# A new *Trypanosoma cruzi* genotyping method enables high resolution evolutionary analyses

**DOI:** 10.1590/0074-02760200538

**Published:** 2021-08-30

**Authors:** Christian Macagnan Probst, Myllena de Fátima Alheiros Dias Melo, Daniela Parada Pavoni, Max Jean de Ornelas Toledo, Tainah Silva Galdino, Adeilton Alves Brandão, Constança Britto, Marco Aurelio Krieger

**Affiliations:** 1 Fundação Oswaldo Cruz-Fiocruz, Instituto Carlos Chagas, Laboratório de Genômica Funcional, Curitiba, PR, Brasil; 2 Fundação Oswaldo Cruz-Fiocruz, Instituto Oswaldo Cruz, Laboratório de Biologia Molecular e Doenças Endêmicas, Rio de Janeiro, RJ, Brasil; 3 Universidade Estadual de Maringá, Departamento de Ciências da Saúde, Laboratório de Doença de Chagas, Maringá, PR, Brasil; 4 Fundação Oswaldo Cruz-Fiocruz, Instituto Oswaldo Cruz, Laboratório Interdisciplinar de Pesquisas Médicas, Rio de Janeiro, RJ, Brasil

**Keywords:** Trypanosoma cruzi, genotyping techniques, high-throughput nucleotide sequencing, trans-sialidase

## Abstract

**BACKGROUND:**

*Trypanosoma cruzi* is an important human pathogen in Latin America with nearly seven million people infected. It has a large degree of genetic diversity, classified into six discrete typing units (DTUs), which probably influences its physiological behavior and clinical manifestations. Several genotyping methods are available, with distinct performance on easiness, cost, resolution and applicability; no method excels in all parameters.

**OBJECTIVES AND METHODS:**

To devise a molecular method for *T. cruzi* genotyping, based on polymerase chain reaction (PCR) amplification of a single target with multiple copies in the nuclear genome by large scale sequencing. We have applied this method to 29 *T. cruzi* isolates, comprising all described DTUs.

**FINDINGS:**

We were able to classify all samples into sub DTU level with high robustness. Evolutionary relationship between DTUs were ascertained, suggesting that TcIII and TcIV DTUs are non-hybrid, and DTU IV is more similar to the common ancestral.

**CONCLUSION:**

As the TS-LSS method is based on a single PCR reaction, comprising several copies of the target, it is probably useful for clinical samples, when the amount of DNA is a limiting factor. As large scale sequencing systems become more common, the TS-LSS method can be increasingly applied for *T. cruzi* genotyping.

*Trypanosoma cruzi* is the causative agent of Chagas’s disease,^1^ an illness that affects nearly seven million people mostly in Latin America, being a significant cause of morbidity, mainly cardiac insufficiency, and mortality, especially when the infection route is oral.^2^ It is a protozoan transmitted to humans by contact of contaminated excreta of triatomine insects with mucosal surfaces or skin lesions, or by non-vectorial mechanisms, as blood transfusion or mother-to-child.^3^ It is estimated that 65 million people live in endemic areas, susceptible to be infected.^4^

*Trypanosoma cruzi* is a genetically divergent organism and several genetic divisions were proposed, depending on the technique used, according with methodological advances, and the last nomenclature consensus subdivides the *T. cruzi* genetic diversity into six discrete typing units (DTU), from DTU TcI to DTU TcVI.^5^ Some authors suggest that the DTU TcI has a significant degree of genetic divergence and could be subdivided into at least four distinct groups.^6^ ^,^ ^7^ ^,^ ^8^ Also, a *T. cruzi* near-clade associated with bats, TcBat, distantly related to DTU TcI, was proposed.^9^

*Trypanosoma cruzi*, as many other pathogen species, follows a pattern of preponderant clonal evolution (PCE), showing limited genetic recombination.^10^ However, the TcV and TcVI DTUs are probably TcII-TcIII recent hybrids and possibly TcIII and TcIV DTUs are TcI-TcII ancient hybrids.^11^ ^,^ ^12^ Active recombination may be a rare phenomenon but it has epidemiological importance, as TcV and TcVI are strongly associated with syntomatic human cases of Chagas’s disease in the southern countries of South America.^5^

Interest in studying the genetic diversity of *T. cruzi*, trying to correlate it with biological (parasite) and clinical (human) phenotypes, is tempting. An initial finding that distinct clinical forms of Chagas’s disease were caused by distinct DTUs started the interest in this field of study.^13^ However, establishing a causal link between *T. cruzi* genetic composition and the disease epidemiology is difficult and this question remains open. This is due to several factors, as the *T. cruzi* diverse host spectrum, the strong association of DTUs with ecological and geographic locations and the complexity of host-parasite interaction, but also to the discriminatory power of current genotyping methods. Several distinct methods, using distinct targets and techniques, were applied for *T. cruzi* genotyping, reviewed in Messenger et al.,^14^ and currently the multilocus sequence typing (MLST) is considered the best method.

Trans-sialidase and trans-sialidase-like (TS) proteins are present in a large number of copies in *T. cruzi*, aproximatelly 1,430 in CL Brener strain, with a large proportion (~48%) as pseudogenes.^15^ It is largely polymorphic, being classified into eight distinct sequence similarity clusters,(16) and only one group (TcTSI) has trans-sialidase activity, where members of other groups are involved in host cell attachment and invasion (TcTSII, for instance).^17^ ^,^ ^18^

Due to the high level of sequence diversity present in the TcTS gene family, we devised a phylogenetic method based on the polymerase chain reaction (PCR) amplification of a multicopy amplicon targeting members of the trans-sialidase family using only one pair of primers and sequence determination by large scale sequencing. This method is highly robust for DTU and sub-DTU assignments, allowing high resolution evolutionary analyses.

## MATERIALS AND METHODS

*Analysed samples and DNA purification* - Twenty stocks covering all six *T. cruzi* lineages (DTUs), as well as *T. cruzi marinkellei*, *T. rangeli* and *T. cruzi* Bat samples, were used for genotyping the parasite through the nucleotide variation on the 5’ extremity of the trans-sialidase multigene family (Table).

A *Leishmania* sample used as a control of cross reaction of PCR was donated by CLIOC collection of Institute Oswaldo Cruz. All *T. cruzi* samples were cultivated in Liver Infusion Tryptose (LIT) at 28ºC. In average, 1 x 10^7^ parasites were submitted to DNA extraction with the QIAamp DNA Mini kit (Qiagen, USA). The DNA concentration was determined by a Picodrop 200 spectrometer equipment (Picodrop, UK).

**TABLE:** *Trypanosoma cruzi s*tocks analysed in the present study, with associated data

Stock	DTU	Isolation organism	Isolation locale	Isolation date	Source
12SF	II	*Homo sapiens*	Bahia, Brazil	~ 1979	C
222	III	*H. sapiens*	Minas Gerais, Brazil	~ 1979	C
3663	III	*Panstrongylus geniculatus*	Amazonas, Brazil	~ 2000	A
4167	IV	*Rhodnius brethesi*	Amazonas, Brazil	~ 2000	A
AM14	IV	*H. sapiens*	Amazonas, Brazil	2007	C
CanIII cl1	IV	*H. sapiens*	Pará, Brazil	1968	C
CL Brener	VI	*Triatoma infestans*	Rio Grande do Sul, Brazil	1963	A
Colombiana	I	*H. sapiens*	Colombia	< 1964	A
D8	I	*Didelphis marsupialis*	Rio de Janeiro, Brazil	1996	B
Dm28c	I	*D. marsupialis*	Guárico, Venezuela	1976	A
G	I	Opossum	Amazon, Brazil	< 1983	A
LL014	V	*T. infestans*	Chaco, Argentina	~ 2000	A
Peruana	VI	*H. sapiens*	Peru	1966	A
SO3 cl5	V	*T. infestans*	Potosi, Bolivia	NA	C
Tulahuen cl0	I	*H. sapiens*	Tulahuen, Chile	< 1950	A
Y	II	*H. sapien*s	São Paulo, Brazil	1953	A
CP300	VI	NA	NA	NA	A

Source: (A) ColProt FIOCRUZ collection; (B) ColTryp FIOCRUZ collection; (C) Dr Max Jean de Ornelas Toledo, UEM-PR.

*Selection of the gene target* - Considering the high number of copies, the trans-sialidase gene family was selected as a first ubiquitous target. The set of sequences was retrieved from the CL Brener genome data at TriTrypDB, with the following parameters: ‘trans-sialidase’ key, not pseudogene and size larger than 2 kb (n = 500, average sequence size 2,618 nucleotides, comprising members of all TcTS groups). An amplicon size of 200 nucleotides was pre defined, as suitable for large scale DNA sequencing protocols, and oligonucleotides forward (5’-CTAATCGCTACTGTGAAATT-3’) and reverse (5’-GCCGGG ACATGTTGGGCCTC-3’) were predicted, aiming to be identical to at least 60% of the selected set. The CL Brener sequences TcCLB.506459.230, TcCLB.506537.200, TcCLB.508853.20 and TcCLB.509817.40 are matched perfectly by both primers. This target region is located in the 5’ end of TS genes, with aproximatelly one third consisting of the 5’ UTR region and the remaining comprising the CDS N-terminal end.

*PCR amplification* - A standard PCR reaction to amplify the TS amplicon was set up as follows: 0.2 µM of each primer, 1x PCR reaction buffer, 2 mM MgCl_2_, 200 µM each dNTP, 100 ng DNA, 1 U Taq polymerase, to a final volume of 50 µl. The cycling was 94ºC for 5 min, followed by 40 cycles of 94ºC for 10 s, 56ºC for 30 s and 72ºC for 30 s, with a final extension step of 72ºC for 10 min [Supplementary data I (Fig. 8)].

Sequencing assays

*Prototype testing* - To establish the reliability of the trans-sialidase target for phylogenetic analysis and to assess the influence of technical variability in the final results, we have selected one *T. cruzi* stock for each DTU, and performed two separated PCR amplifications of the same DTU DNA sample according to the procedure described above. After the amplifications, each sample was processed independently and sequenced in the same sequencing run. We have followed the manufacturer’s instructions from the Ion Xpress Plus Fragment Library Kit (P/N 4471269), using a Ion 314 chip v2 (P/N 4488144) with a Ion PGM Sequencing Kit v2 (P/N 4482006).

*Phylogenetic assay* - After accessing the experimental variability incorporated by separated technical procedures, we performed a phylogenetic assay where at least two samples per DTU were sequenced. All samples that were sequenced in the prototype assay were sequenced again, from a distinct PCR product. The samples were sequenced in two distinct sequence runs [Supplementary data I (Table III)], using the manufacturer’s instructions from the Ion Xpress Plus Fragment Library Kit (P/N 4471269), using a Ion 314 chip v2 (P/N 4488144) with a Ion PGM Sequencing Kit v2 (P/N 4482006).

All sequencing was performed at the Large Scale Sequencing Facility (RPT-01G), Instituto Carlos Chagas, FIOCRUZ-PR.

Bioinformatics analysis

*Read filtering* - The reads produced were filtered using the BLASTn algorithm against a trans-sialidase prototype sequence (accession number XM_812522, positions 116 to 313). Only those reads that had a complete match (i.e., from the start to the end of the prototype sequence, excluding the primers, named *reads passed, RP*) were selected for posterior analyses [Supplementary data I (Table I)]. In the filtering process, anti-sense reads were reverse complemented.

*Read clustering* - To obtain a general quantification of diversity, we have compared all RP against each other using the BLASTn software. The resulting similarity file was analysed by the MCL algorithm,^19^ with default parameters, using different similarity thresholds for filtering the BLAST results before clustering.

*K-mer analysis* - Filtered but non-corrected reads were analysed with the software FFP-Feature Frequency Profile Phylogeny,^20^ to extract all 20 nt k-mers present in all reads from all samples, and to create phylogenetic analyses based on k-mer frequency. The phylogenetic construction based directly on FFP software results was done with Jensen Shannon Divergence (JSD) distance and neighbor joining method. The k-mer counting was normalised by transforming the total count of each sample to be equal to the count of the sample with less reads.

*Analyses with distinctive k-mers* - Those k-mers that have a ratio between the highest and lowest sample counting higher than 20 (K20) or 100 (K100) were selected for further analyses. For the quantitative phylogenetic analyses, an Euclidean distance was calculated on normalised k-mer counting, with 1,000 bootstrap replicates, and a phylogenetic tree was constructed with the neighbor joining method. We used the Phylip package to perform the neighbor joining method,^21^ iTOL for tree drawing,^22^ and the newick utilities for general processing.^23^

*Self organising maps (SOM) clustering of k-mer distribution* - The software Expander^24^ was used for SOM clustering of all k-mers for the K20 and K100 sets. We used a SOM grid of 20 rows by 100 columns (at most, 2,000 clusters) and the maximum number of allowed iterations.

## RESULTS

*Potential diversity of the TS target* - Aiming to evaluate the trans-sialidase target diversity *in silico*, we identified all CL Brener genome fragments that were similar to the last ten nucleotides of both forward and reverse primers by BLAST analysis, allowing one possible mismatch not in the last 3’ primer nucleotide. This assumption was considered as a proxy for the resulting PCR amplification and sequencing that would be obtained by the TS-LSS method, before a more detailed analysis based on real data from distinct *T. cruzi* strains were obtained (see below), as it is not possible to predict exactly the resulting amplification profile of this diverse gene family.

**Fig 1:**
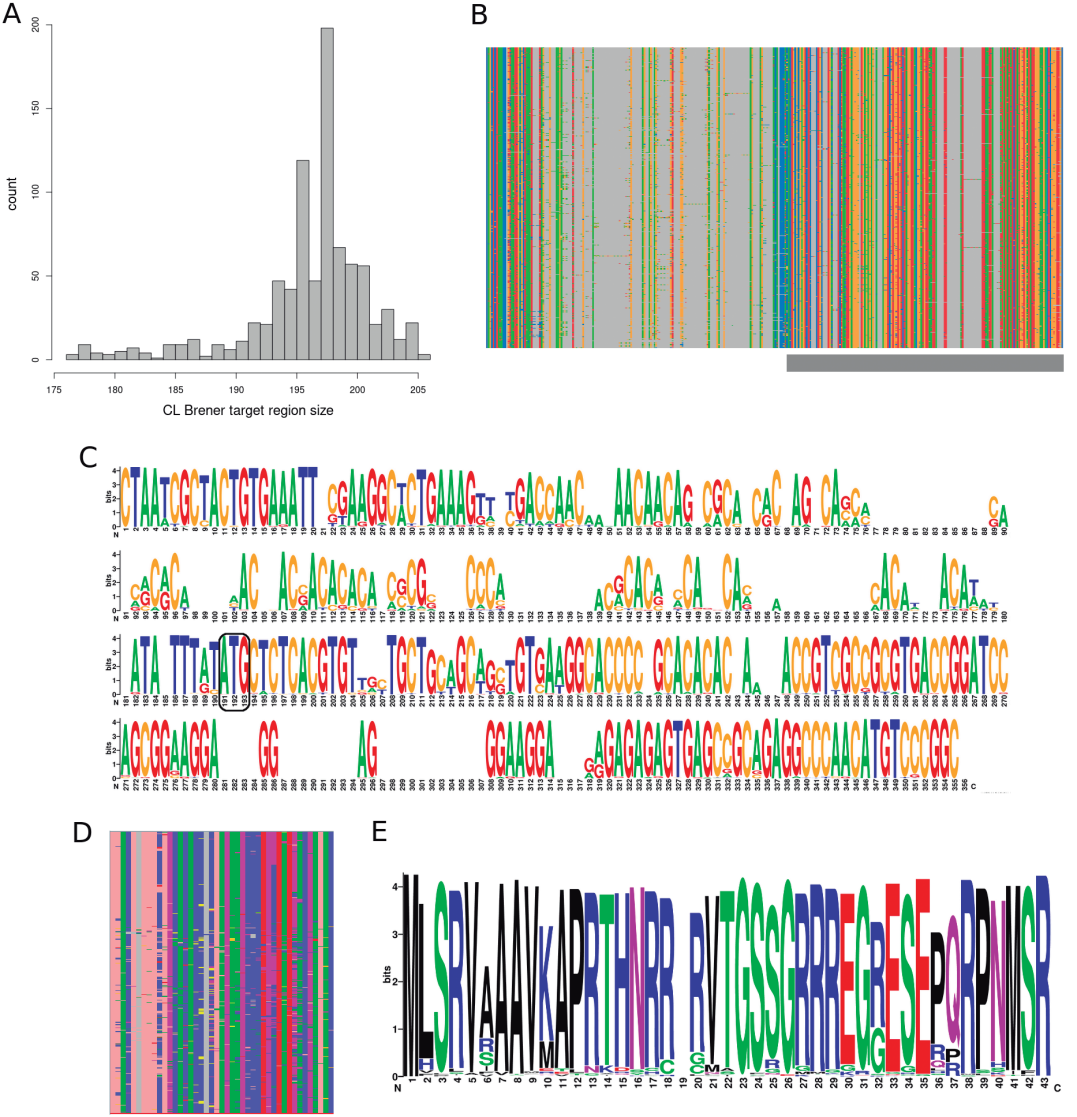
diversity of the trans-sialidase potential targets in CL Brener genome assembly. (A) Fragment size distribution of all trans-sialidase copies identified. (B) Overview of nucleotide alignment of all trans-sialidase targets identified, where each nucleotide has a distinct colour and gaps are represented as gray; the gray box in the bottom represents the CDS; (C) Logoplot of the alignment depicted in B, where the height of each position represents its conservation; the CDS start codon is identified by a rounded box; (D) Overview of the amino acid alignment of all trans-sialidase targets identified with an open reading frame, where each amino acid has a distinct colour and gaps (indels) are represented in gray; (E) Logoplot of the alignment depicted in D, where it is possible to observe that some positions have non-synonymous mutations.

A total of 870 sequences were identified with the above criteria, comprising also pseudogenes, whose mean size was 196.2 nucleotides and with two distinct modes at 196 and 198 nucletides (Fig. 1A). The total GC content of these sequences is 56.6%, higher than the average GC content of the *T. cruzi* genome, that is 51.7%. The nucleotide alignment of these sequences (Fig. 1B) shows that there are several long gaps, generally present in just one sequence, more frequently in the 5’ UTR region but also in the protein coding region; variant nucleotide positions are localised all along the sequence (Fig. 1B-C). From this sequence set, it was possible to identify an open reading frame compatible with the amino terminal targeted area for 784 sequences (90.1% of the initial set), comprising many non-synonymous SNPs (Fig. 1D-E), including positions within the signal peptide. These results, altogether, show that the putative amplifiable set of CL Brener sequences have a large ammount of diversity, indicating its suitability for phylogenetic inference.

We searched for homopolymeric nucleotide runs in the TS set identified in CL Brener, as the Ion Torrent technology has an increased error rate for this trait, but very few examples larger than three nucleotides were identified, restricted to a small fraction of the TS set, not occuring as a trait shared by most TS genes (data not shown). Hence, we assume that, although this type of platform-dependent error will occur, it will probably have very low impact in the phylogenetic results obtained (see below).

*Data generated* - We have produced three runs of Ion Torrent sequencing [Supplementary data I (Tables I-III)], covering 29 different samples of *T. cruzi*, related to 20 different biological stocks (strains, clones, subspecies). For six stocks, a technical duplicate PCR was produced (Dm28c, Y, 222, 4167, Bug2148 and CL Brener) and sequenced in the same run; for four stocks, a technical replicate of both amplification and sequencing was produced (Dm28c, 222, 4167, CL Brener). At least nine thousand reads were generated per sample, with a read mode of ~200 nucleotides, the size of the PCR amplicon.

After sequencing, we selected those reads that had a complete sequence, excluding the priming region. In average, 50% of the generated reads passed this criterion.

**Fig. 2:**
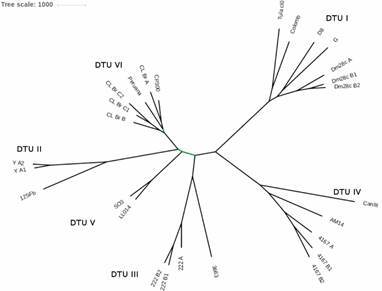
phylogenetic tree based on the K20 set. All branches showed 100% bootstrap support, with the exception of the branches depicted in green (from 86.2% to 86.7%).

*Technical reproducibility* - As the TS-LSS method is based on a single PCR annealing element that has multiple sites in the *T. cruzi* genome, this can lead to distortion in the sequence representation as distinct internal sequences may have different amplification kinetics. We have initially conducted a pilot assay to assess technical reproducibility, selecting one representative *T. cruzi* stock for each DTU, performing a separated amplification and sequencing procedure [Supplementary data I (Table I)], generating ~3 Mb per sample. In general, the obtained results were very reproducible [Supplementary data I (Table II)], especially when focusing on the identified k-mers [Supplementary data I (Figs 1-2, see below)]. The reproducibility is similar even when dealing with hybrids, more complex DTUs, as TcVI. We were not capable of evaluating the technical reproducibility of TcV, as the Bug2148 sample sequenced was identified by the method as pertaining to DTU I, being suggestive of a mislabel or a mixed sample, but as this DTU is very similar to TcVI in sequence diversity, hybrid origin and putative ancestral DTUs, we assume that its technical reproducibility will have a similar pattern. It is interesting to notice that the comparison between TcI and TcII, for instance, has a significant higher level of diversity than the technical replicates, seen as a greater dispersion of the k-mer counts, what reinforces that the biological information is larger than the technical error related to PCR amplification, sample processing and sequencing [Supplementary data I (Fig. 1)].

*K-mer occurrence* - The complexity of the read sequences is very high, including the genetic diversity, that is informative for DTU assignment and evolutionary studies, but also the sequencing errors, that blurs the identification of rare target variants, probably more informative for a detailed phylogenetic analysis. In order to simplify the analytical pipeline, we used an alignment-free, k-mer occurrence phylogenetic analysis of the sequencing results, for the pilot (Supplementary data II) and for the phylogenetic assay (Supplementary data III), instead of relying in the alignment of the complete sequence.

When analysing the total set of sequenced samples (n = 29), we have identified 1,049,005 distinct k-mers. Very rare k-mers are probably sequencing errors and when we apply distinct thresholds based on their occurrence in the samples, a great reduction in the number of identified k-mers is seen, as expected [Supplementary data I (Table IV)]. As an example, only 4,400 k-mers were identified with a normalised read count of at least one in all samples (0.4% of all k-mers) and only 531 k-mers were identified with a normalised read count larger than 100 in all samples (0.05%). It is important to emphasise that the read count has no absolute frequency interpretation per se and is an arbitrary metric influenced by sequencing depth and normalisation procedures. However, its relative interpretation is straightforward, giving us a sense of intra and inter-stock diversity for the trans-sialidase target.

*Identification of specific patterns* - For the DTU assignment and phylogenetic studies, it is important to work with k-mers that are informative, having a great degree of distinctiveness between samples and/or DTUs [Supplementary data I (Table V)]. To identify informative k-mers for phylogenetic analysis, we have produced two distinct k-mer sets, aiming to reduce the number of analysed elements, but retaining informativeness. First, a selection of k-mers based on a prior phylogenetic classification of the selected samples into DTUs [Supplementary data I (Table VI); Supplementary data III, DTU specific sheets]; and second, a more generic selection of k-mers that have greater distinction power between samples, without taking in consideration previous knowledge regarding their classification into DTUs (K100, 100-fold difference between the lowest and highest normalised read count per sample, Supplementary data V; K20, 20-fold difference, Supplementary data VI).

*DTU-aware analysis* - We performed DTU-aware phylogenetic analysis, where the k-mer count was transformed into a binary trait (+/-) and only those k-mers whose DTU-aware discriminatory power, or, in other words, the ratio between the less frequent read count in a stock from a specific DTU and the more frequent read count in a stock from all samples from the other DTUs, was larger than 5-fold were selected. In total, 1,803 k-mers passed this criteria (Supplementary data VI; TcI, n = 508; TcII, n = 23; TcIII, n = 32; TcIV, n = 686; TcV, n = 97; TcVI, n = 492). TcI and TcIV have the largest number of discriminative k-mers, and the majority of these k-mers have high ranking, i.e., a high frequency in the amplicon pool and therefore are less prone to be influenced by sampling effects; although the TcVI has a high number of discriminative k-mers, most of these k-mers has lower ranking; this is not unexpected as, due to its hybrid origin, specific k-mers are probably very recent and not present in many copies in the genome; the TcII and TcIII have fewer discriminative k-mers and this is mostly explained by their participation in the hybridisation event(s) giving origin to TcV and TcVI. The high number of specific TcVI k-mers is unexpected and is probably explained by a low genetic heterogeneity between the analysed TcVI stocks. When including more TcVI samples, with a higher genetic diversity, we presume that the number of specific k-mers will be similar to that identified in TcV.

The DTU-aware analysis illustrates that there is enough phylogenetic information to clearly discriminate the stocks in DTUs, in a simpler analytical procedure, based on specific markers. Besides, this type of analysis can be used to detect mixed populations or contaminated cultures, if involving distinct DTUs. It is expected that when increasing our evaluation of intra-DTU diversity by sampling a larger number of stocks in the future, the number of DTU specific k-mers will decrease, converging to a robust, stable representative set, that can be used for analysis in a cladistic framework.

*General analysis of k-mers* - For the K100 and K20 set, we have selected 1,697 and 17,996 k-mers, respectively [Supplementary data I (Table VII)]. In Supplementary data I (Fig. 4), the locations of the informative k-mers are displayed and they are distributed all along the amplicon sequence, both in the 5’UTR and CDS regions.

When looking at the clusters identified by the SOM algorithm (Supplementary data IV-V, for the K100 and K20 datasets, respectively), it is possible to see that the vast majority of k-mers are DTU-related, i.e, shared by the members of the same DTU (Supplementary data VII, where the DTU enrichment pattern of each cluster is described). As expected, most of the TcV and TcVI enriched k-mers are also identified in TcII or in TcIII, or both; TcI has a higher proportion of clusters with specific or enriched k-mers; for TcIII enriched k-mers, we can see a small degree of sharing with TcI, but the same is not so apparent for TcIV, as it shares its k-mers with other DTUs but TcI.

As expected, the separation between TcV and TcVI is less clear, but some many small clusters are very informative (for instance, K100, clusters 14, 23, 26, 28, 36, 39, 43, 44, 52, 62, 63, 65). In general, these clusters have k-mers that are also found in TcII or TcIII, but they are more frequent for TcV or TcVI, and can be discriminatory when considering their frequency. This illustrates how the evolutionary signal differentiating these two DTUs is weak; nevertheless (see below), the TS-LSS method has sufficient power to clearly separate them, distinct from the majority of *T. cruzi* genotyping methods that are very limited to discriminate these two DTUs.

*Genetic relationship between T. cruzi samples* - We have used the information present in the K20 and K100 sets to analyse the phylogenetic relationship of the *T. cruzi* samples, excluding *T. c. marinkellei* and TcBat from this analysis due to the fact that, as they are highly divergent (Supplementary data III), it is easy to identify them as distinct phylogenetic entities.

In Fig. 2, the phylogenetic relationship between the *T. cruzi* samples constructed with the K20 set is shown [for K100, see Supplementary data I (Fig. 4)]. The known phylogenetic relationships between *T. cruzi* DTUs are readily identified, even for those DTUs that are more difficult to separate, as the hybrid TcV and TcVI.

We have selected two levels of distinct informativeness, aiming to analyse what would be more appropriate for identifying the phylogenetic relationship between the samples: if the use of a larger set of informative k-mers (K20) or a more restricted set with higher discriminatory power (K100). Considering the granularity, bootstrap confidence and intra-DTU divergence assessment, the K20 set performed slightly better. This is expected, as a 20-fold difference between k-mers retains a high discriminatory power and the total number of k-mers used for the analysis is significantly larger (~10-fold) than that for the K100 set.

Although the trees showed strong bootstrap support for the DTU attribution, we can see that technical variation (PCR and sequencing biases) has an impact in the resolution of the current method. This can be seen at different levels: (i) the stocks Dm28c (samples Dm28c B1, B2), Y (Y A1, A2), 222 (222 B1, B2), 4167 (4167 B1, B2) and CL Brener (CL Br C1, C2) that were sequenced in the same sequencing batch from two distinct PCR amplifications performed at the same time from the same DNA sample. They all clustered together in the quantitative analysis, but there was a small distance between these replicates, what is probably related to stochastic processes occurring during PCR amplification, especially with saturation occurring with the large number of cycles applied, but also from sequencing errors; (ii) the samples Dm28c (A, Bx), 222 (A, Bx), 4167 (A, Bx) and CL Brener (B, Cx) that were produced in different sequencing batches from distinct PCR amplifications performed at different times from the same DNA sample. They also clustered together, as expected, but the distances are slightly larger than what we see in (i), showing that sequencing batch effects also occurs. Nevertheless, all stocks were clearly identified as distinctive entities, reinforcing the fact that the technical variation (error) is smaller than the biological information (genetic diversity) discriminating the stocks analysed here. Procedures to decrease technical variability, both pre and post sequencing, are being integrated in the TS-LSS method, for future updates of this method.

*Phylogenetic information in the higher ranking k-mers* - The majority of the discriminatory k-mers (in K20, K100 and DTU-aware sets) are not very common. Generally, the most common k-mers have lower technical variability [Supplementary data I (Fig. 1)], being less susceptible to sampling bias, aggravated by sequencing errors. In order to see if the higher ranking k-mers, although less discriminatory, have retained enough phylogenetic information for a correct DTU assignment, we have selected only the more common k-mers with at least two-fold normalised count ratio (equivalent to a K2 set, but containing only those k-mers with high frequency). Using the 1,000 most frequent k-mers, it is possible to reconstruct a phylogeny very similar to the previous trees [Supplementary data I (Fig. 1)]. The only difference is that the 3663 and 222 stocks (TcIII) are not in a common branch, but close together. This last result could be explained by the large phylogenetic distance between these two stocks, and when using the 2,000 most frequent k-mers [Supplementary data I (Fig. 10)], these two stocks branch together.

Nevertheless, the use of only the most frequent k-mers has enough phylogenetic information to discriminate the stocks. One major consequence of this analysis is that we can obtain high quality evolutionary information using lower sequencing coverage, decreasing the experimental costs.

*Sequence diversity of TcBat DTU and T. c. marinkellei subspecies* - TcBat is probably a distinct DTU that is found not exclusively in bats;^9^ ^,^ ^25^ *T. c. marinkellei* is a bat-associated parasite, regarded as a *T. cruzi* subspecies. TcBat was the DNA sample that showed the smaller proportion of mapped, passed reads (17.9%) and this could indicate a distinct trans-sialidase set whose sequence(s) was not amplified by the PCR primers. However, as there was only one TcBat sample, it is not possible to exclude the possibility of technical issues for the low proportion of mapped reads. This is the most probable scenario, as for instance *T. c. marinkellei,* a more distant related entity to the other *T. cruzi* stocks, had one of the highest proportion of mapped, passed reads (74.7%).

In relation to the discriminative k-mers, TcBat and *T. c. marinkellei* have sets with similar size and discriminatory power as the *T. cruzi* DTUs. However, *T. c. marinkellei* has k-mers with the highest discriminatory power (the best with 169-fold difference), what is expected due to its more distant genetic relation with the other *T. cruzi* stocks.

The phylogenetic tree using the K20 set including the TcBat and *T. c. marinkellei* samples is shown in Supplementary data I (Fig. 11). By using *T. c. marinkellei* as an outgroup, TcIV is the first to diverge from all other DTUs, a pattern that is not seen with other markers, where generally TcI is the first to diverge. The TcBat sample diverges after the TcI, in a common branch with the other DTUs, a different evolutionary origin from that previously described.^25^

*Specific patterns restricted to each sample* - To analyse the occurrence of sample specific k-mers, instead of DTU specificity, aiming to assess more recent shifts in trans-sialidase repertoire, we have selected those k-mers that were enriched at least two fold with a normalised count higher than 10 in a specific sample when compared to all others (Supplementary data VIII). All stocks that belong to non-hybrid DTUs have a relatively large number of enriched k-mers, ranging from 158 to 600. The hybrid TcV and TcVI DTUs have a smaller number of enriched k-mers, specially for TcVI. For this last group, the similarity between the four constituent stocks is extremely high, what could explain the smaller number of sample-specific enriched k-mers.

*Bioinformatics analyses of trans-sialidase repertoire* - To evaluate the capability of the distinctive k-mers identified by the TS-LSS method to classify *T. cruzi* samples *in silico*, we analysed all *T. cruzi* genome assemblies produced with Pacific Biosciences long read technology to verify if the DTU specific patterns were corroborated. TcDTU I Dm28c (MBSY01) and Brazil (WNWZ01), TcDTU II Y (WNWY01), TcDTU V Bug2148 (NMZN01) and TcDTU VI TCC (PRFC01) genome assemblies were retrieved from NCBI and submitted to the same analysis performed for CL Brener strain. We have identified 703, 605, 576, 787 and 840 TS targets for Dm28c, Brazil, Y, Bug2148 and TCC strains, respectively.

**Fig. 3:**
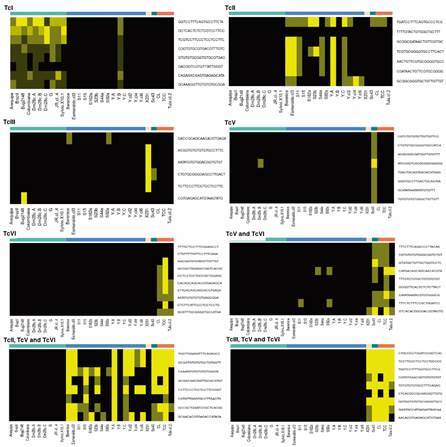
heatmap representation of selected DTU-specific k-mers and their identification in all genome assemblies available at NCBI. Selected k-mers are represented in each row and the genome assemblies are in the columns; the black colour represents absence of that k-mer in the trans-sialidase identified *in silico* in each genome assembly, and distinct shades of yellow give a estimative of how many copies share that k-mer.

When searching for the top most discriminative k-mers for each one of the six DTUs, the results were as expected, with a high degree of concordance even for donor DTUs, as TcII (Fig. 3). A surprising finding was that the Bug2148 genome assembly data was also classified as TcI and not as TcV. As described in the prototype assay, we also had this result with a Bug2148 sample that was included in the present work. These results are indicative of a possible sample mislabelling, previous to studies conduct by two distinct groups. Another possibility is that Bug2148 is a mixed stock, with a predominant proportion of a TcI genotype.

We have extended this analysis to all *T. cruzi* genomes that are available at the NCBI depository [Supplementary data I (Table VIII)], and excluded the genome assemblies for the B.M.Lopez, S154a and Ikiakora strains as the number of identified trans-sialidases was very low. Most of the DTU specific k-mers are identified in the genome datasets in a coeherent pattern, as expected (Fig. 3), but there are few discrepancies, generally when analysing k-mers that distinguish DTUs II, III, V and VI. Unfortunately, there is no DTU IV genome assembly available until now, and just one for TcDTU III, what limits our inference in relation to these DTUs. The discrepancies observed can be due to several factors, as non-amplification of some tran-sialidase genes, incompleteness of genome information and k-mers present only in strains that were not previously analysed. As stated before, we intend to increase the number of samples assayed with the TS-LSS method, taking advantage of its great potential as a tool tailored for the analysis of a large number of samples, to provide a better picture of the genetic diversity of *T. cruzi* as well as increasing the robustness of DTU assignment. It is also very important to emphasise that an analysis based only on the presence or absence of specific k-mers is more limited than using the whole set of distinct identified k-mers and their frequencies, as we have done before.

Another interesting result of this analysis using the TS-LSS target amplicon for DTU validation in an *in silico* analysis of genome assemblies is that it serves as a genome assembly quality measurement. For instance, it is very clear that genome assemblies produced by long read technologies (both Pacific Biosciences and Oxford Nanopore) have a larger number of trans-sialidases targets identified. The larger number of *in silico* identified trans-sialidase targets for long read assemblies is clearly seen when looking at those strains whose genome was assembled more than once, with different technologies, as Dm28c, Y and CL.

Although it is expected that more contiguous assemblies of repetitive genomes will yield better representations of genome structure, it is usually assumed that short read technologies, with enough coverage, will comprise most, if not all, genomic information. Our results show that this is not the case. One possible explanation is that when applying post-assembly filtering, those contigs containing repetitive, complex structure sequences (as for the trans-sialidase targets) are excluded from the final assembly. This is especially true for the B.M.Lopez and Ikiakora genome assemblies, where the number of identified trans-sialidases targets are very low (12 and 0, respectively), and the authors stated in the Genbank genome entry that they are partial, with many repetitive regions lost in the assembly proccess.

Another result of this analysis was the identification that the genome assembly NMZO01 of the Y strain (DTU TcII) is in fact from a TcI strain; there are other five genome assemblies for the Y strain, all compatible with a TcII classification, and the NMZO01 assembly is clearly distinct, grouped in DTU TcI. This result reinforces the capability of the TS-LSS method to precisely identify DTU and also that DTU determination is of utmost importance before conducting large, expensive projects on *T. cruzi* samples.

## DISCUSSION

The analysis of *T. cruzi* diversity is still a topic of great interest, as Chagas’s disease continues to be a significant public health issue;^4^ besides, it has not been established a strong evidence of *T. cruzi* genotype association with clinical or biological manifestations.^14^ Another interesting topic that needs powerful analytical methods is the evolution model of *T. cruzi*, involving clonality and occasional genetic exchange through hybridism.^10^ ^,^ ^26^

Several typing methods are available for *T. cruzi,* tailored for clinical sample or strain genetic diversity analysis, and this focus of research has always had a strong association with the technical developments available at a specific time.^14^ Currently, the more useful methods are divided in two broad categories, focusing either on easiness of implementation and low cost (size of PCR fragment, RFLP) or on high information content (microsatellites). Regarding these parameters, MLST is an intermediate approach, and is considered the actual gold standard for *T. cruzi* genotyping.^14^

Here, we have described a method that attempts to incorporate technological advances to obtain high information content with ease of production for multiple samples. The positive points of our method are a single, PCR-based target needing smaller DNA amounts; broad coverage of evolutionary patterns, as although based on a single target amplicon, the trans-sialidase target has many amplifiable copies, each one able to tell a different evolutionary story; the data obtained is at the most informative level, nucleotide sequence determination; and it has high multiplexing capability. One possible limitation is the fact that the evolution model of the trans-sialidase target is complex, being a multigene family involved in direct contact with the host and, hence, has a larger degree of diversification, selective pressure and also homoplasy.^16^ ^,^ ^27^ ^,^ ^28^ ^,^ ^29^ However, the results presented here suggest that we can have distinct markers (sequence tags) that allow the identification of ancient and recent genetic differentiation events, in spite of more complex evolutionary scenarios directed by functional aspects.

Another potential negative aspect of this method is the fact that it is based on the PCR amplification of a multisequence target and artifacts can appear, including also sequencing errors, modifying the genetic profile of a sample.^30^ This is illustrated in this study by the variation observed for technical replicates of the same sample, even when they were sequenced together. Although this reinforces the fact that we have to be cautious about the resolution level attained, in general the biological variation, that is of interest, is significantly higher than the technical error created by PCR, what is evidenced by the clear delimitation of each stock in the phylogenetic trees. A broader panel of *T. cruzi* samples will allow us to quantify more precisely the degree of separation that can be attained with this method; besides, a decrease in the number of PCR cycles helps to avoid saturation and plateau effects that can have a significant influence in this bias. These are aspects that we will analyse in the near future.

We have used the Ion Torrent technology^31^ to produce the sequence data. This technology is based in semiconductor detection of protons released when nucleotides are incorporated during DNA synthesis. The error rate varies according to GC content, but it was estimated to be 1.8% in general, higher than the 0.4% error rate estimate for the Illumina platform, that is the dominant sequencing technology nowadays.^32^ Due to its detection method, the Ion Torrent platform has a higher error rate for homopolymeric runs, specially larger than eight nucleotides.^33^ However, there are very few homopolymeric runs larger than four nucleotides in the trans-sialidase sequence set, indicating that an increase in error rate by using the Ion Torrent technology is probably not significant. Nevertheless, we will evaluate the TS-LSS method in other platforms in the near future.

When dealing with high throughput sequencing technologies, three major drawbacks are raised: high equipment costs, high reagent costs and bioinformatics skills needed. Regarding high equipment costs, it has been true in the past, but nowadays we have equipments that have costs equivalent to or cheaper than PCR machines, a common molecular biology laboratory equipment. The recent release of the Oxford Nanopore MinIon and Illumina iSeq devices illustrates that equipment cost is not more a significant issue regarding large scale sequencing. In relation to reagent costs, they represent a significant burden when taken by nominal value, but obviously a high throughput sequencing method will focus on multiplexing, enabling analysis of a large number of samples, and the cost per sample will be very low. As such, the current method is not tailored for a single or few samples, but enables a very cost effective, automation prone, way to analyse a large number of samples.

In fact, multiplexing is one of the major strength of a large scale sequencing assay, as it is possible to sequence thousands of samples using the current proposed protocol, at a very low cost per sample, enabling large genotyping efforts to study *T. cruzi* genetic diversity, especially in scenarios where high resolution is needed, as clinical association studies.

In other words, the focus changes from assaying a few strains to assaying a more representative set, similar to what is occurring in microbial genomics.^34^ Enabling high resolution genotyping of thousands of samples at low per sample cost stresses the steps of sample management, DNA extraction and sample addressing. The fact that the TS-LSS method relies on just one PCR reaction facilitates the processing required to obtain adequate material for sequencing.

Finally, although the bioinformatics analyses presented here are diverse, they represent more the wealth of distinct views that are possible with this kind of data rather than bioinformatics complexity per se. A computational pipeline can be easily implemented, with low processing and memory requirements, allowing non-specialised users to obtain high quality phylogenetic information. There is a significant requirement for data storage, but this in fact is also the main strength of this method, as it provides a high degree of information.

Until now, there is no *T. cruzi* genotyping method that has a great degree of resolution based on a single reaction target and large scale sequencing. Here, we devised a method with just one single amplicon, that due to its high copy number, enables high resolution genotyping. The TS-LSS method has the ability to clearly separate the current devised six *T. cruzi* DTUs as well as providing sub-DTU classification, illustrated, for example, by the sub clustering of TcI strains and the demonstration of large intra-DTU genetic distance between the TcIII and TcIV DTU strains. This method, as it is based in a large quantity of high resolution information, has the potential to identify mixed infections, also providing an estimate of the mixture proportion, although we have not directly tested this point in the present work.

Regarding *T. cruzi* evolution, the identification of several TcIV specific clusters that are not shared with TcI or TcII (for instance, K100, clusters 10, 11, 18, 22, 23, 37, 40, 49, 58, 72) is interesting, as the evolutionary origin of TcIV is controversial, if from one hybridisation event generating the ancestral lineage of TcIII and TcIV,^12^ or if it represents a distinct lineage whose similarities with TcI and TcII are reminiscent of that existing in a common ancestor.^35^

Our results suggest that TcIV has an ancestral origin, due to the presence of these specific clusters, also illustrated in Supplementary data VI, where many DTU-specific k-mers of relative high frequency are found in TcIV, as well by the low number of discriminative k-mers shared with TcIII stocks.

Regarding TcIII, it has also several specific clusters (for instance, K100, clusters 3, 4, 7, 8, 12, 15, 25, 26, 28, 30, 31, 39, 43, 60, 71), generally shared with TcV and TcVI due to the recent occurence of the hybridisation event.^11^ A few TcIII specific clusters, with low number of k-mers, are also evidenced (K100, clusters 35, 41, 51). These results suggest also a non-hybrid origin for TcIII, what has been proposed by other authors,^35^ ^,^ ^36^ ^,^ ^37^ as its sharing of informative k-mers with TcI and TcII is very restricted. In this sense, the TcI, TcII, TcIII and TcIV would be ancient lineages. Interestingly, generally the few k-mers that are shared between TcIII and TcIV (for instance, K100, clusters 5, 6) are much less frequent, or even absent, in TcV and TcVI, suggesting that this specific gene subset was lost after the hybridisation event or, less probably, was not present in the TcIII parental stock, representing a modification of the trans-sialidase repertoire.

It is also important to emphasise that trans-sialidases were previously studied in *T. cruzi* stocks aiming primarily to analyse its profile regarding the phylogenetic conservation of the enzymatically active TS (aTS) and a lectin-like TS (iTS).^36^ When analysing the sequences obtained, it was seen that TcI, TcIII and TcIV are more related and that these two last DTUs are probably not hybrid. We have observed the same results with our method, with strong bootstrap support.

Although interesting, these analyses must be taken with caution due to the functional role of trans-sialidases, as sequence divergence caused by positive selective pressure could be a possible explanation for these patterns. Nevertheless, if that is the case, the distinct ecological niches that created the general diversification of *T. cruzi* DTUs had a very strong effect in trans-sialidase diversification.

Regarding TcBat, it is probably a distinct DTU that is found not exclusively in bats^9^ ^,^ ^25^ and, since its first description, their isolates are clearly separated from other *T. cruzi* lineages. Further studies, based on diverse markers, established that TcBat is indeed a distinct DTU, able to infect other mammals, including humans.^25^ ^,^ ^38^ ^,^ ^39^ ^,^ ^40^ ^,^ ^41^ It was described that TcBat was sister to TcI and more closely related to TcIII than TcIV, being TcII the first DTU to diverge in the phylogenetic analysis. Our results suggest a different scenario, where TcIV is the first DTU to diverge and TcBat is more closely related to TcIII than TcI.

*Trypanosoma cruzi marinkellei* is a subspecies of *T. cruzi* that infects exclusively bats, not infecting other mammals^42^ ^,^ ^43^ and whose divergence from *T. cruzi cruzi* is estimated to have occurred around 6.5-8.5 MYA.^11^ ^,^ ^44^ The trans-sialidase gene content of *T. c. marinkellei* is similar to that of TcI^45^ ^,^ ^46^ and similar to that of the other *T. cruzi cruzi* DTUs, although some k-mers showed the highest discriminatory scores, representing a trans-sialidase gene set that was expanded in *T. c. marinkellei* (Supplementary data III).

Trans-sialidases have a faster evolution rate than usual markers used for *T. cruzi* genotyping, being more informative for studies that need identification of recently emerging phylogenetic entities. However, the information diversity was also able to separate ancient entities, as the current DTU identification. In fact, the composite nature of the sequence repertoire that is amplified represents a plethora of distinct elements with evolutionary differences: first, the amplified region, comprising both a noncoding and protein coding sequence; second, the occurrence of distinct entities as faster evolving pseudogenes, positive selected regions and potentially trans-sialidases with a more constrained evolution due to major, more specific functions.

With the lowering of sequencing costs and the more extensive availability of large scale sequencing equipment, the genetic divergence of *T. cruzi* stocks could be assayed at the highest informative level, the complete genome sequence. However, a targeted single reaction method, as the one presented here, is an interesting alternative, as with the same budget it is possible to obtain a strong phylogenetic information for a larger number of samples, as for instance clinical samples of chronic patients, with low parasitaemia levels.

In summary, we have described here a method based on a single amplicon derived from a multigene family (TS-LSS) that can provide high evolutionary resolution for the genotyping of large number of samples at a very reasonable cost. This method was able to classify *T. cruzi* samples with sub DTU resolution and also to provide insights regarding the evolutionary history of DTUs whose phylogenetic position is more complex.

Additional Information

Data availability

The reads used for this paper are available at the Short Reads Archive (SRA) database, with the accession ID SRP140704 (https://www.ncbi.nlm.nih.gov/sra/ SRP140704). All other informations are in the supporting information files.

Supporting information

Supplementary data I - Additional general information (.pdf format);

Supplementary data II - K-mers identified in the pilot study (.xls format);

Supplementary data III - K-mers identified in the phylogenetic study, also containing the DTU-enriched k-mers list (.xls format);

Supplementary data IV - Heatmaps of SOM clusters of the K100 set (.pdf format);

Supplementary data V - Heatmaps of SOM clusters of the K20 set. (.pdf format);

Supplementary data VI - Unified list of highly enriched k-mers per DTU. (.xls format);

Supplementary data VII - SOM clusters association with DTUs (.xls format);

Supplementary data VIII - Strain-enriched k-mers (.xls format).

For the Supplementary data III, rank is the k-mer position in decreasing order for the distribution of the average of normalised read count for all samples. Start is the left most position of the k-mer in the specific trans-sialidase prototype sequence used for the BLAST search. %ident is the percentage identity of the k-mer to the trans-sialidase prototype sequence. HSP is the high scoring sequence pair size, i.e., the size of the local BLAST alignment between the k-mer and the trans-sialidase prototype sequence. Normally it will have 20 nt, as the complete k-mer was found in the sequence, without gaps (but probably mismatches); if the HSP is smaller than 20 it generally means that the similarity between the k-mer and the trans-sialidase sequence is not total, in the k-mer ends; if the HSP is larger than 20 it generally means that there were gaps between the k-mer and the trans-sialidase target. Obviously, mismatches and gaps can occur concurrently, as well as insertions and deletions, which will have an impact in HSP size and % ident.

For the Supplementary data IV-**V**, the stocks depicted in each column are in the same order as in the Supplementary data III. The colour gradient is a relative scale (z-score) of the k-mer count where the lowest valor is black and gradually increasing frequencies are coloured as increasing shades of yellow.
