# Supplementary material for: A new Trypanosoma cruzi genotyping method enables high resolution evolutionary analyses
Source: Mem Inst Oswaldo Cruz. 2021 Aug 30;116:e200538. doi: 10.1590/0074-02760200538 (PMC8405150; doi:10.1590/0074-02760200538)

TABLE I

Reads generated for the prototyping assay with quality parameters

Sample	# bases	# >Q20 bases	# reads	Mean size (bp)	#reads post-filtering	% reads filtered
DTU TcIa	3,058,539	2,621,866	20,549	149	11,060	53.8%
DTU TcIb	3,149,896	2,661,231	21,715	145	10,785	49.7%
DTU TcIIa	3,395,864	2,891,783	23,160	147	11,710	50.6%
DTU TcIIb	2,817,143	2,395,160	18,624	151	9,562	51.3%
DTU TcIIIa	2,829,480	2,407,999	19,563	145	9,377	47.9%
DTU TcIIIb	2,451,821	2,046,330	17,111	143	7,965	46.5%
DTU TcIVa	2,990,534	2,542,199	20,995	142	10,850	51.7%
DTU TcIVb	1,970,502	1,594,421	14,269	138	6,404	44.9%
DTU TcVa	2,850,324	2,631,362	20,212	145	9,967	49.3%
DTU TcVb	2,651,921	2,331,191	19,824	140	9,796	49.4%
DTU TcVIa	2,879,056	2,461,879	19,509	148	10,186	52.2%
DTU TcVIb	2,727,667	2,259,801	19,659	139	8,967	45.6%

Q20: Phred-like quality score, meaning an error probability per base of <1% (>Q20 score)

Filtering: A BLASTn comparison was performed against the target sequence prototype and only those reads that had a complete sequence (start and end of the amplicon, excluding primer sites) were selected.

Stocks and Replicates - DTU TcI: Dm28c, TcII: Y, TcIII: 3663, TcIV: 4167, TcV: Bug2148, TcVI: CL Brener. A and B - technical replicates

CC BY

TABLE II

Number of clusters generated for the prototyping assay using distinct thresholds

Sample	# clusters >99%	# clusters >98%	# clusters >97%	# clusters >95%	# clusters >90%	# clusters >80%
DTU Ia	4,075	2,032	1,245	489	96	95
DTU Ib	4,534	2,345	1,336	489	66	66
DTU IIa	4,124	2,020	1,176	522	132	124
DTU IIb	3,699	1,838	1,081	661	71	63
DTU IIIa	4,404	2,445	1,401	829	32	27
DTU IIIb	3,667	1,959	1,179	721	24	21
DTU IVa	4,451	2,188	1,236	747	87	84
DTU IVb	3,341	1,885	1,212	755	39	30
DTU VIa	4,163	2,285	1,383	888	91	82
DTU VIb	3,964	2,041	1,249	814	61	44

The reads with complete sequence (excluding primer annealing region) were compared all-against-all using the BLASTn software and clustered with the MCL algorithm. The resulting BLAST similarity results were filtered with different similarity thresholds (80%, 90%, 95%, 97%, 98% and 99%) before submitting to the clustering procedure. With higher similarity thresholds, the sequencing error has a significant impact in the number of clusters; with a threshold of 80%, we are close to a lower boundary of how many distinct trans sialidase sub-families amplified by our primers we have for each DTU strain.

The higher similarity level thresholds (97%, 95%) give us an idea if sequencing errors are still significantly influencing the final results. We can see that, in general, the number of clusters are generally similar between DTUs and between technical replicates. This suggests that the technical error associated with the PCR amplification, sample preparation for sequencing and the sequencing process itself had little impact at this level of representation.

The second technical replicate (B) generally has a lower number of clusters for the lower similarity levels (90%, 80%). This probably is caused by the lower number of reads generated for these samples; besides, the missing clusters are composed of low frequency reads (data not shown), what suggests that they are sequencing errors and/or low frequency informative sequences.

The DTU TcV samples were excluded from the analysis, as they were identified as DTU TcI.

Figure 1. Comparison of k-mer frequency by DTU from the prototyping assay.

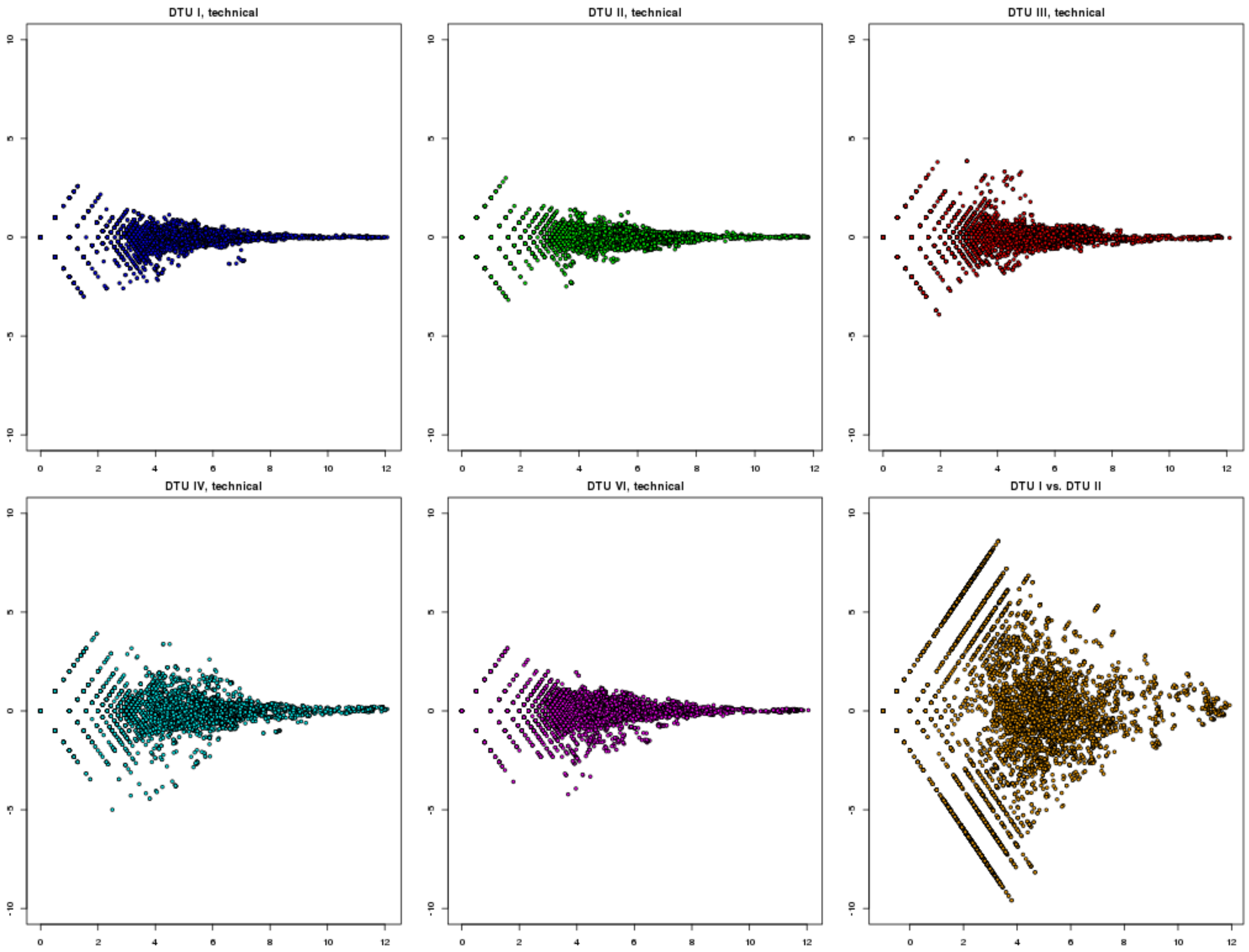

X-axis: $\log_2$ of normalised mean read count; Y-axis: $\log_2$ of the ratio between normalised read counts between samples.

Figure 2. Neighbor-joining JSD distance tree of technical replicates of DTU samples.

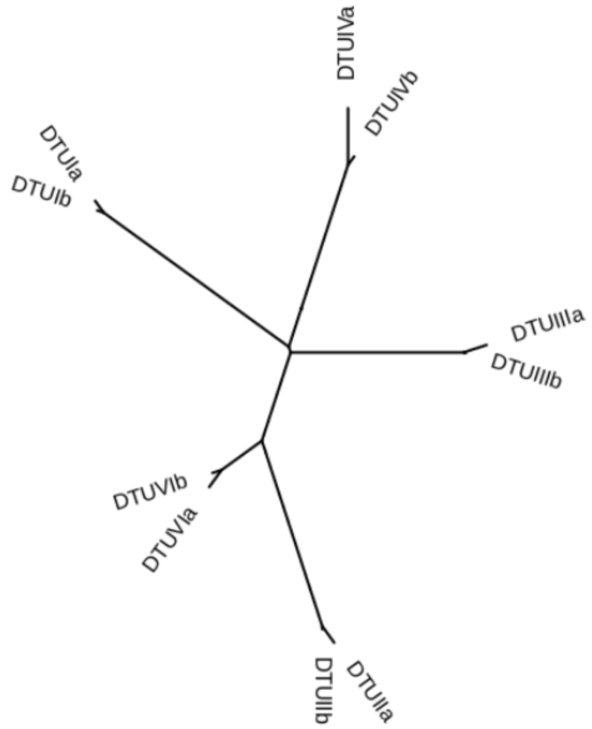

Mem Inst Oswaldo Cruz, Rio de Janeiro, Vol. *116*: e200538, 2021

TABLE III

Reads generated for the phylogenetic assay

Sample	Sequencing run	DTU	# reads	#reads passed	% reads passed
12SF	A	II	11,363	7,141	62.8%
222	A	III	47,300	23,039	48.7%
3663	A	III	24,936	15,309	61.4%
4167	A	IV	18,501	11,321	61.2%
AM14R3	A	III	53,460	29,703	55.6%
CanIII	A	IV	18,600	8,113	43.6%
CL Brener A	A	VI	23,934	14,411	60.2%
CL Brener B	B	VI	16,785	12,881	76.7%
Colombiana	A	I	8,987	5,447	60.6%
D8	A	I	20,301	12,318	60.7%
Dm28c	A	I	18,102	8,735	48.3%
G	A	I	120,619	43,174	35.8%
LL014	A	V	17,460	8,266	47.3%
Peruana	B	VI	30,868	23,217	75.2%
SO3 cl5	A	V	37,527	22,086	58.9%
TcBat	B	TcBat	31,210	5,581	17.9%
T. c. marinkellei	B	*T.c. marinkellei*	44,513	32,236	74.7%
Tulahuen cl. 0	A	I	37,379	21,605	57.8%
CP300	B	VI	31,787	23,431	73.7%

TABLE IV

Distinct k-mers identified according to distinct thresholds of sampling

	1	2	3	4	5	10	20	29
1	1,049,005	372,221	236,334	172,945	135,919	62,629	22,205	4,400
5	120,280	74,393	60,659	50,084	42,280	22,812	8,987	1,774
10	84,857	54,954	45,475	37,487	31,782	16,825	6,590	1,197
20	62,908	39,603	32,328	26,624	22,432	11,797	4,353	844
50	37,952	21,439	16,844	13,732	11,632	5,826	2,401	646
100	22,641	11,659	8,726	7,093	5,978	3,213	1,340	531

At least X times (rows) a k-mer was identified in Y samples (columns). For instance, 4,400 k-mers were identified at least one time in all samples (very rare, conserved k-mers); 22,641 were identified only in one sample but with read count higher than 100 (sample specific k-mers, that are enriched); 531 were identified in all samples with very high read count (very conserved, very ubiquitous k-mers).

TABLE V

Informative k-mers considering distinct levels of fold difference between samples and k-mer counting threshold

	2	5	10	20	50	100	200	500
Total (>=1)	119,965	105,803	83,687	58,976	33,276	18,632	8,433	0
Count >=2	102,022	94,533	81,439	58,976	33,276	18,632	8,433	0
Count >=5	70,797	70,512	64,656	54,757	33,276	18,632	8,433	0
Count >=20	30,404	30,135	29,698	29,256	23,524	17,067	8,433	0
Count >=100	5,729	5,487	5,198	4,979	4,614	4,320	3,215	0

A k-mer was observed at least X times (rows) and the k-mer count ratio between the samples with the highest and lowest counts are at least more than Y fold (columns).

TABLE VI

Discriminative k-mers for the phylogenetic entities analysed in this paper

Fold	DTU I	DTU II	DTU III	DTU IV	DTU V	DTU VI	TcBat	*T. c. marinkellei*
>=2	973	420	173	1364	373	1098	928	735
>=5	508	23	32	686	91	463	309	249
>=10	186	3	7	324	26	43	52	105
>=20	60	0	0	134	15	0	13	32
>=50	0	0	0	0	4	0	0	18

A k-mer was observed X-fold (rows) increased in the specific DTU when compared to all other DTUs. In other words, it was the ratio between the k-mer count of the sample pertaining to the selected DTU with the lowest count and that from all other samples with the highest count.

Mem Inst Oswaldo Cruz, Rio de Janeiro, Vol. *116*: e200538, 2021

TABLE VII

Discriminative k-mers when comparing all samples analysed

Fold	>=5	>=10	>=20	>=50	>=100	>=200	>=300	>=400
# k-mers	35,886	28,009	17,996	6,217	1,697	217	25	1

A k-mer was observed at least X-fold (columns) between the sample with the highest and the lowest k-mer count. In order to attenuate sampling bias for rarer k-mers and to avoid division by zero, a weight of one count was added for each k-mer.

Figure 3. Electrophoretic analysis of the target amplicon.

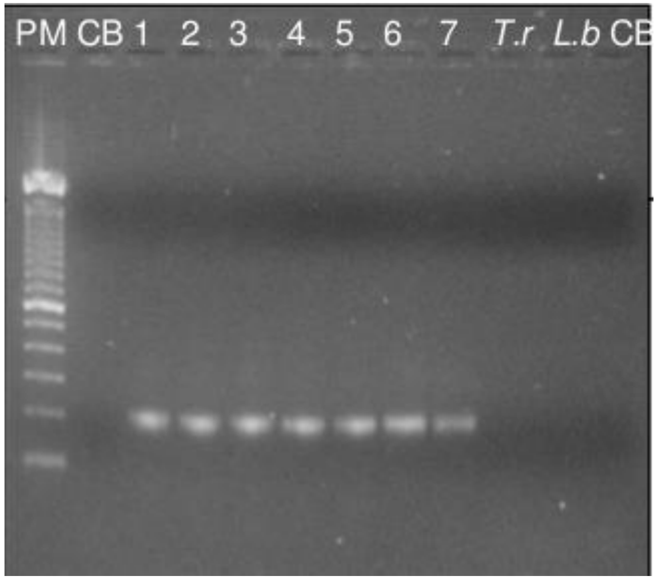

PM - Molecular weight, CB - blank control, 1- Colombiana strain, 2- Dm28c clone, 3- Y strain, 4- 3663 strain, 5- 4167 strain, 6-LL014 strain, 7- CL Brener strain, T.r - *Trypanosoma rangeli*, *L.b. - Leishmania braziliensis*.

Figure 4. Location of DTU enriched k-mers.

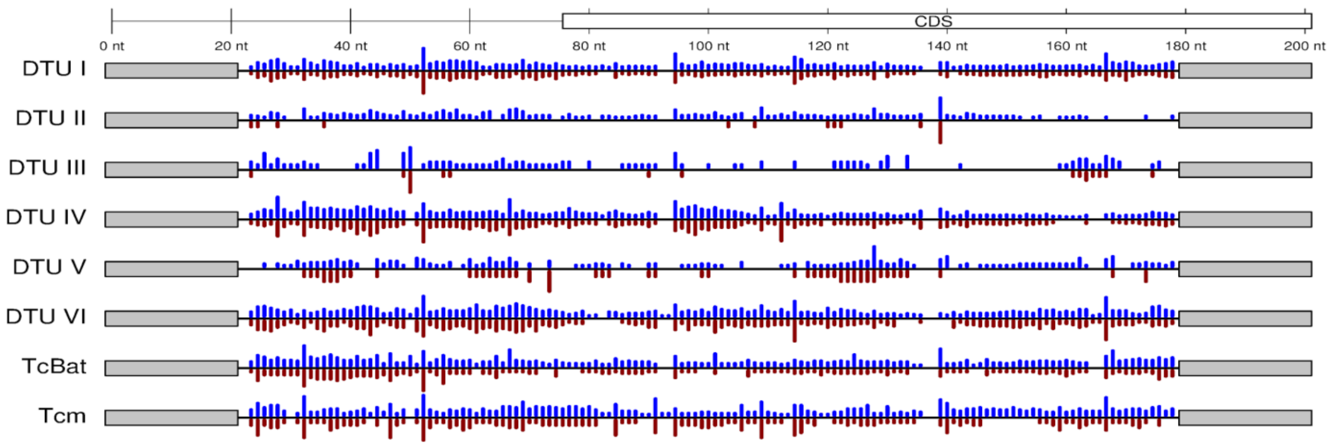

The complete amplicon is shown. Grey boxes represent the PCR primers; the location of the CDS is shown as a white box. Blue bars are k-mers whose fold-enrichment is higher than 20; red bars are k-mers whose fold-enrichment is higher than 100. Relative k-mer frequency is the bar height.

Figure 5. Phylogenetic tree based on the euclidean distance of the K100 set.

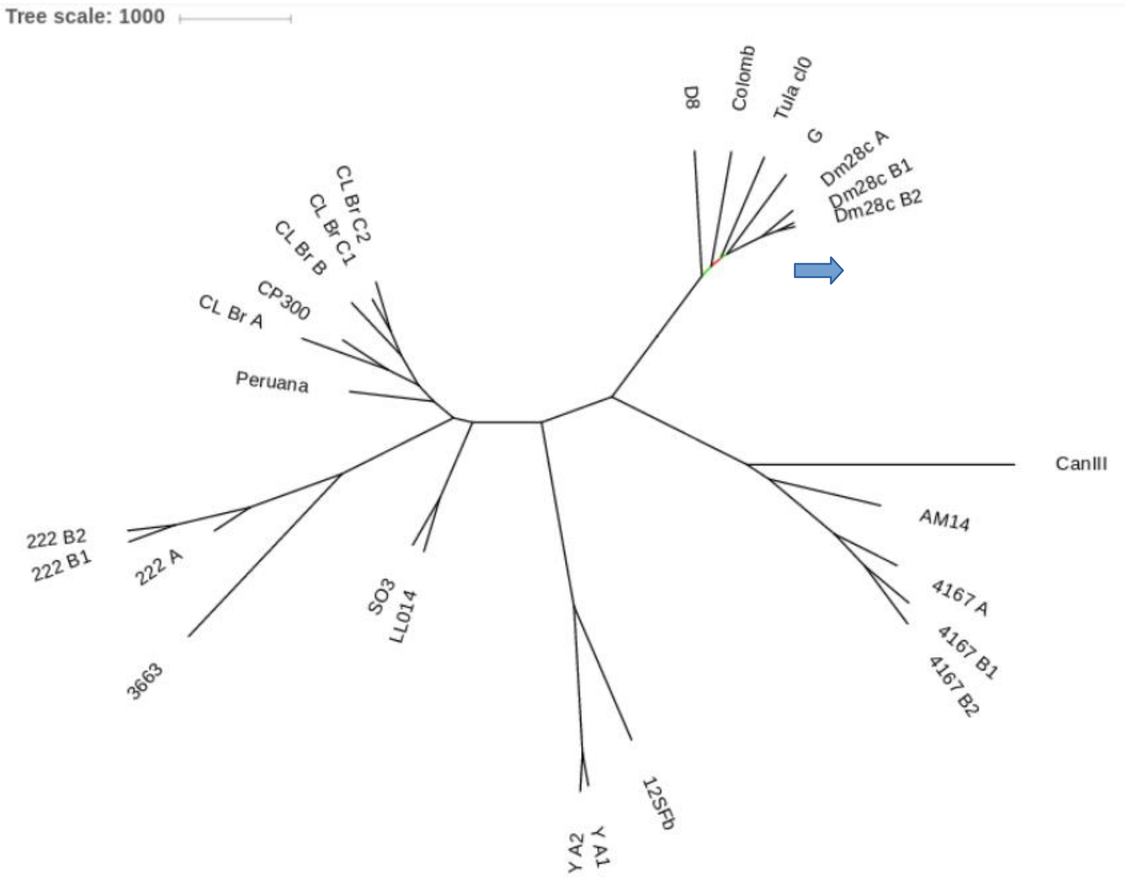

All branches showed 100% bootstrap support, with the exception of the branches depicted in green (84.3% and 86.2%) and red (49.7%), illustrating that the position of D8, Colombiana and Tulahuen cl0 are not well resolved inside the DTU I (blue arrow).

Figure 6. Phylogenetic tree based on the jaccard distance of the K100 set.

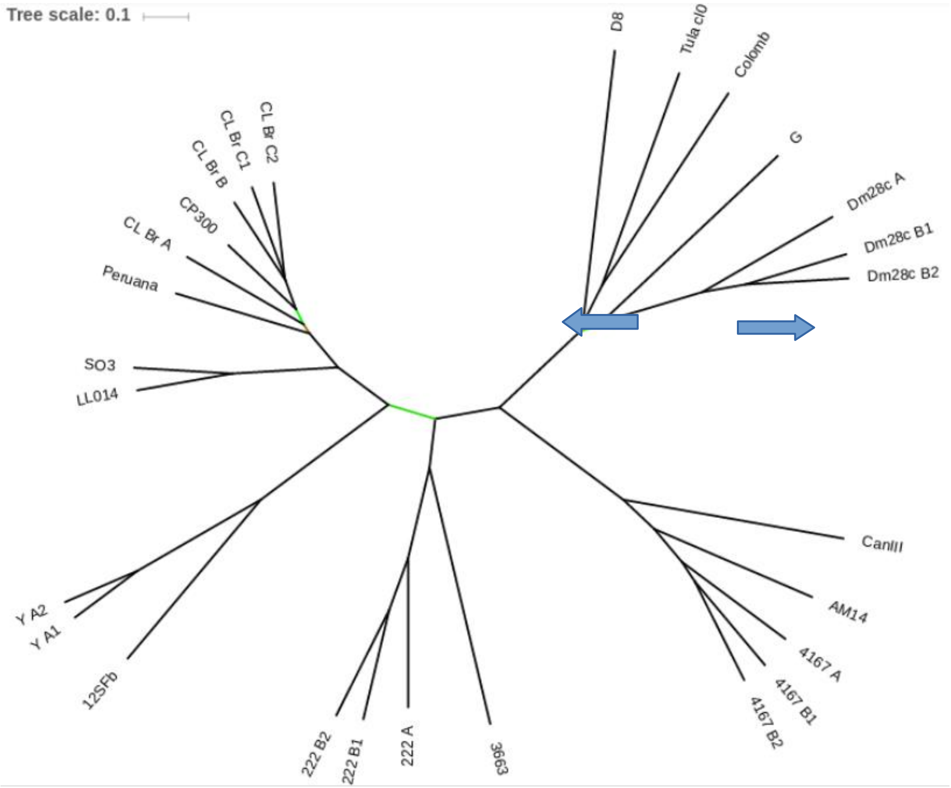

All branches showed 100% bootstrap support, with the exception of the branches depicted in green (from 85.6% to 89.6%) and orange (69.6% and 71.1%, blue arrows).

Figure 7. Phylogenetic tree based on the euclidean distance of the K20 set.

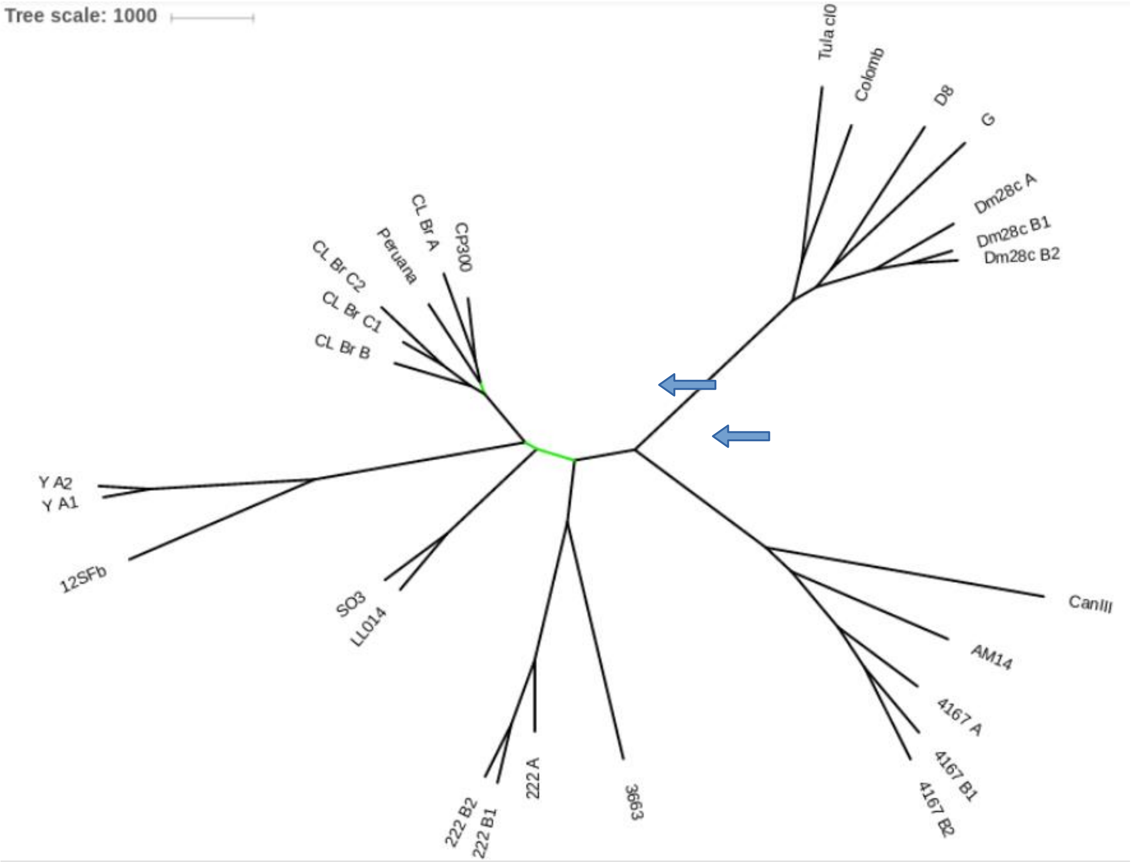

All branches showed 100% bootstrap support, with the exception of the branches depicted in green (from 86.2% to 86.7%, blue arrows).

Figure 8. Phylogenetic tree based on the jaccard distance of the K20 set.

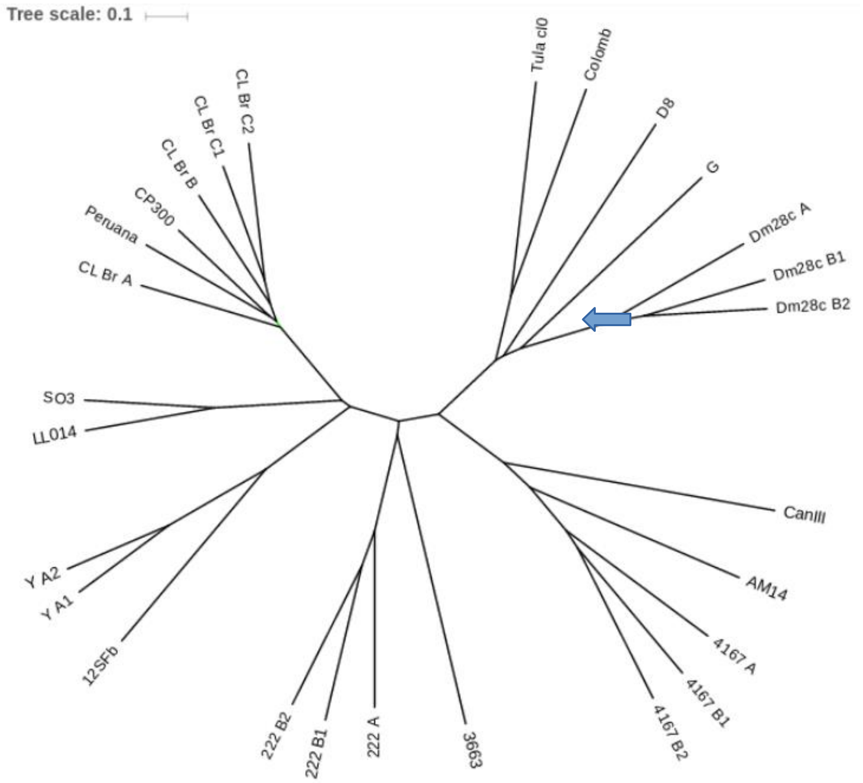

All branches showed 100% bootstrap support, with the exception of the branch depicted in green (87.6%, blue arrow).

Figure 9. Phylogenetic tree based on the euclidean distance of the 1,000 most common k-mers

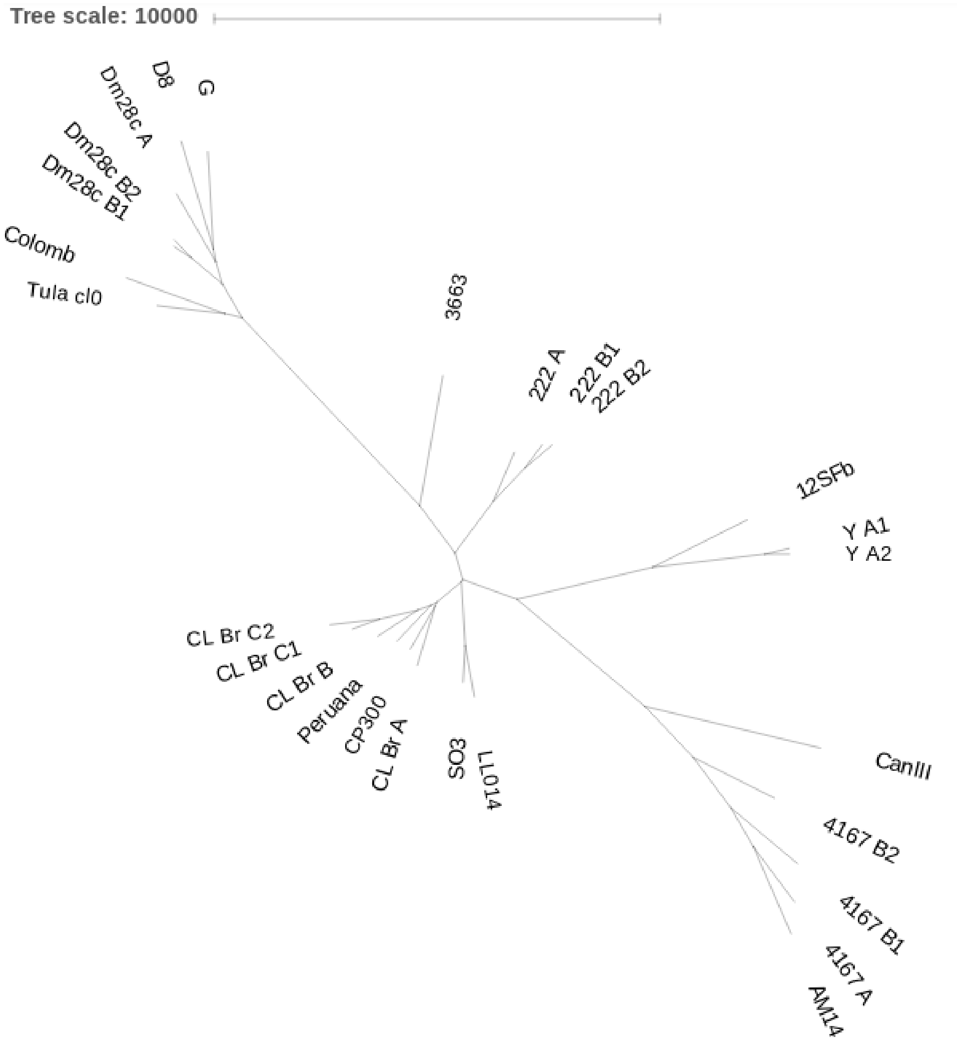

Figure 10. Phylogenetic tree based on the euclidean distance of the 2,000 most common k-mers.

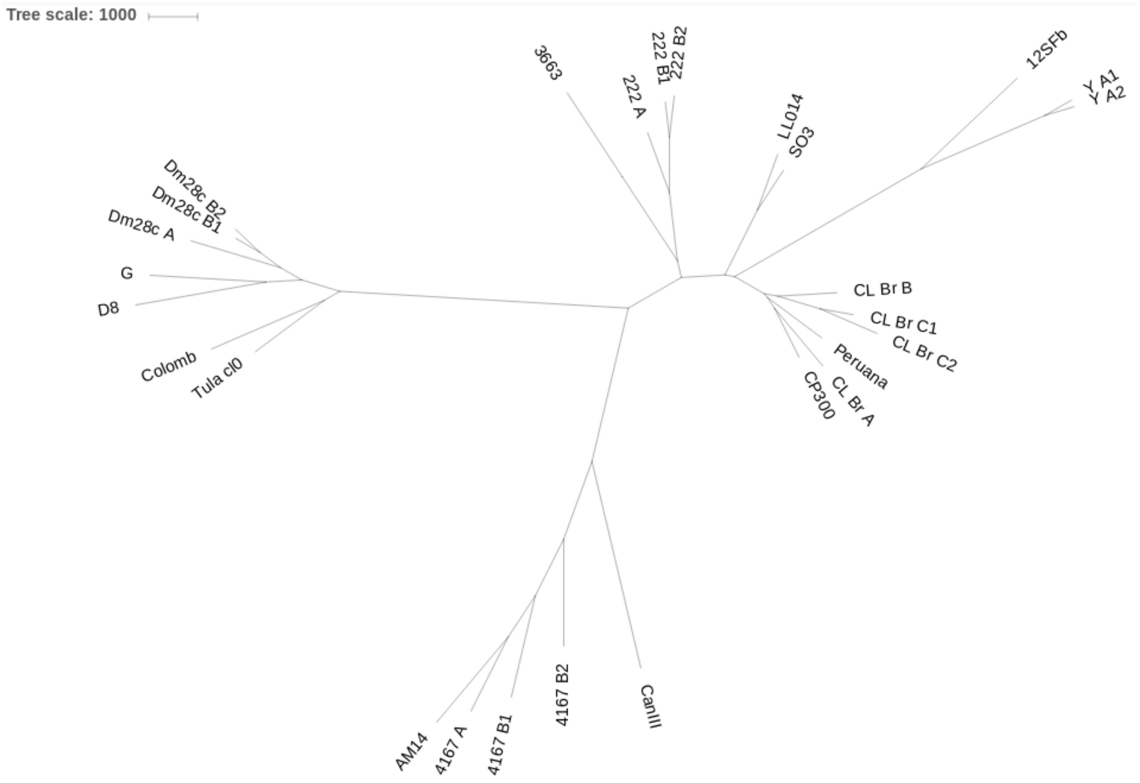

Figure 11. Phylogenetic tree based on the euclidean distance of the K20 set including TcBat and *T. c. marinkelei* samples.

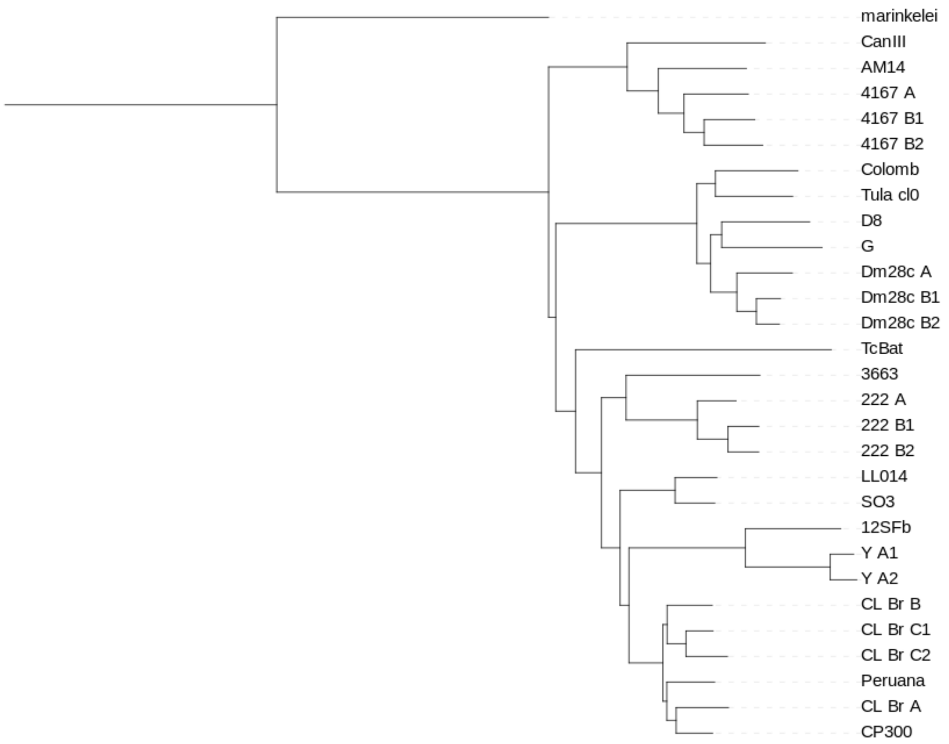

TABLE VIII

T. cruzi genomes available at NCBI (March 2021), including the number of trans-sialidase targets identified by the *in silico* analysis using primer sequences and BLASTn search

Strain	Code	DTU	# TS targets	Technology	Strain	Code	DTU	# TS targets	Technology
Arequipa	PYLF01	TcI	255	454	S162a	PYLC01	TcII	220	Illumina HiSeq
B.M.Lopez	WWPY01	TcI	12	Ion Torrent	S23b	PYLB01	TcII	172	Illumina HiSeq
Brazil	WNWZ01	TcI	605	PacBio	S44a	PYLA01	TcII	71	Illumina HiSeq
Bug2148	NMZN01	TcI	787	PacBio	S92a	PYKZ01	TcII	142	Illumina HiSeq
Colombiana	PYLG01	TcI	273	454	Y A	WYWN01	TcII	576	PacBio
Dm28c A	MBSYS01	TcI	703	PacBio	Y B	NMZO01	TcII	245	Illumina MiSeq
Dm28c B	PRFA01	TcI	714	PacBio	Y C	PYLH01	TcII	330	454
Dm28c C	AYLP01	TcI	351	454	Y cl2	PYKY01	TcII	166	Illumina HiSeq
G	MKKV01	TcI	122	454	Y cl4	PYKX01	TcII	155	Illumina HiSeq
JR cl. 4	AODP01	TcI	209	454	Y cl6	PYKW01	TcII	126	Illumina HiSeq
Sylvio X10/1	ADWP02	TcI	227	454 + Illumina HiSeq	231	OGCJ01	TcIII	282	Illumina HiSeq
Berenice	JABDHM01	TcII	487	Oxford	Ikiakora	WWPZ01	TcIII	0	Ion Torrent
Esmeraldo cl3	ANOX01	TcII	255	454	Sc43	JACCJE01	TcV	837	Illumina NextSeq
S11	PYKV01	TcII	144	Illumina HiSeq	CL	MKQG01	TcVI	388	454
S15	PYLE01	TcII	169	Illumina HiSeq	TCC	PRFC01	TcVI	840	PacBio
S154a	PYLD01	TcII	26	Illumina HiSeq	Tula cl 2	AQHO01	TcVI	142	454

Figure 12. Overview of the steps involved in the TS-LSS method.

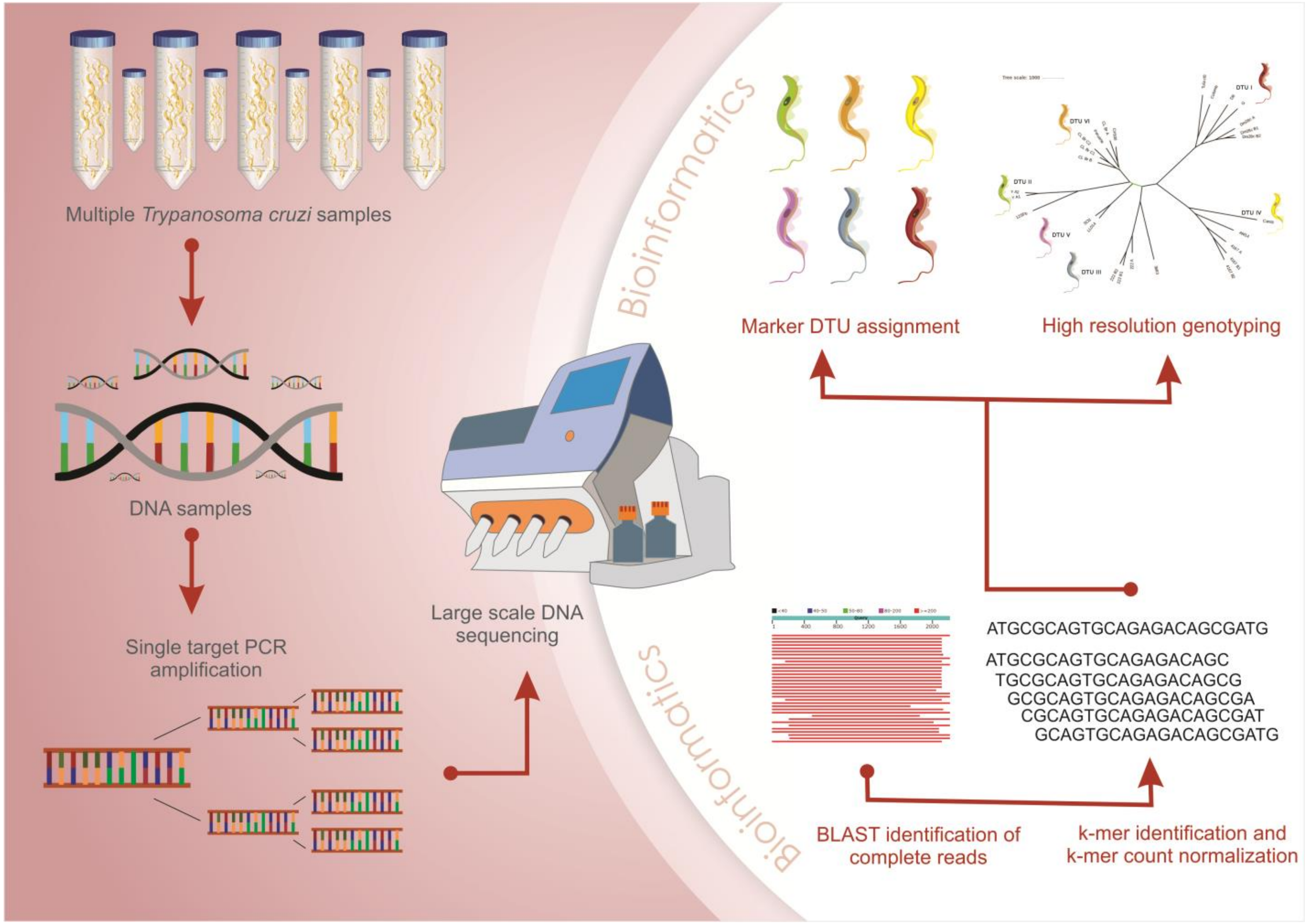

Supplement: Supplementary file 1 [file 1678-8060-mioc-116-e200538-s1.pdf]
