# Supplementary material for: A new Trypanosoma cruzi genotyping method enables high resolution evolutionary analyses
Source: Mem Inst Oswaldo Cruz. 2021 Aug 30;116:e200538. doi: 10.1590/0074-02760200538 (PMC8405150; doi:10.1590/0074-02760200538)

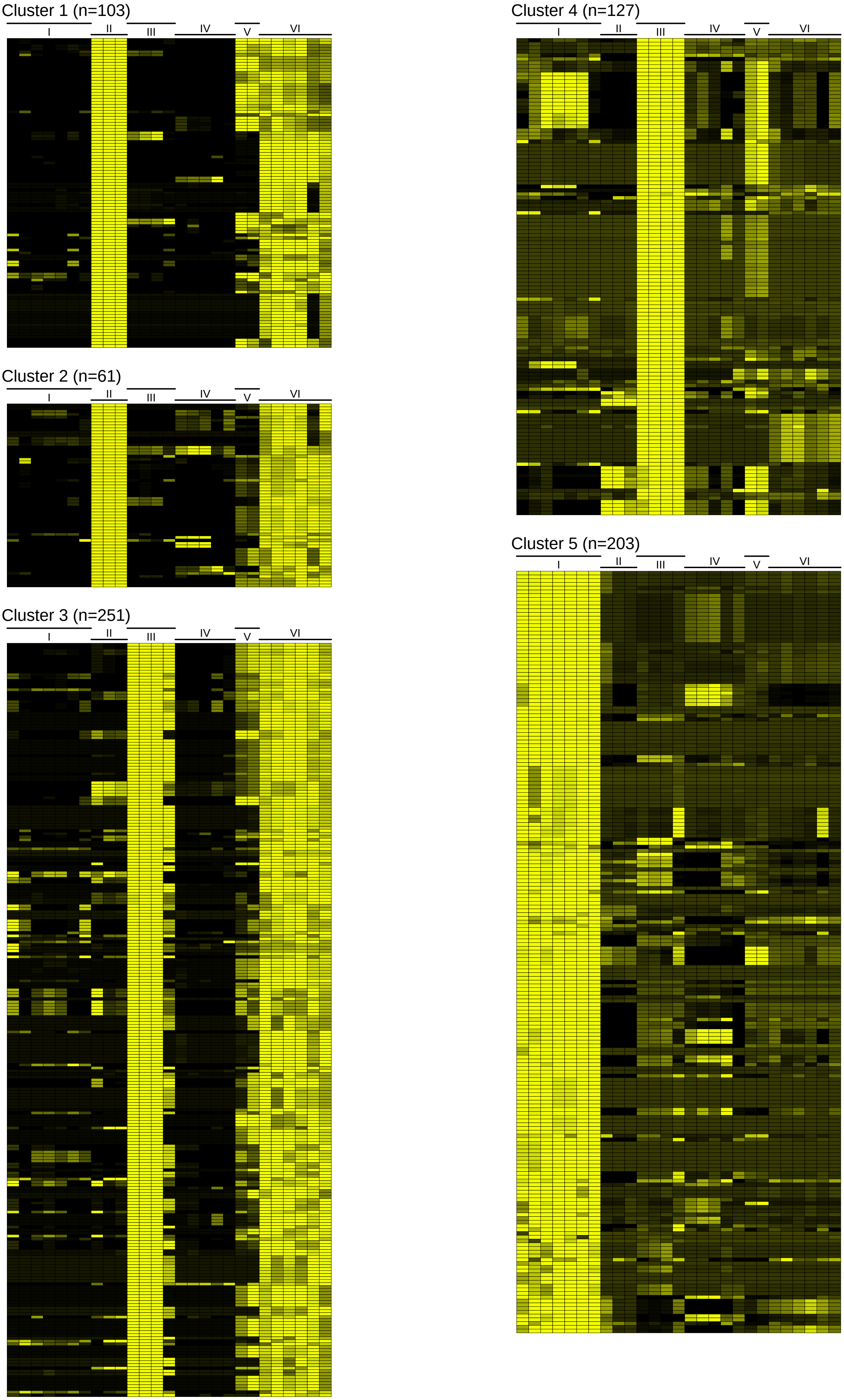

Cluster 1 (n=103)
I
II
III
IV
V
VI
Cluster 2 (n=61)
I
II
III
IV
V
VI
Cluster 3 (n=251)
I
II
III
IV
V
VI
Cluster 4 (n=127)
I
II
III
IV
V
VI
Cluster 5 (n=203)
I
II
III
IV
V
VI

Cluster 6 (n=219)

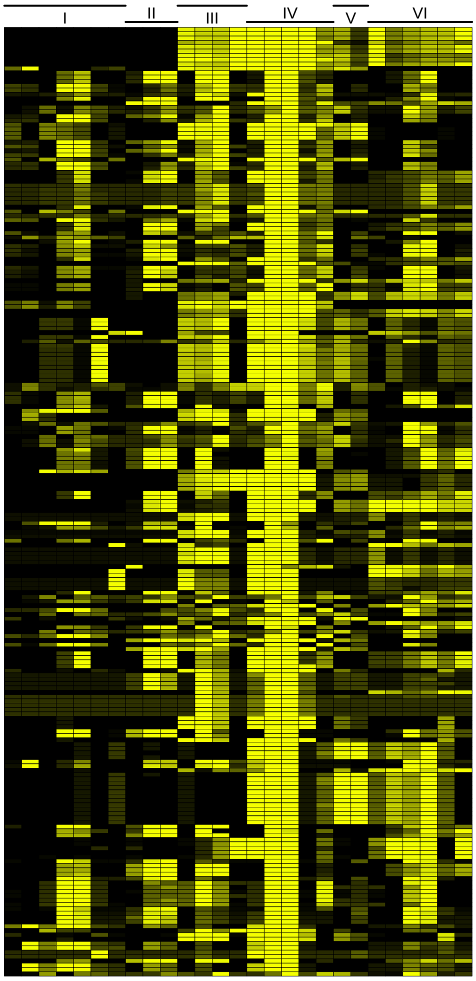

Cluster 7 (n=198)

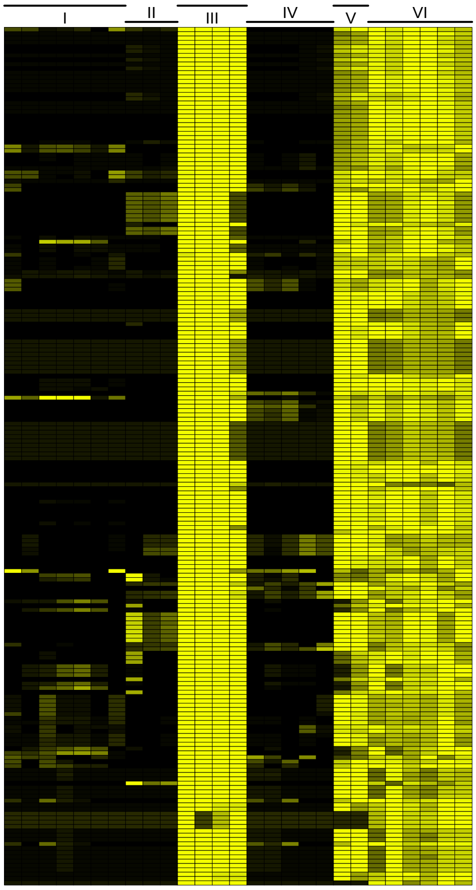

Cluster 8 (n=187)

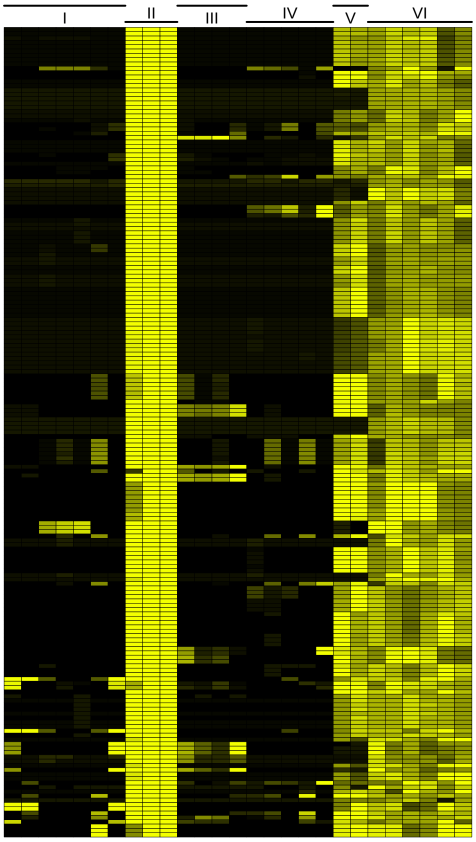

Cluster 9 (n=257)

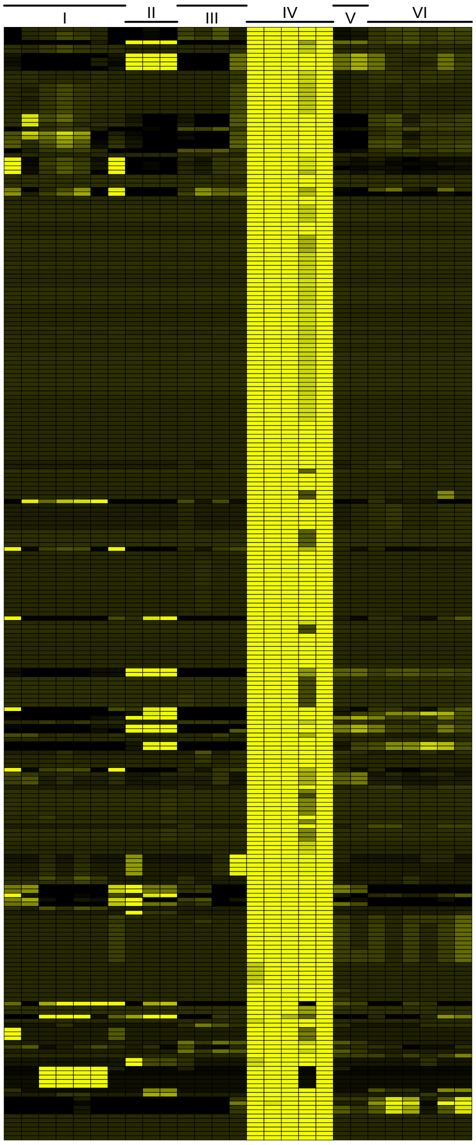

Cluster 10 (n=177)

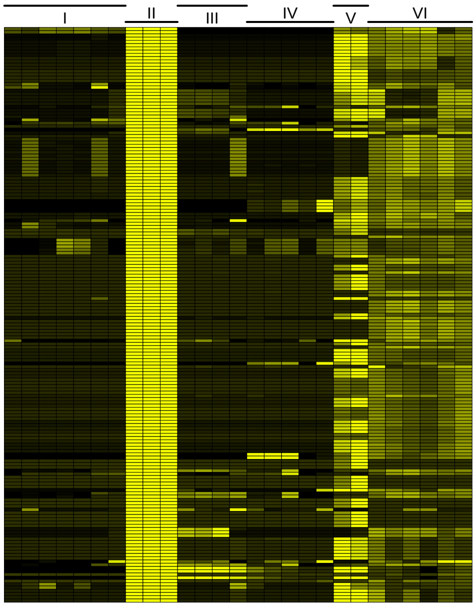

Cluster 11 (n=325)

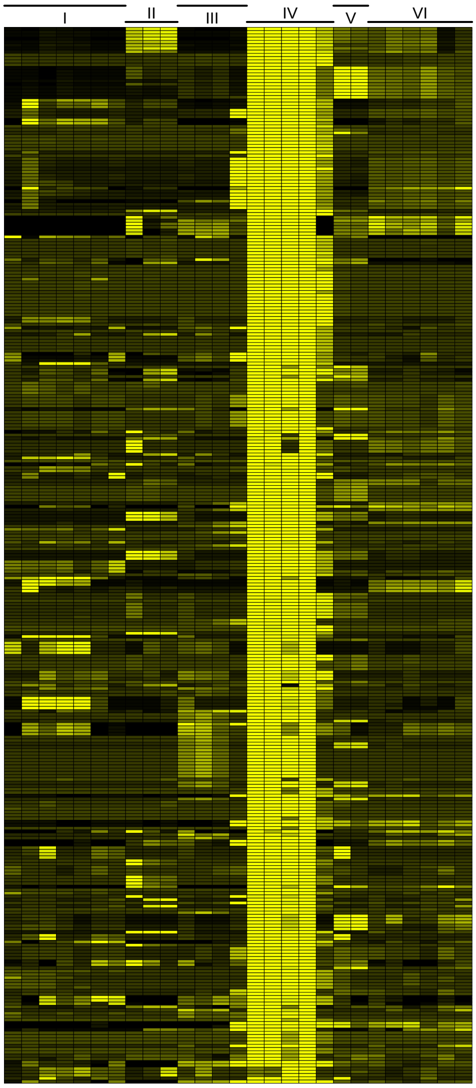

Cluster 12 (n=155)

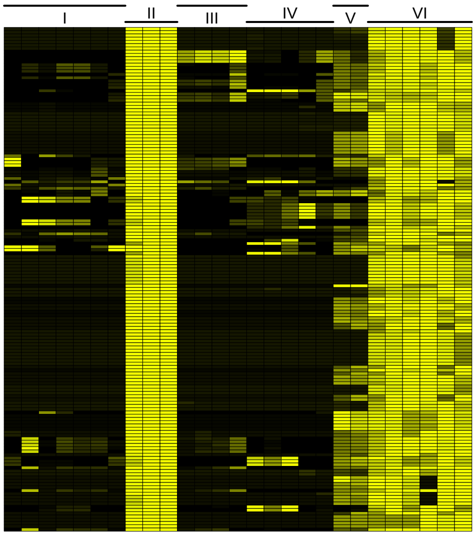

Cluster 13 (n=328)

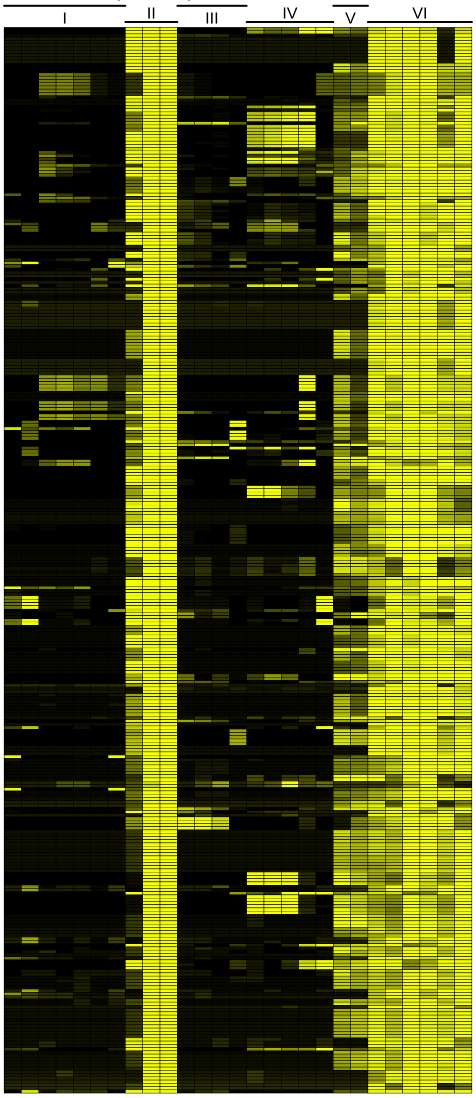

Cluster 14 (n=131)

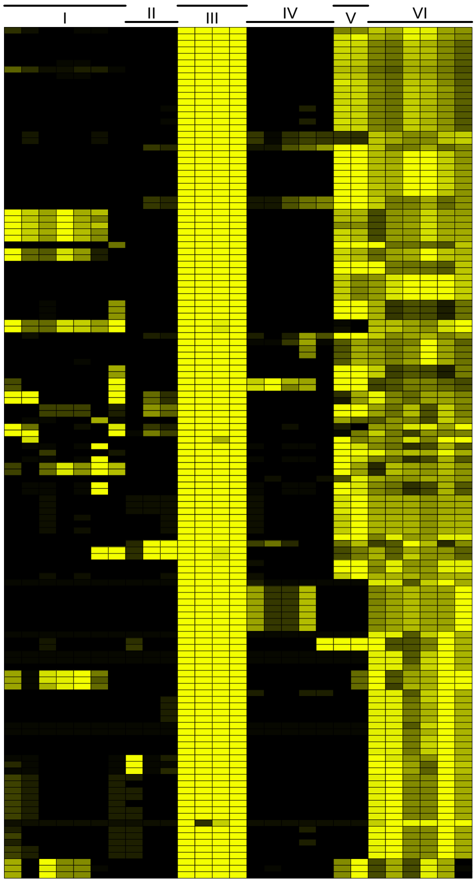

Cluster 15 (n=143)

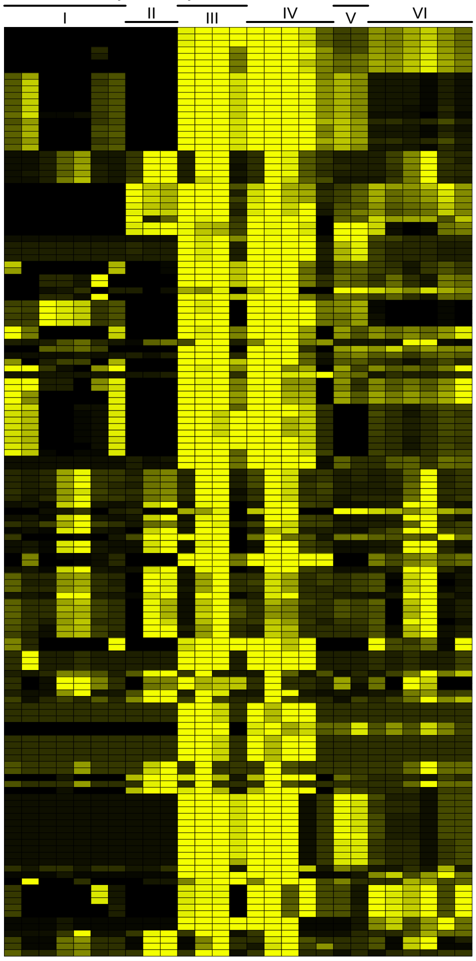

Cluster 16 (n=93)

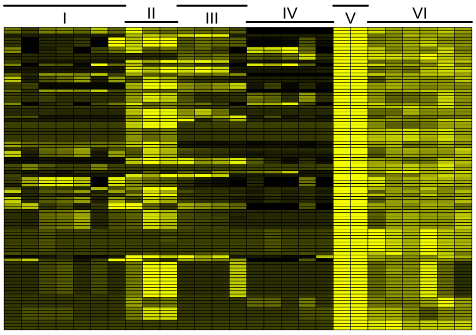

Cluster 17 (n=421)

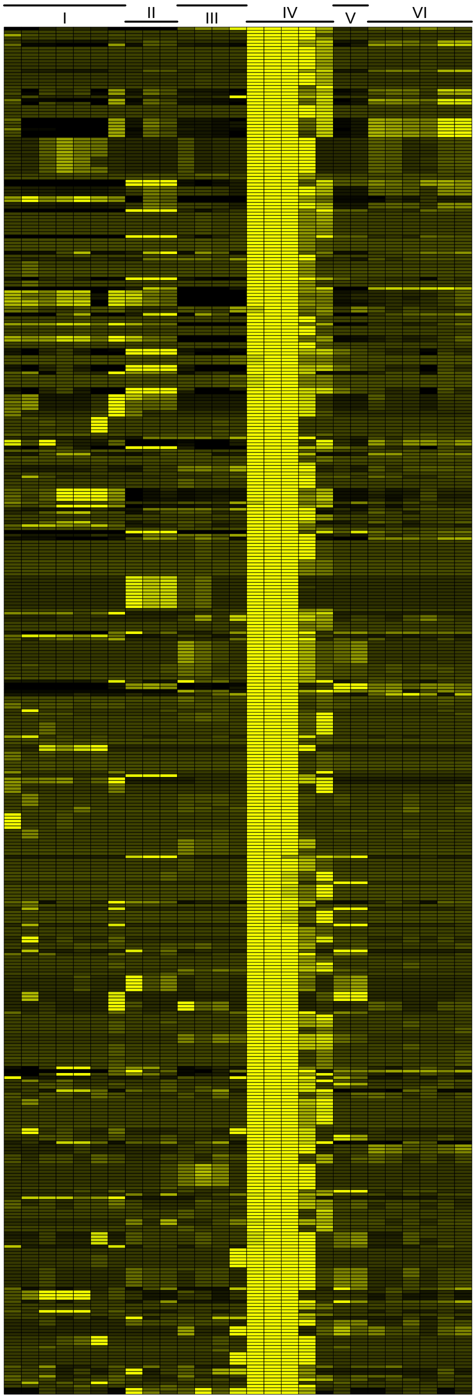

Cluster 18 (n=123)

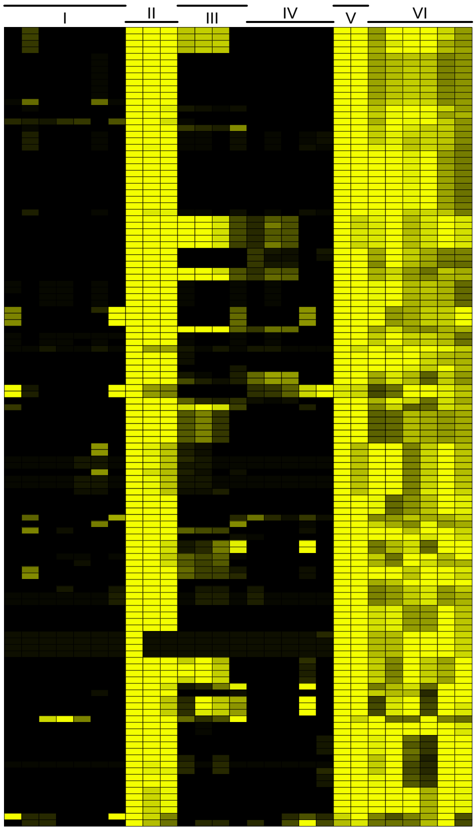

Cluster 19 (n=179)

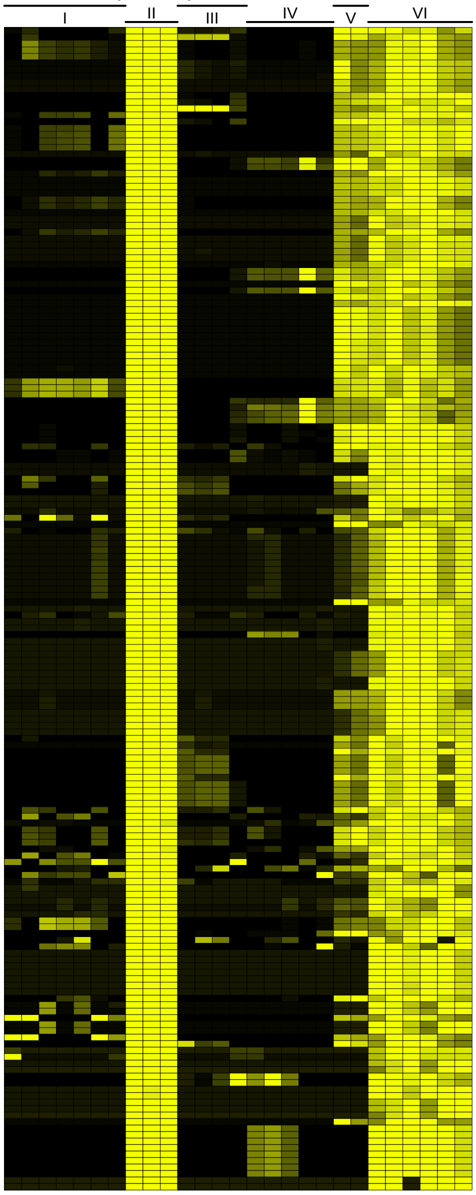

Cluster 20 (n=119)

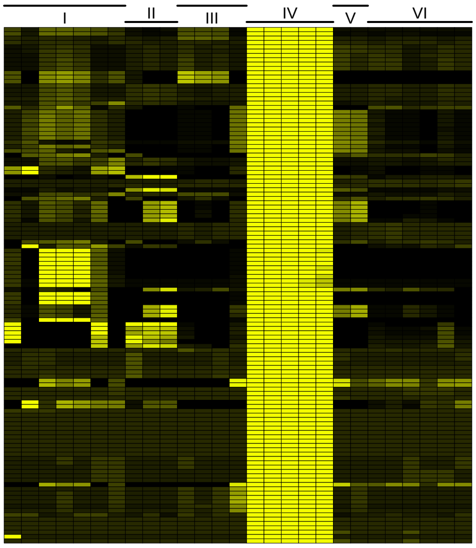

Cluster 21 (n=326)

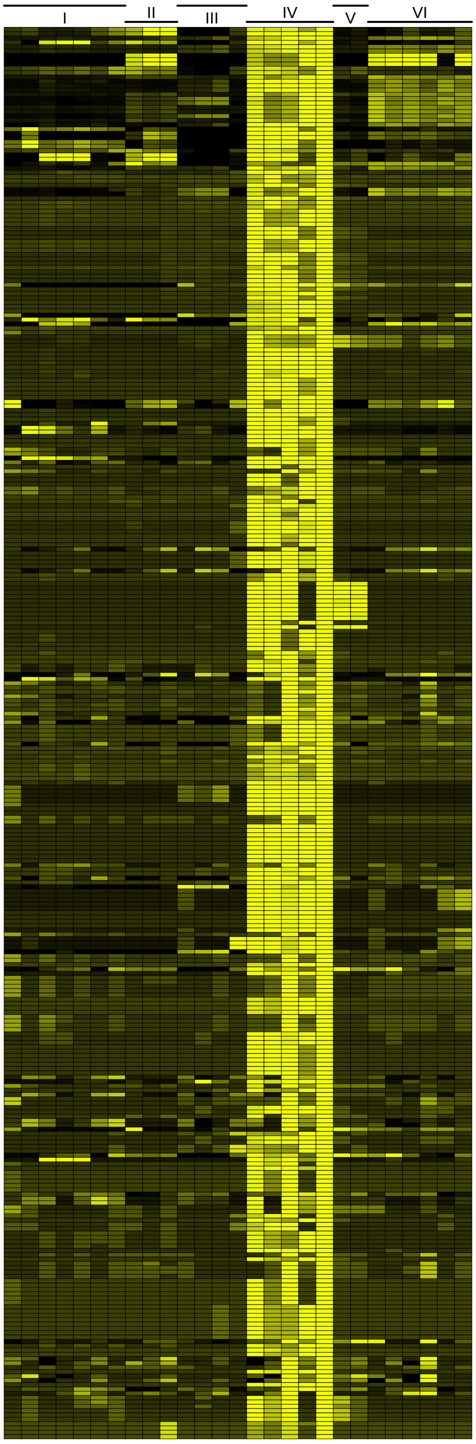

Cluster 22 (n=88)

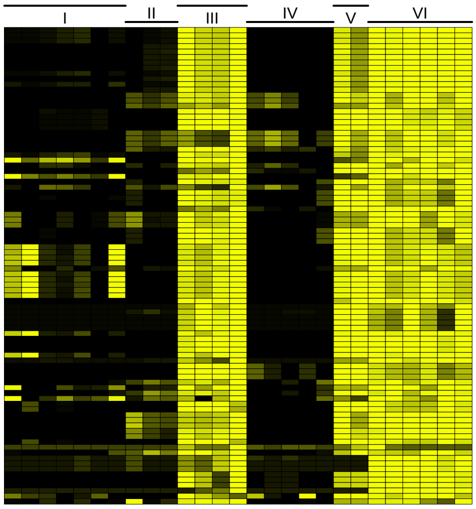

Cluster 23 (n=224)

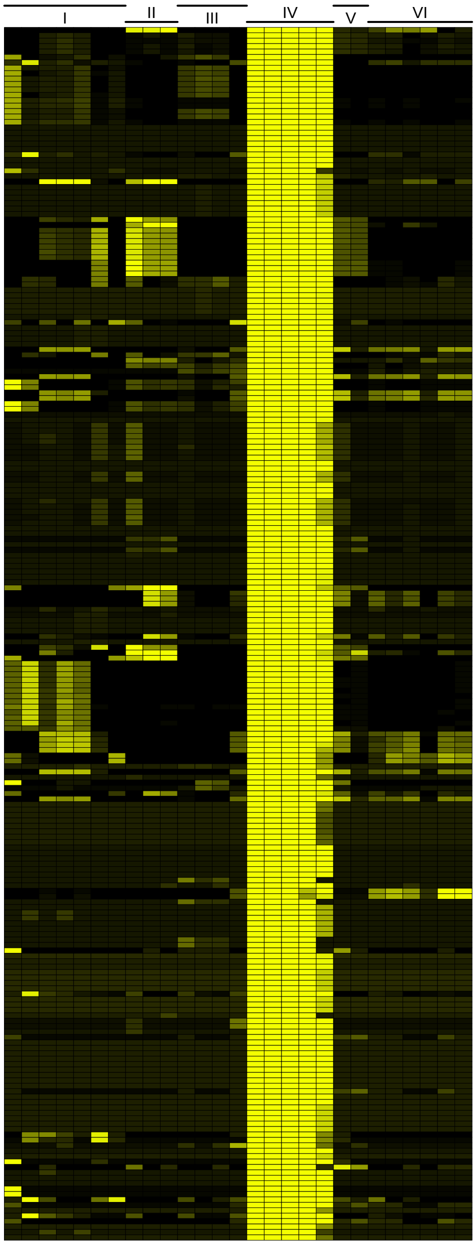

Cluster 24 (n=29)

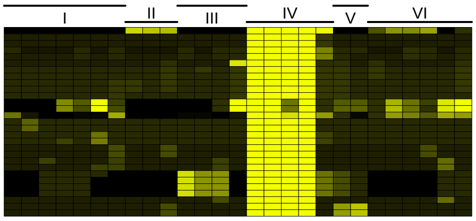

Cluster 25 (n=216)

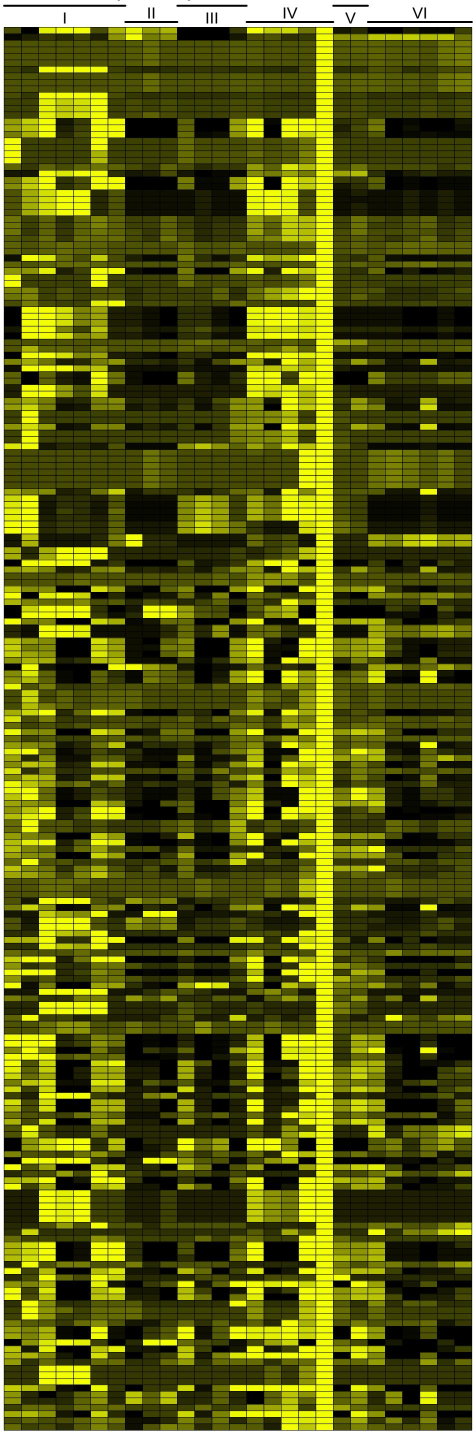

Cluster 26 (n=128)

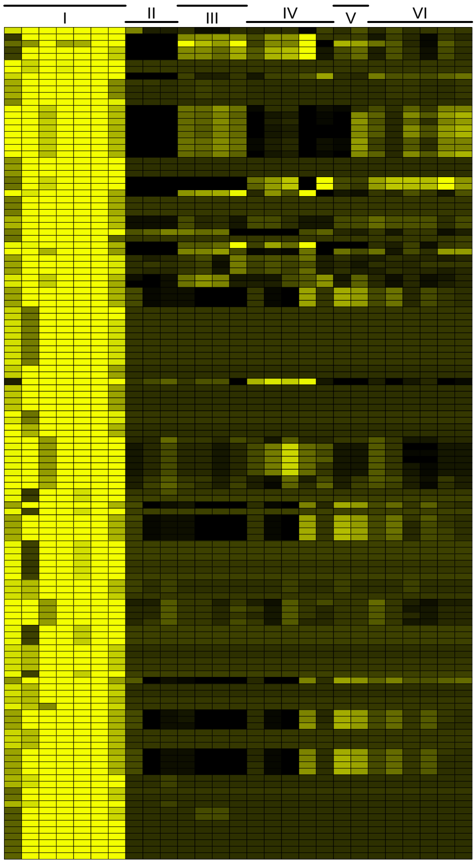

Cluster 27 (n=130)

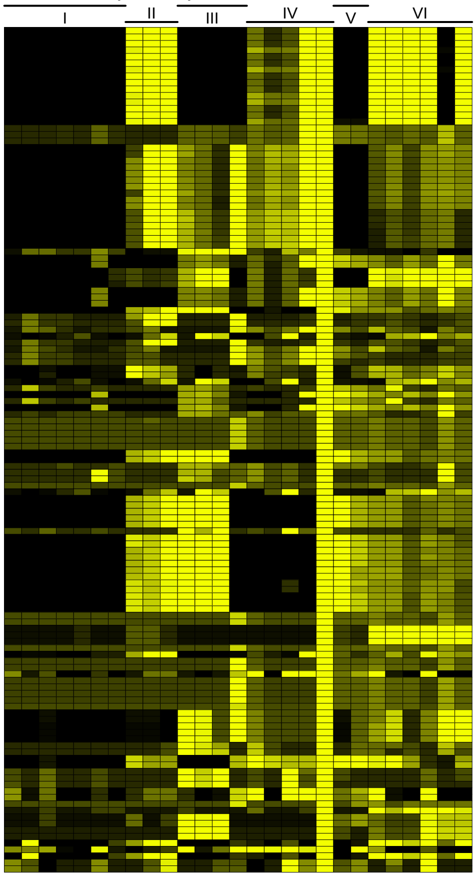

Cluster 28 (n=184)

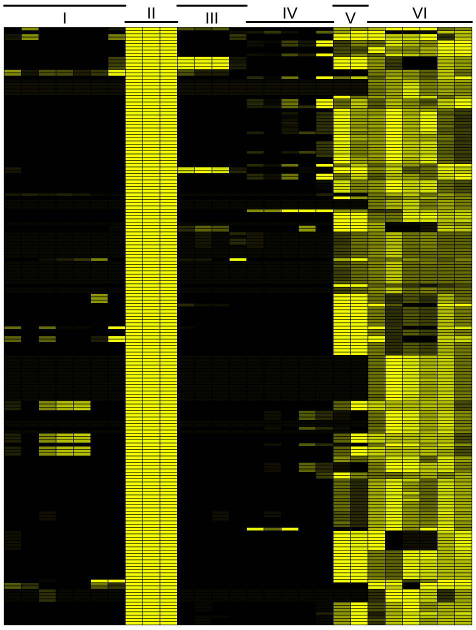

Cluster 29 (n=345)

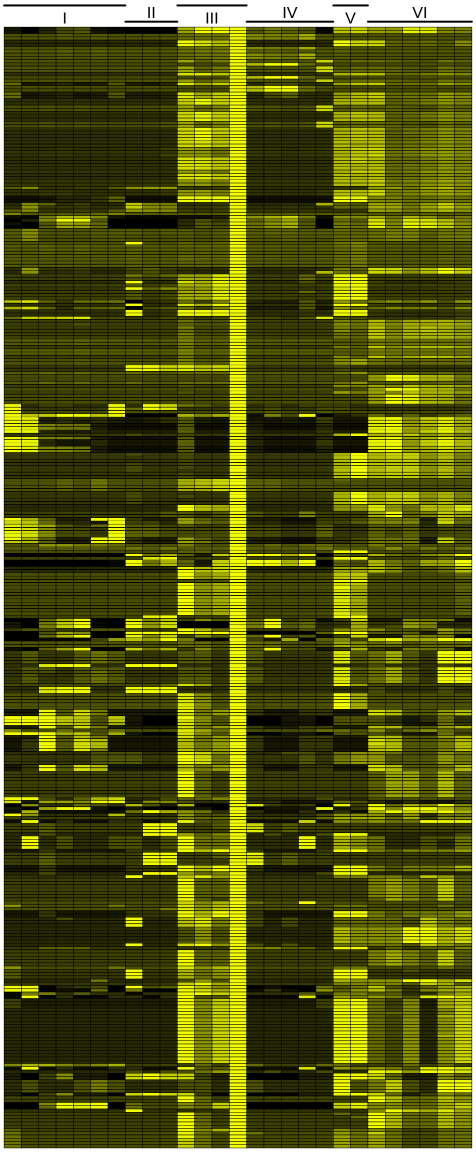

Cluster 30 (n=80)

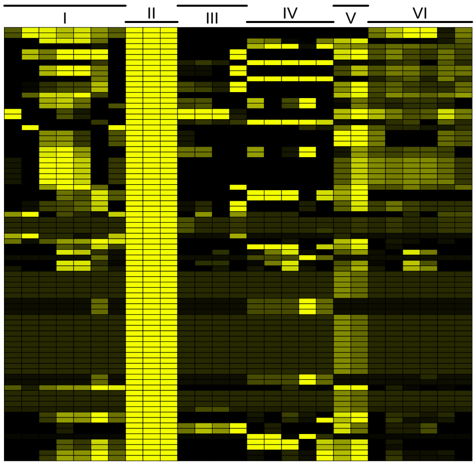

Cluster 31 (n=135)

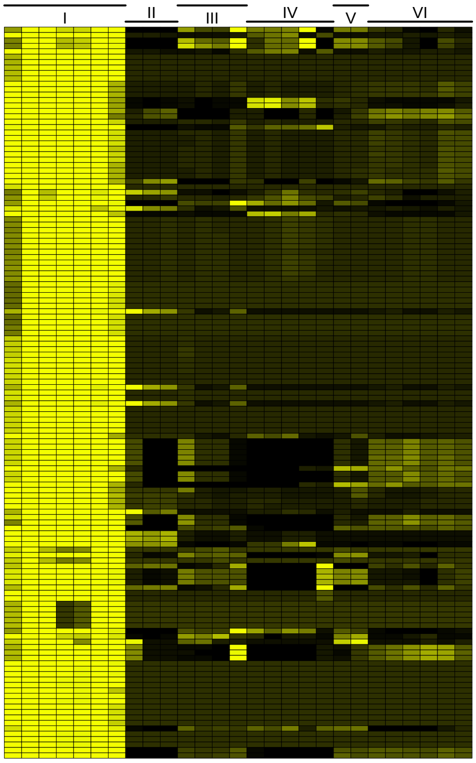

Cluster 32 (n=92)

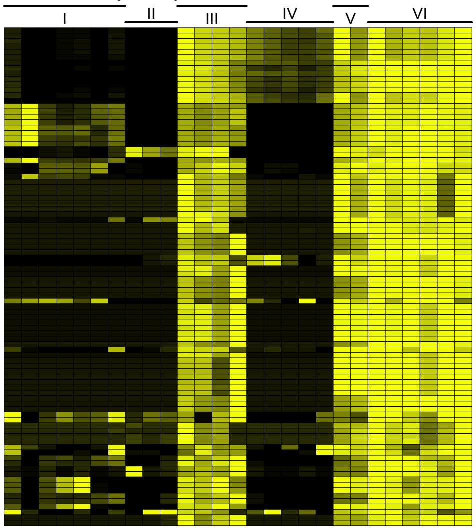

Cluster 33 (n=223)

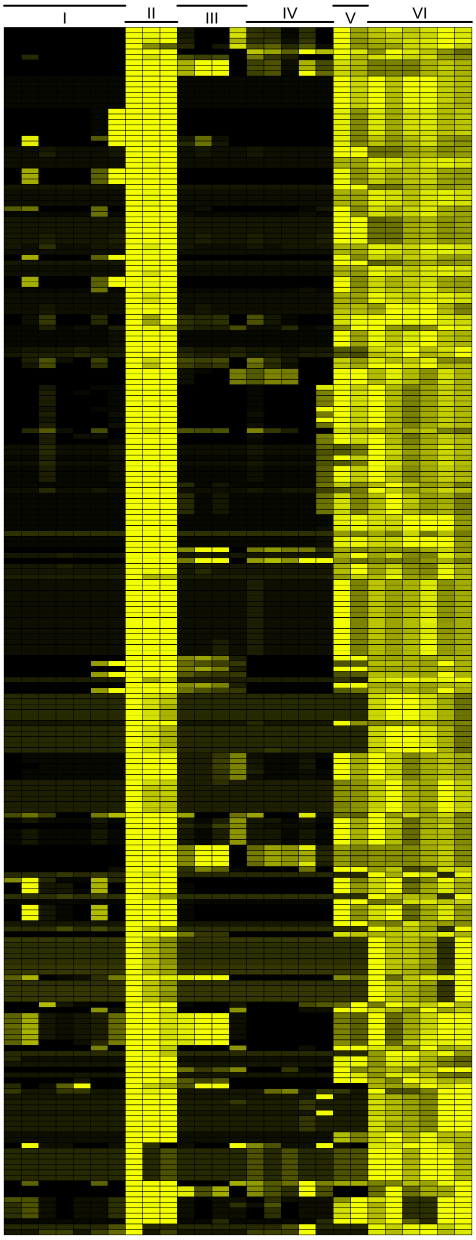

Cluster 34 (n=94)

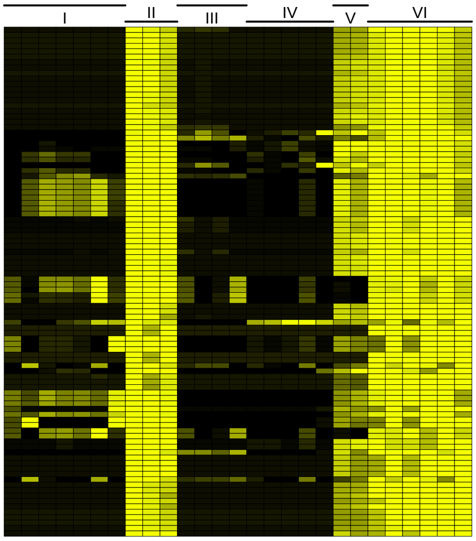

Cluster 35 (n=104)

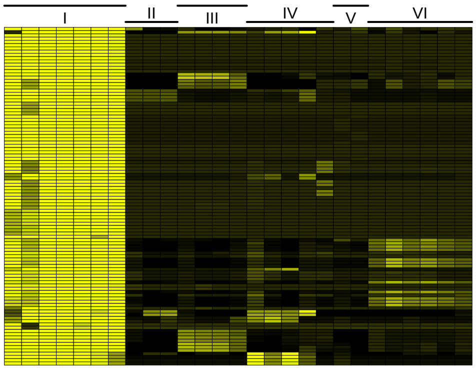

Cluster 36 (n=44)

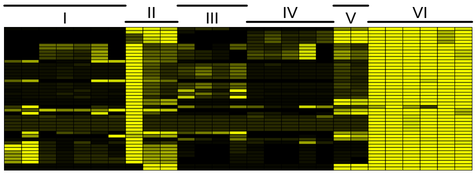

Cluster 37 (n=388)

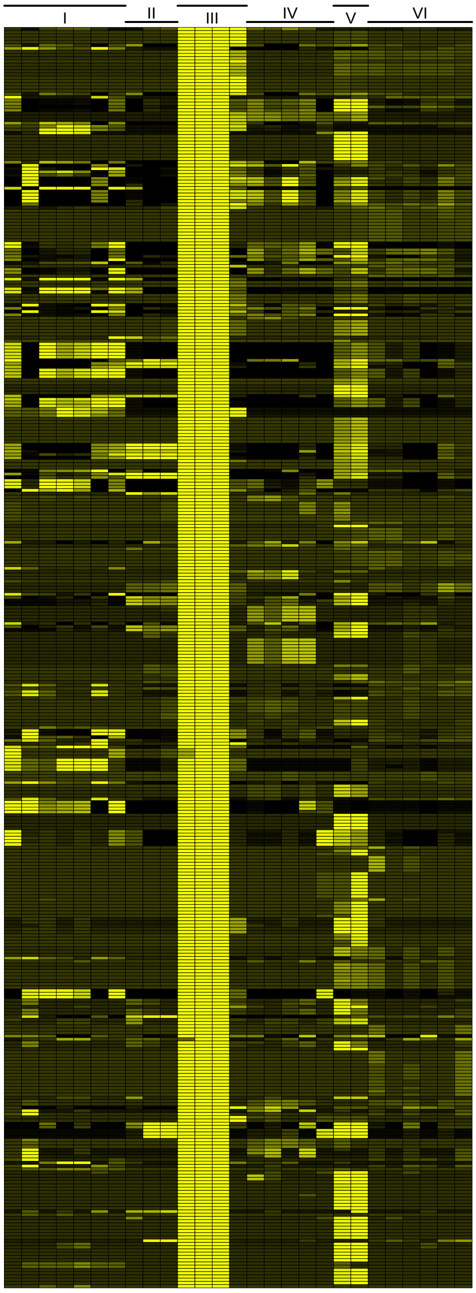

Cluster 38 (n=476)

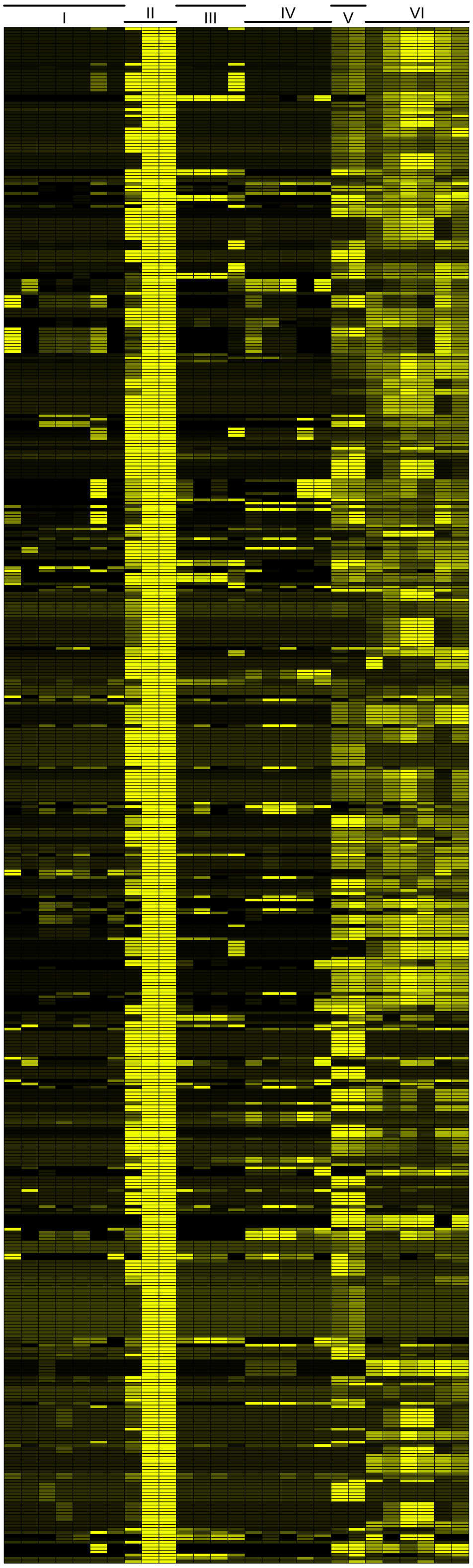

Cluster 39 (n=131)

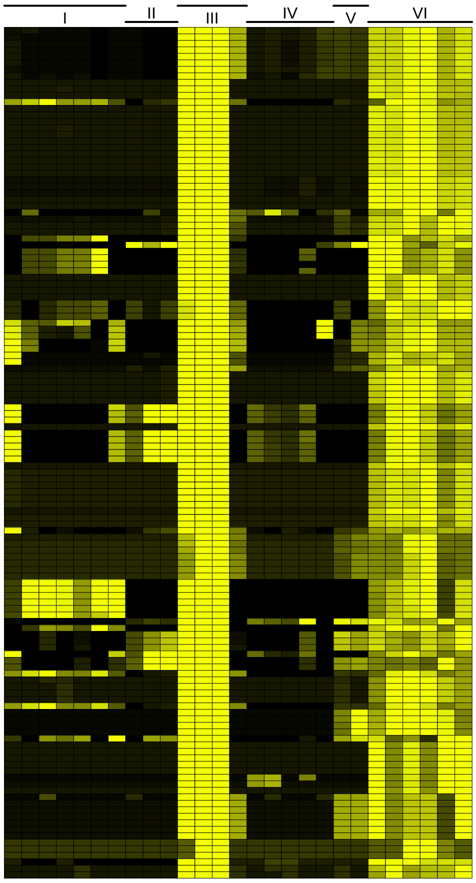

Cluster 41 (n=199)

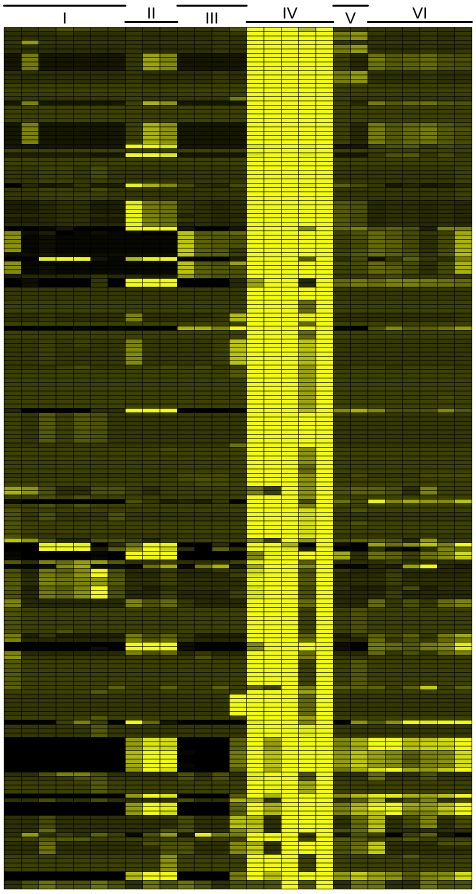

Cluster 40 (n=119)

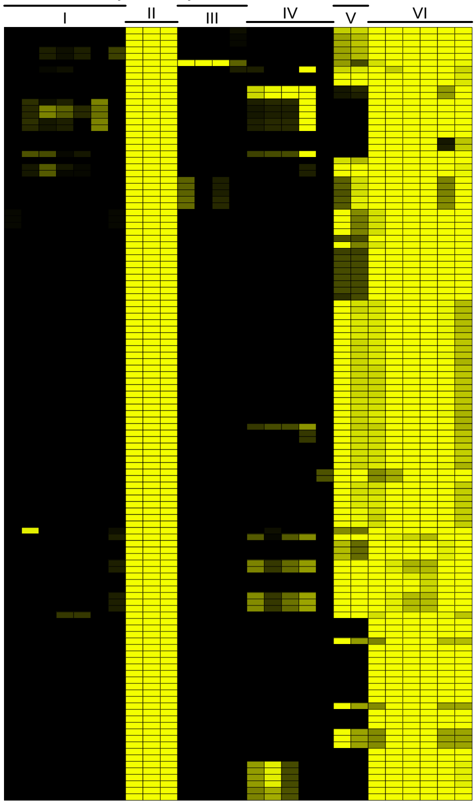

Cluster 42 (n=206)

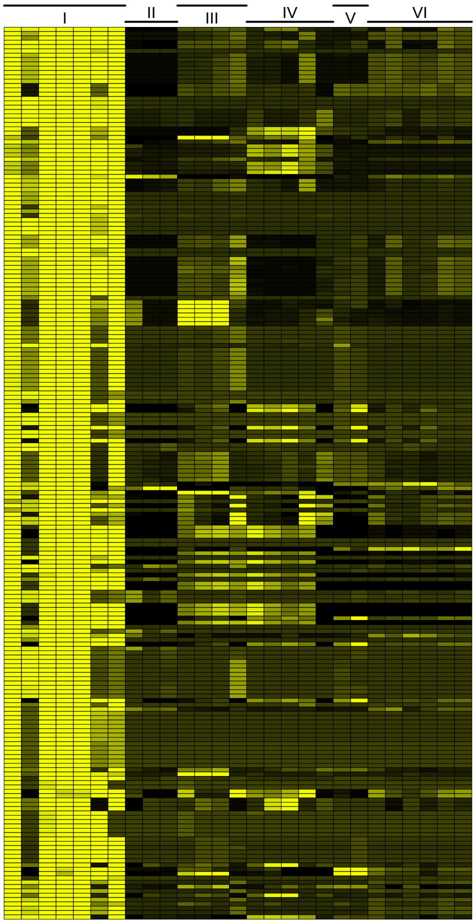

Cluster 43 (n=183)

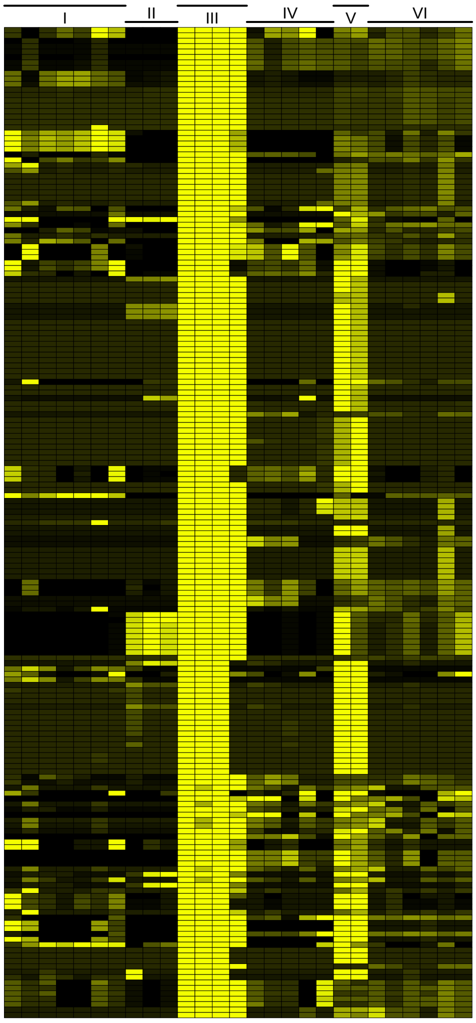

Cluster 44 (n=151)

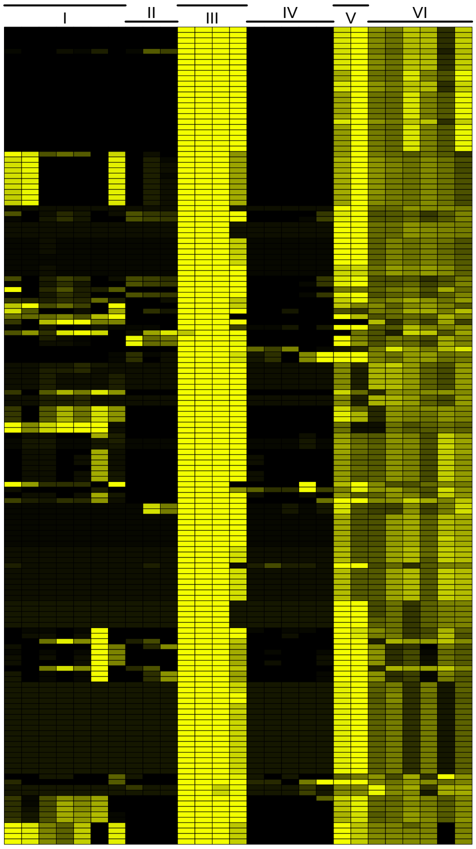

Cluster 45 (n=73)

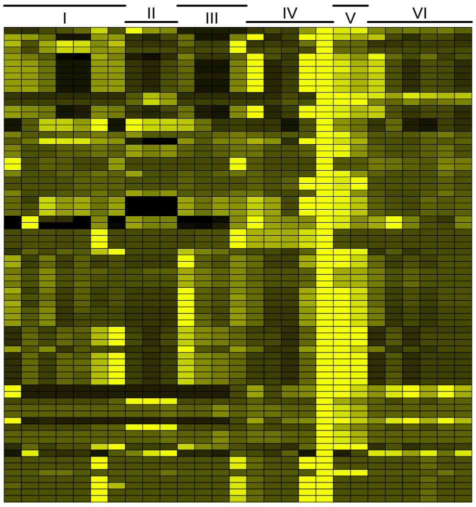

Cluster 46 (n=181)

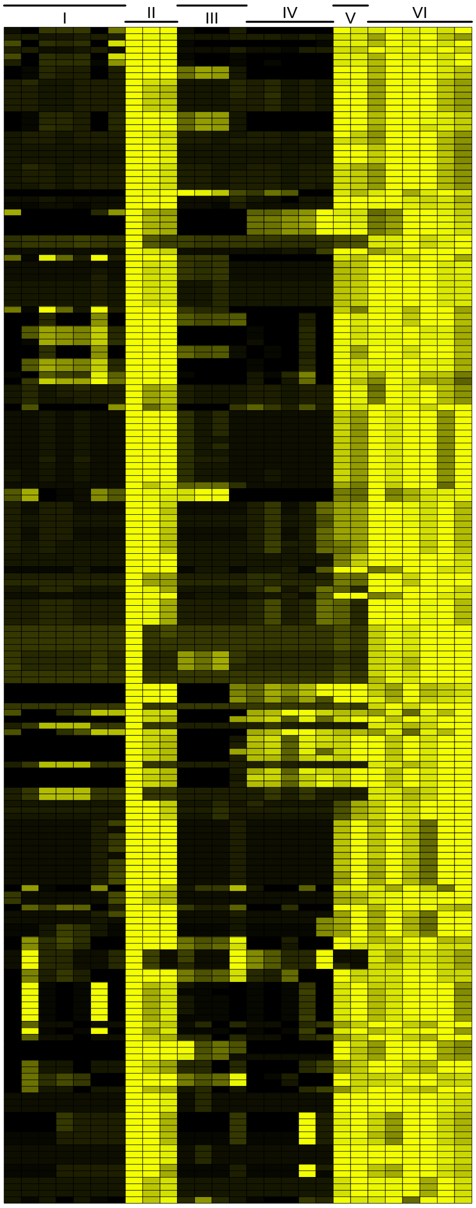

Cluster 47 (n=66)

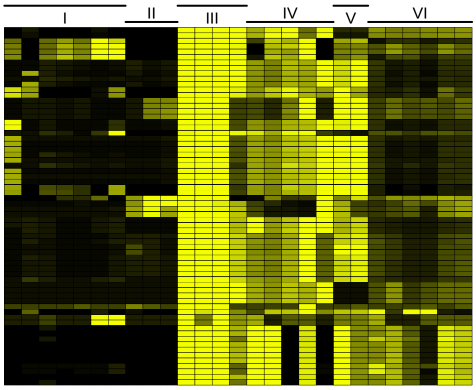

Cluster 48 (n=164)

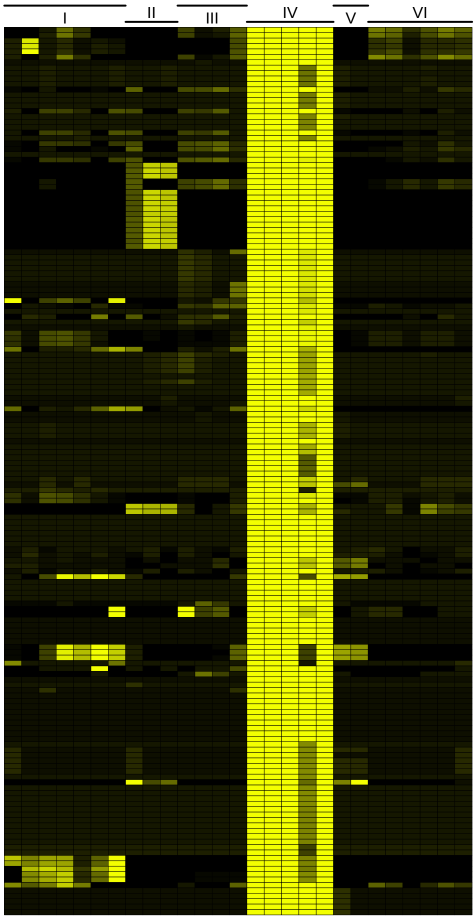

Cluster 49 (n=107)

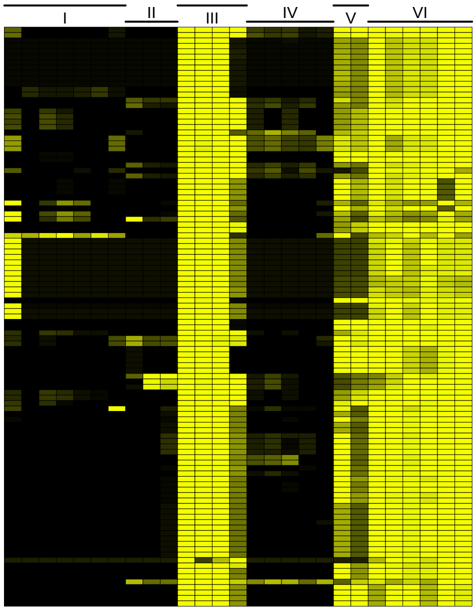

Cluster 50 (n=72)

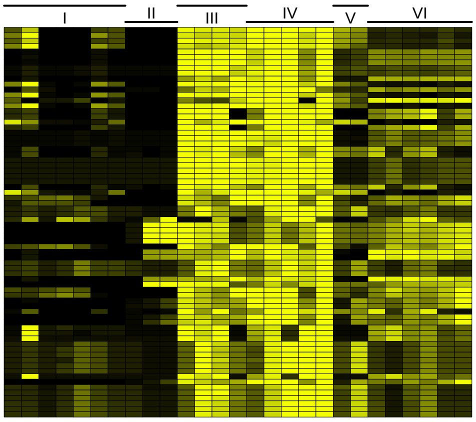

Cluster 51 (n=59)

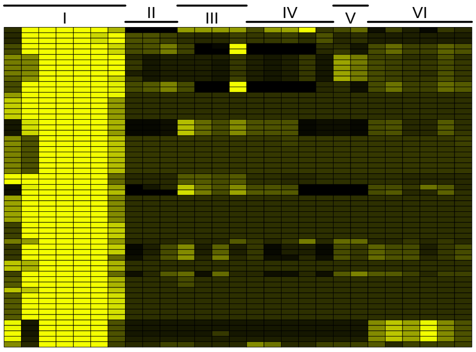

Cluster 52 (n=67)

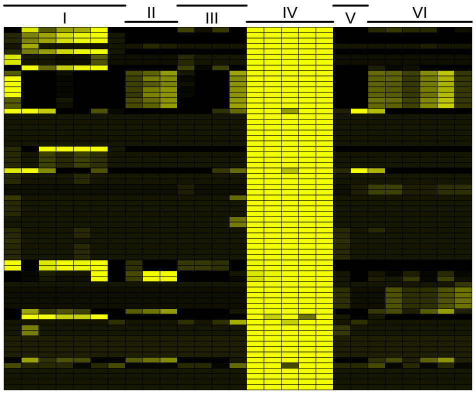

Cluster 53 (n=128)

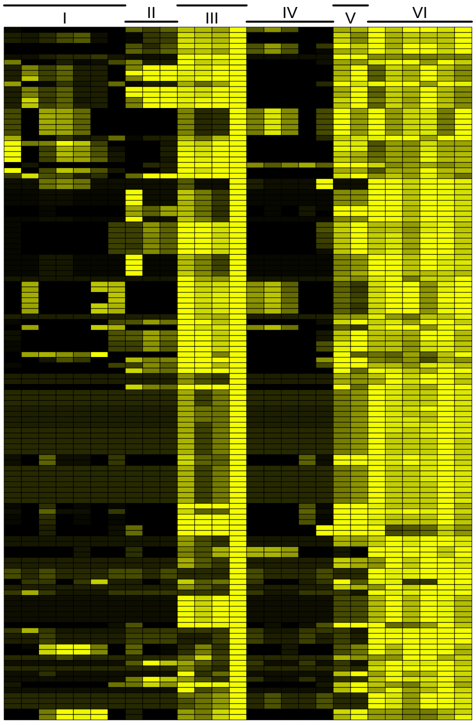

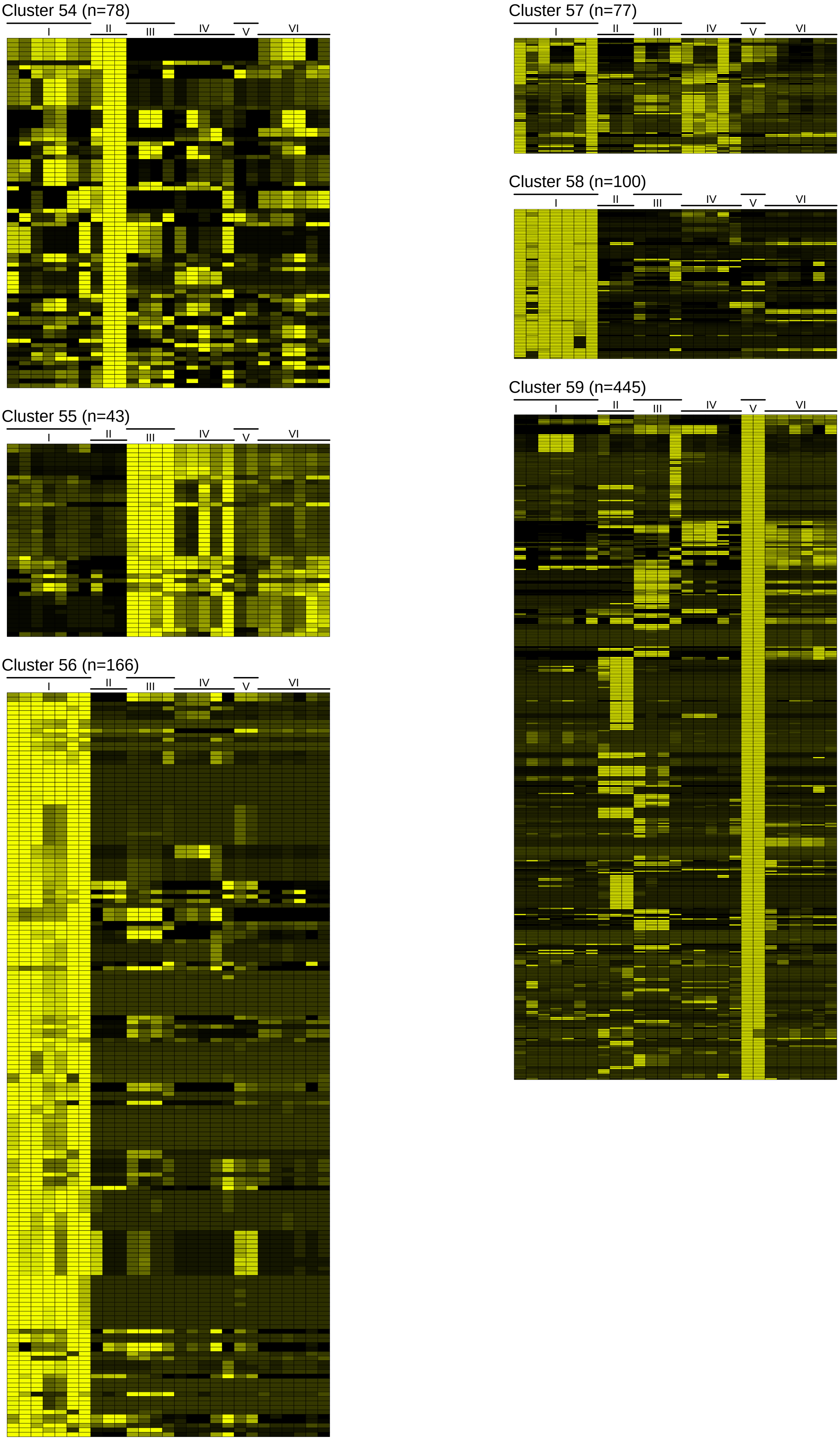

Cluster 54 (n=78)
I
II
III
IV
V
VI
Cluster 55 (n=43)
I
II
III
IV
V
VI
Cluster 56 (n=166)
I
II
III
IV
V
VI
Cluster 57 (n=77)
I
II
III
IV
V
VI
Cluster 58 (n=100)
I
II
III
IV
V
VI
Cluster 59 (n=445)
I
II
III
IV
V
VI

Cluster 60 (n=120)

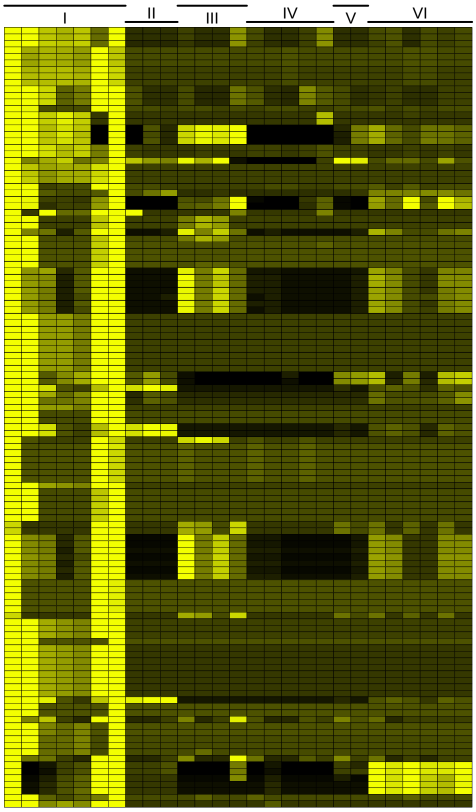

Cluster 61 (n=147)

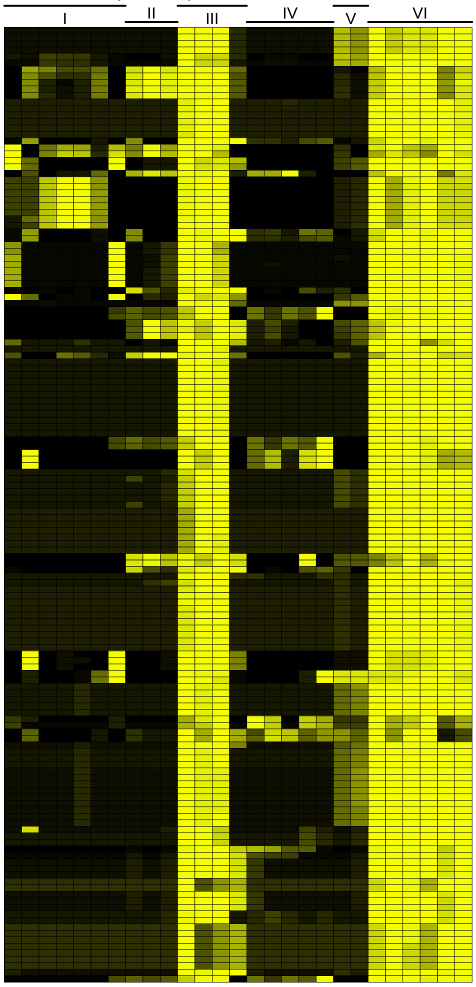

Cluster 62 (n=272)

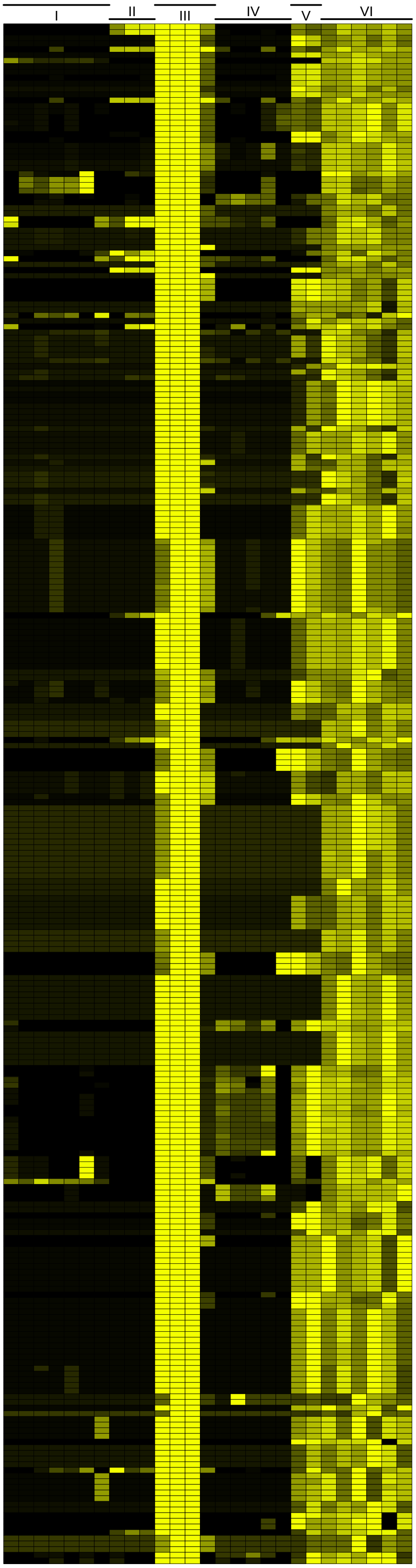

Cluster 63 (n=164)

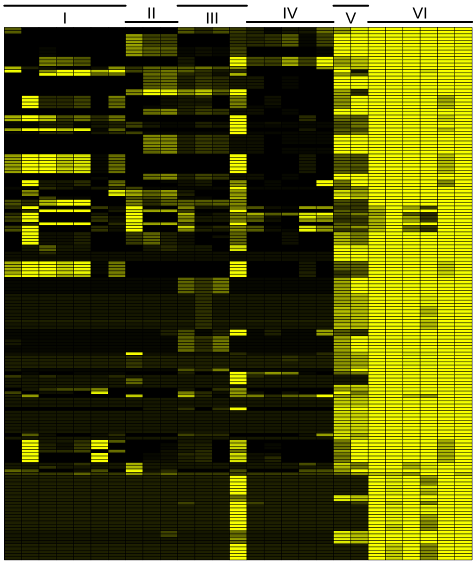

Cluster 64 (n=312)

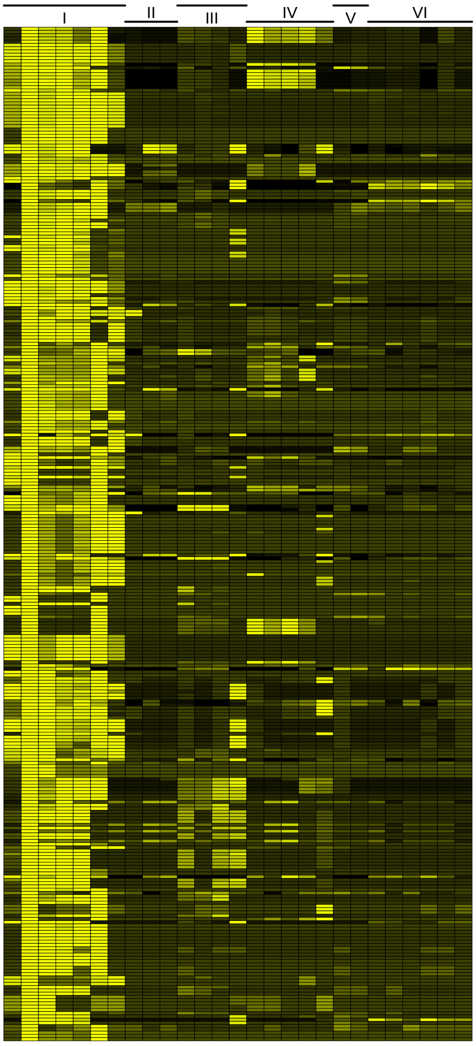

Cluster 65 (n=251)

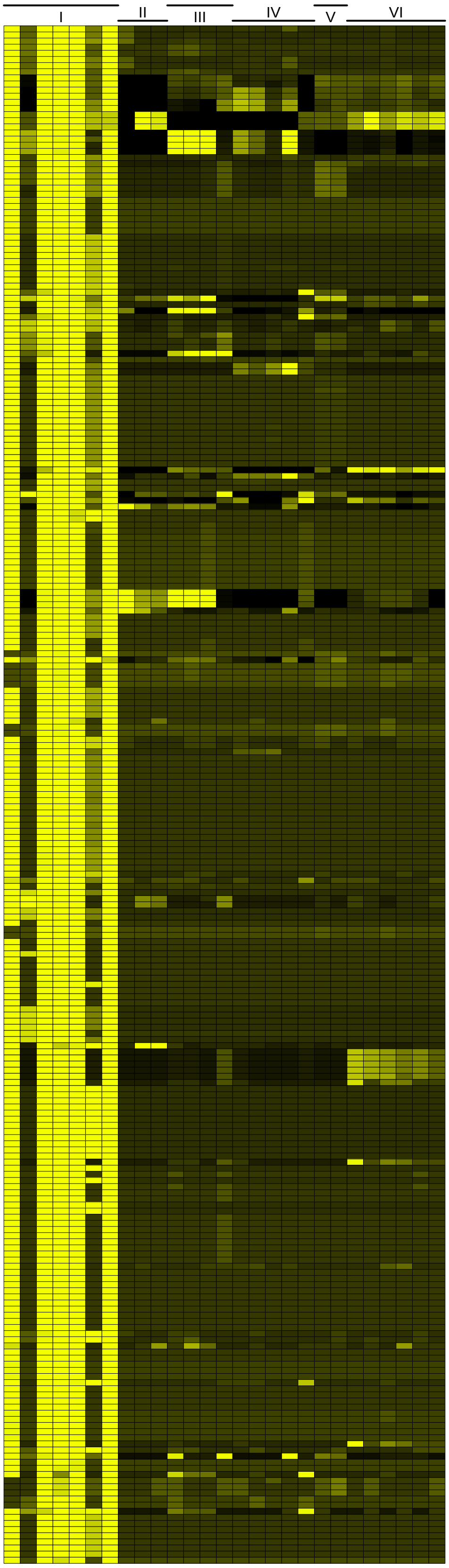

Cluster 66 (n=271)

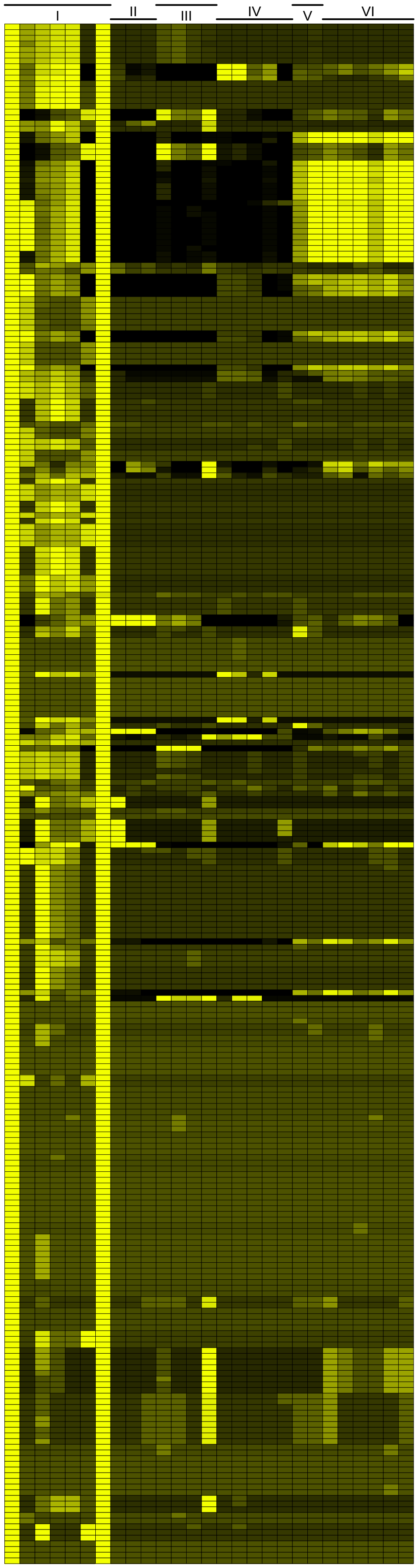

Cluster 67 (n=130)

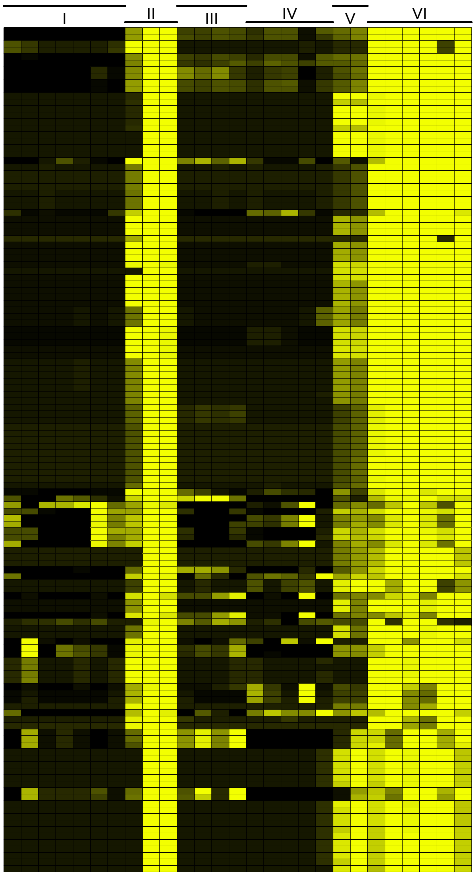

Cluster 68 (n=108)

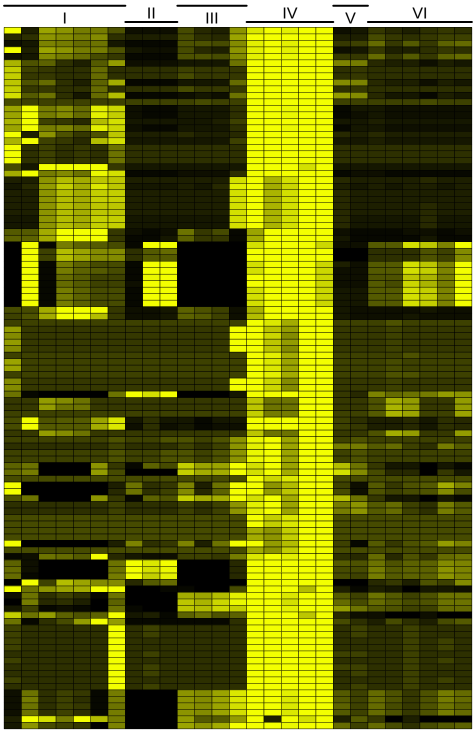

Cluster 69 (n=296)

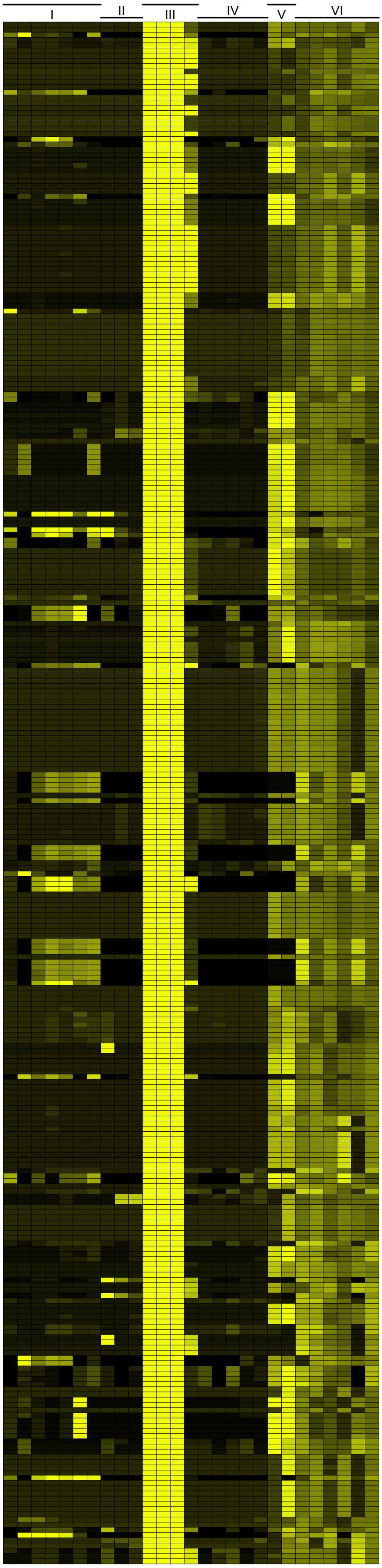

Cluster 70 (n=169)

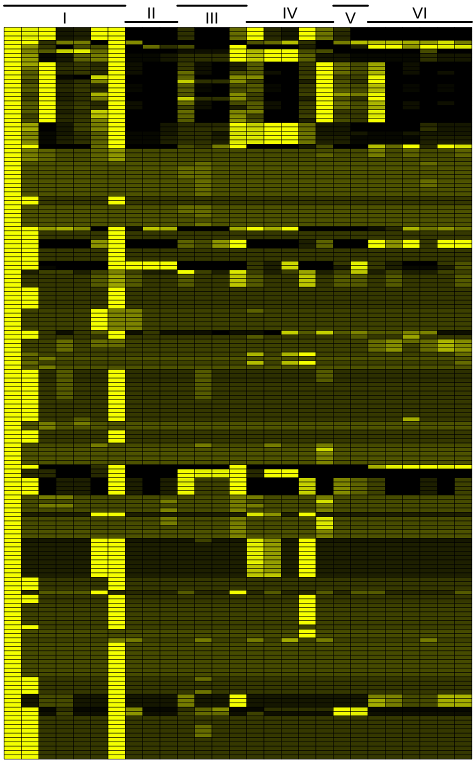

Cluster 71 (n=262)

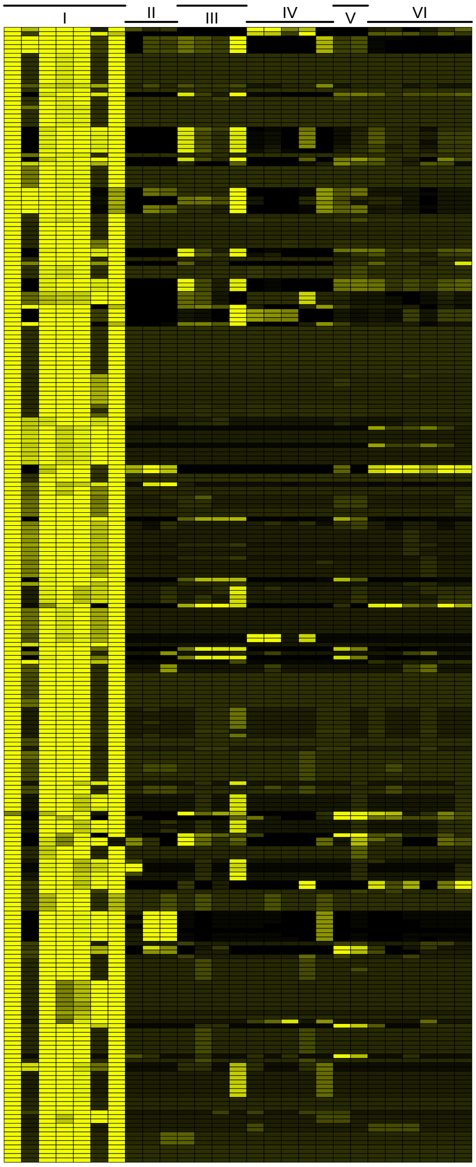

Cluster 72 (n=227)

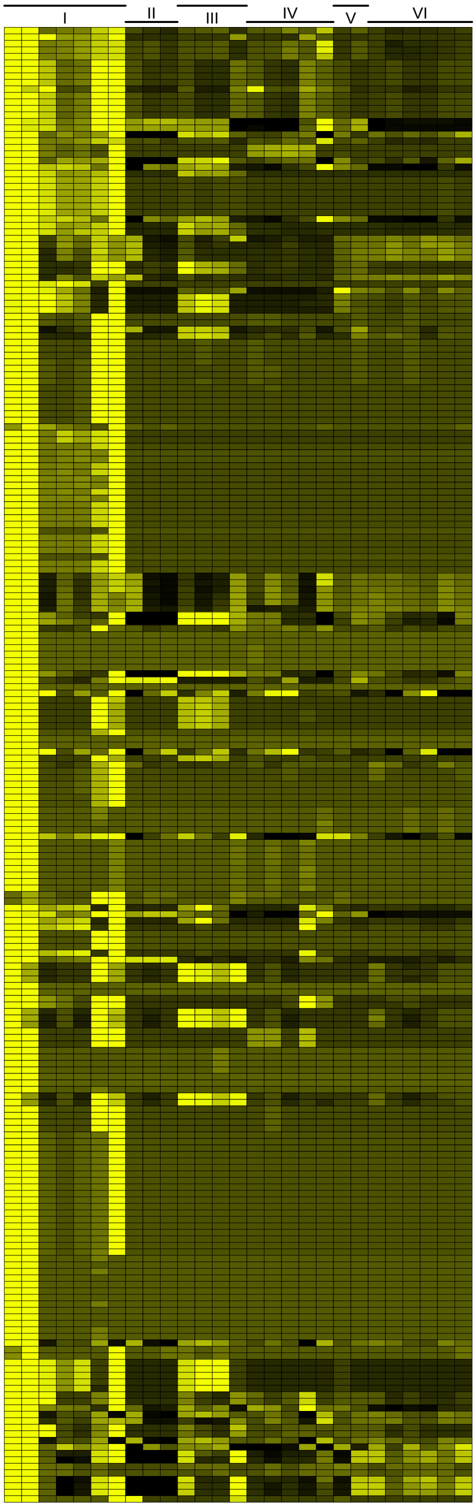

Cluster 73 (n=47)

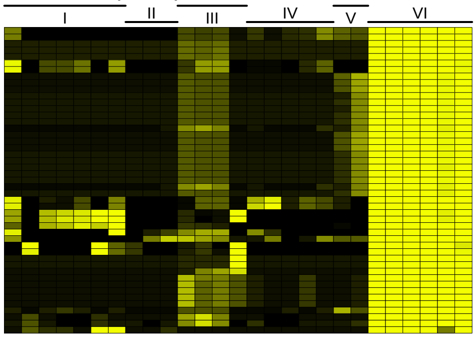

Cluster 74 (n=116)

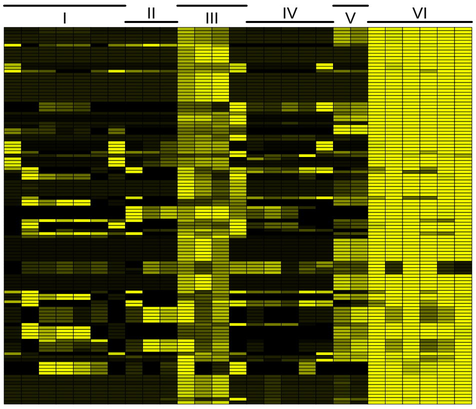

Cluster 75 (n=78)

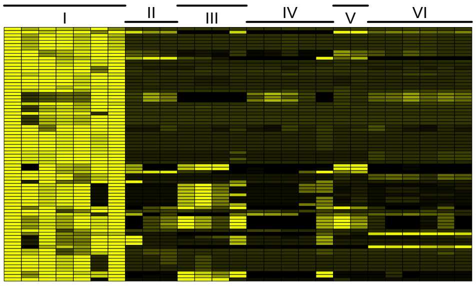

Cluster 76 (n=22)

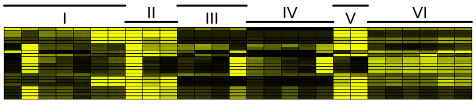

Cluster 77 (n=298)

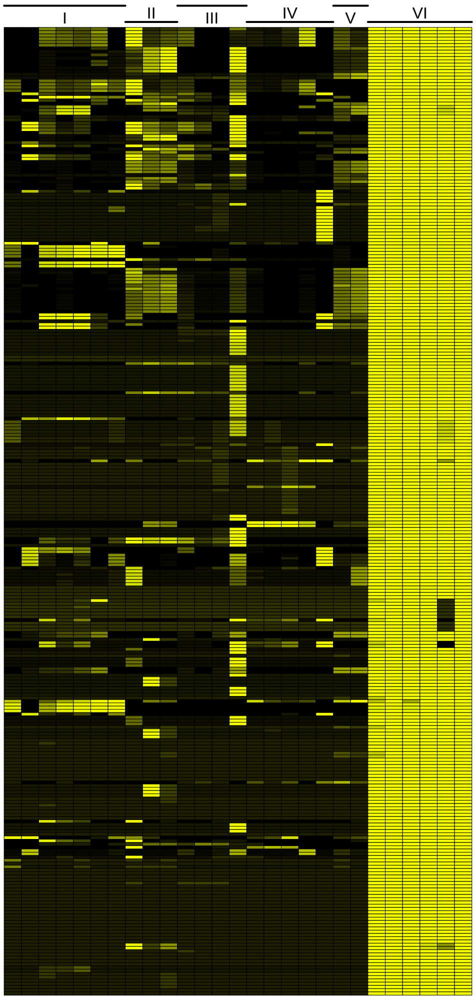

Cluster 78 (n=68)

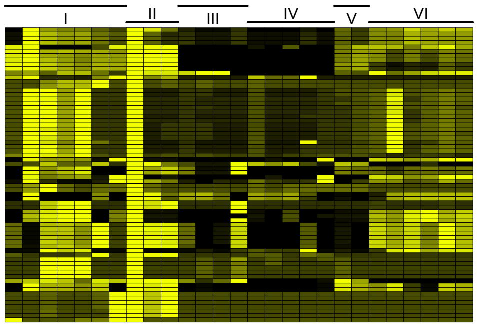

Cluster 79 (n=154)

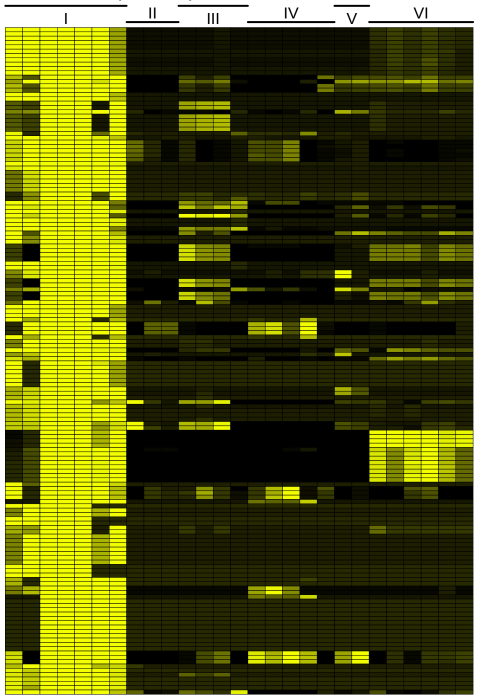

Cluster 80 (n=212)

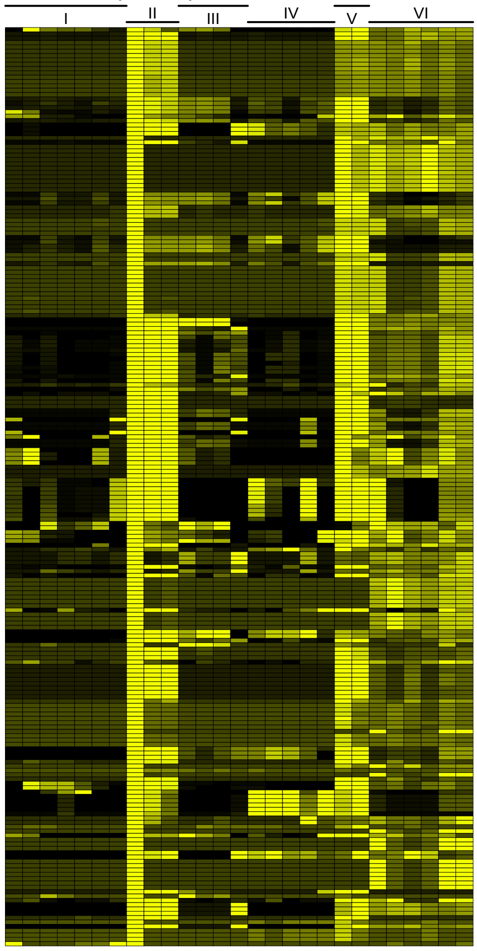

Cluster 81 (n=31)

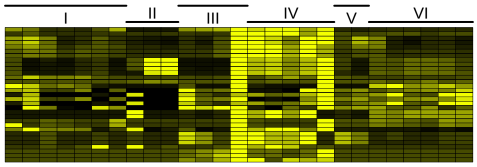

Cluster 82 (n=107)

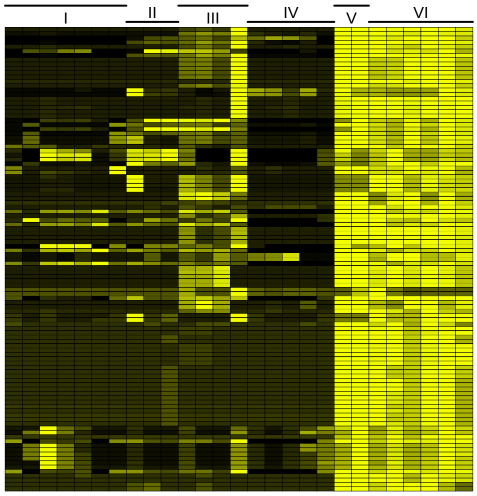

Cluster 83 (n=250)

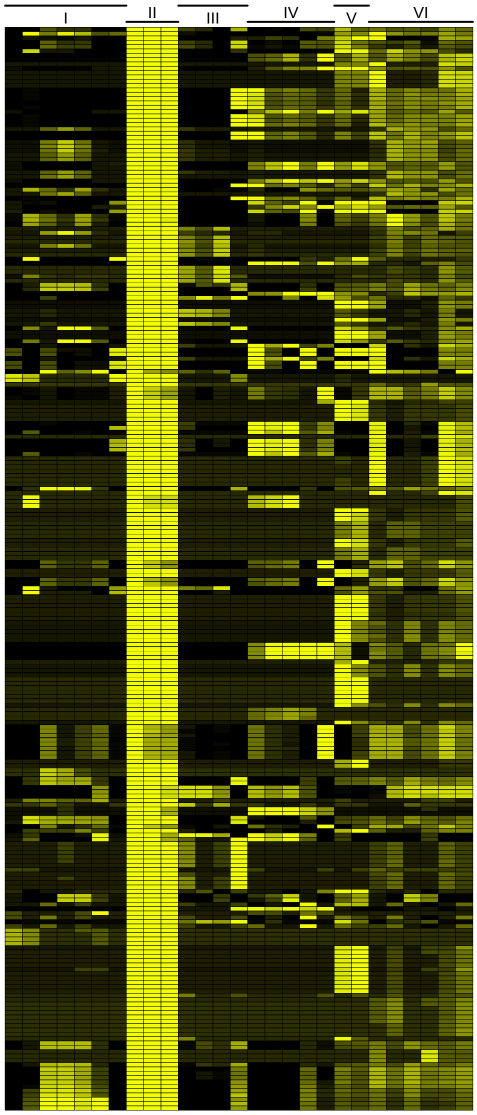

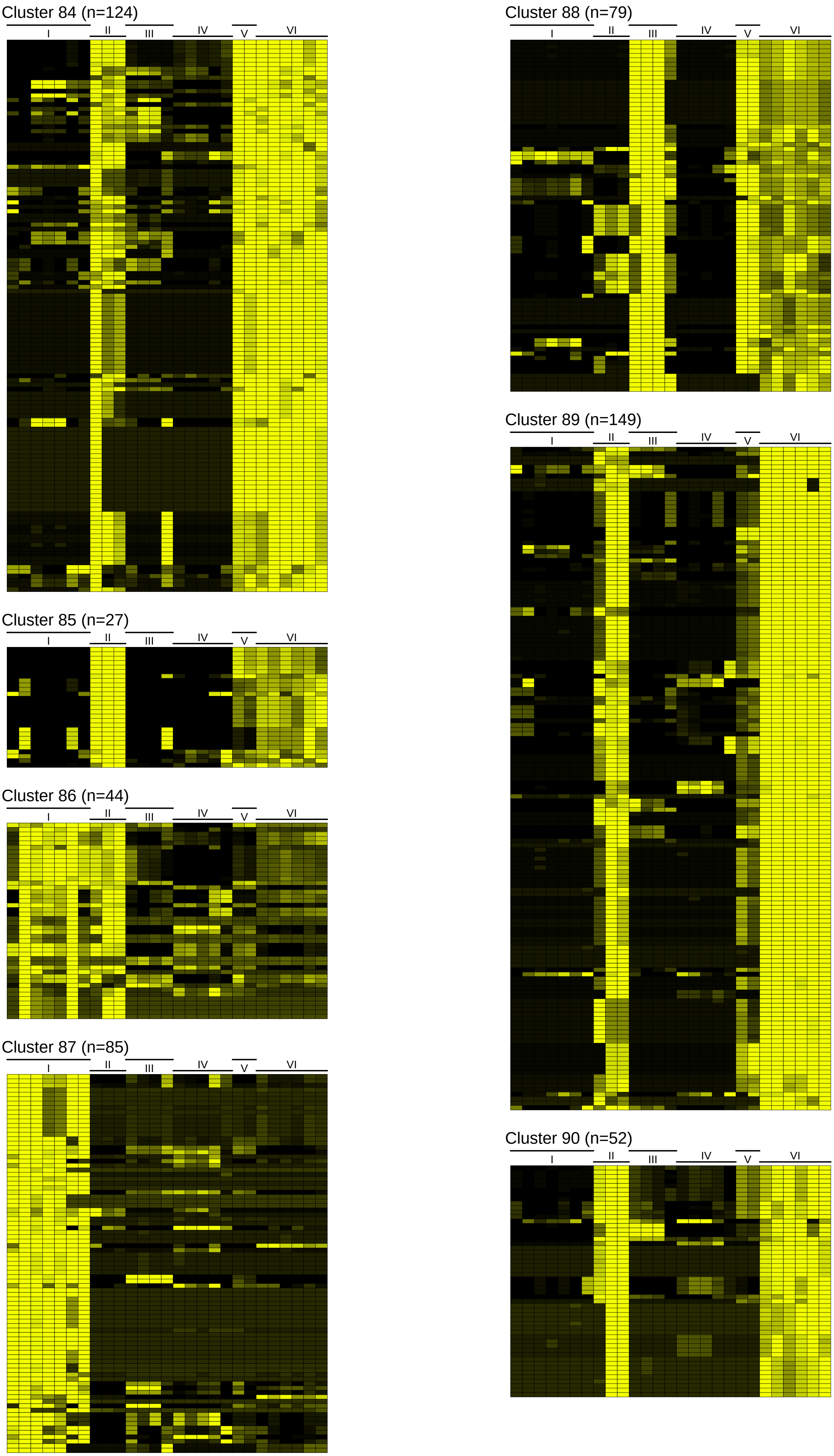
Cluster 84 (n=124)
I II III IV V VI
Cluster 85 (n=27)
I II III IV V VI
Cluster 86 (n=44)
I II III IV V VI
Cluster 87 (n=85)
I II III IV V VI
Cluster 88 (n=79)
I II III IV V VI
Cluster 89 (n=149)
I II III IV V VI
Cluster 90 (n=52)
I II III IV V VI

Cluster 91 (n=97)

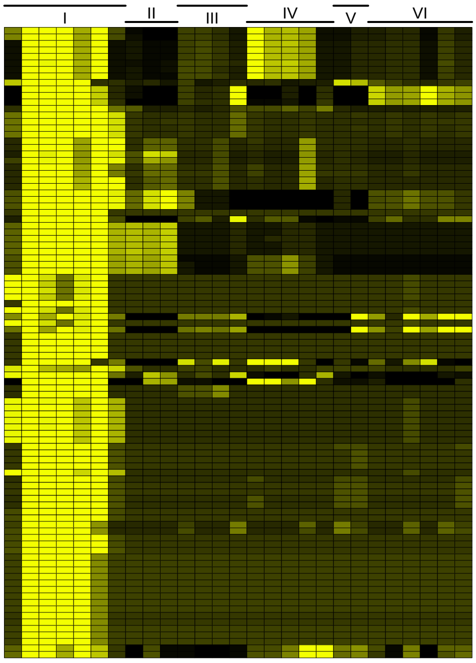

Cluster 92 (n=26)

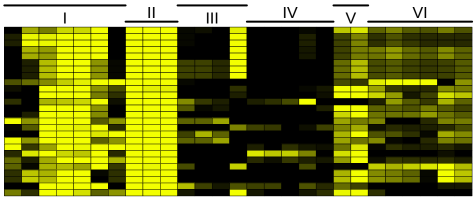

Cluster 93 (n=118)

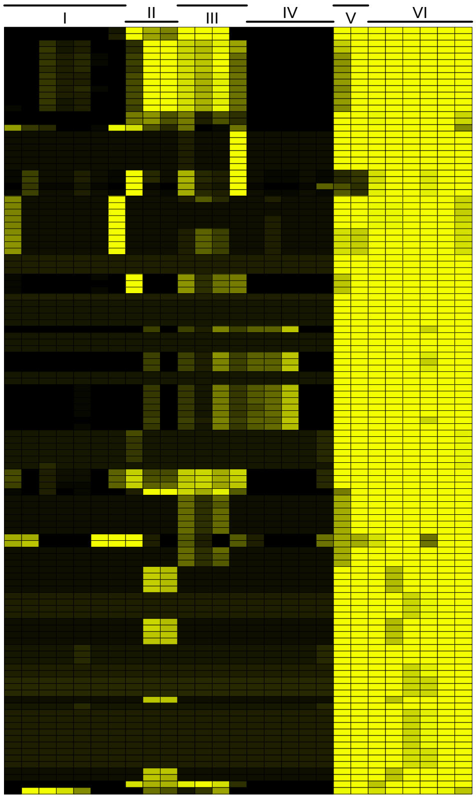

Cluster 94 (n=191)

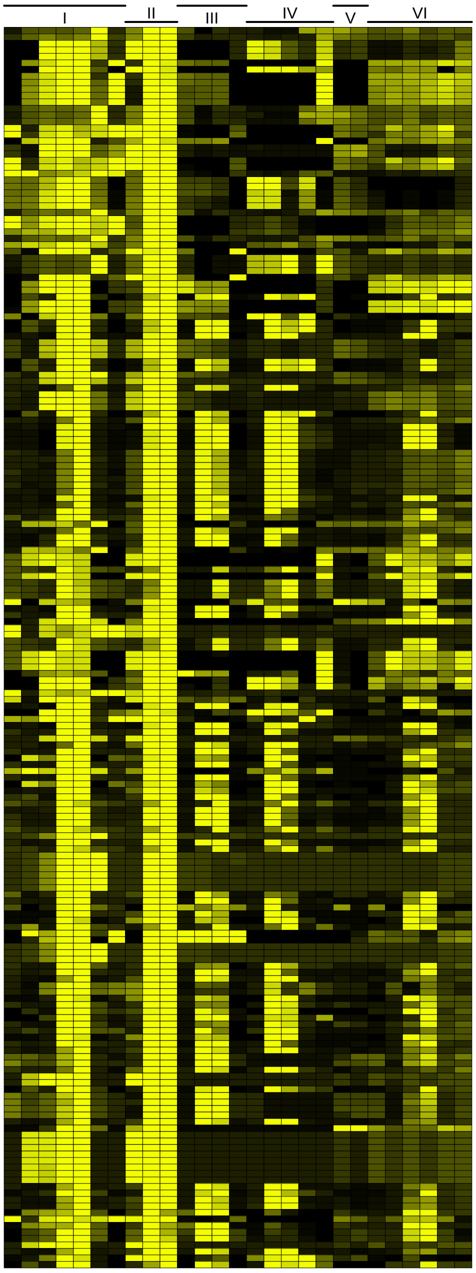

Cluster 95 (n=58)

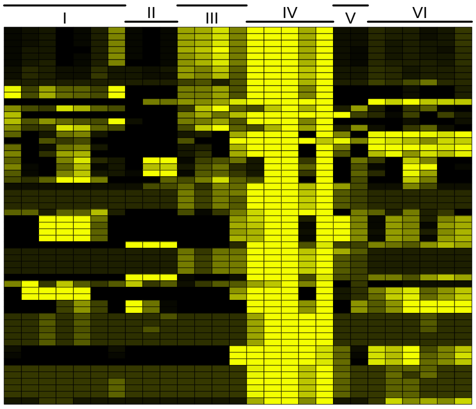

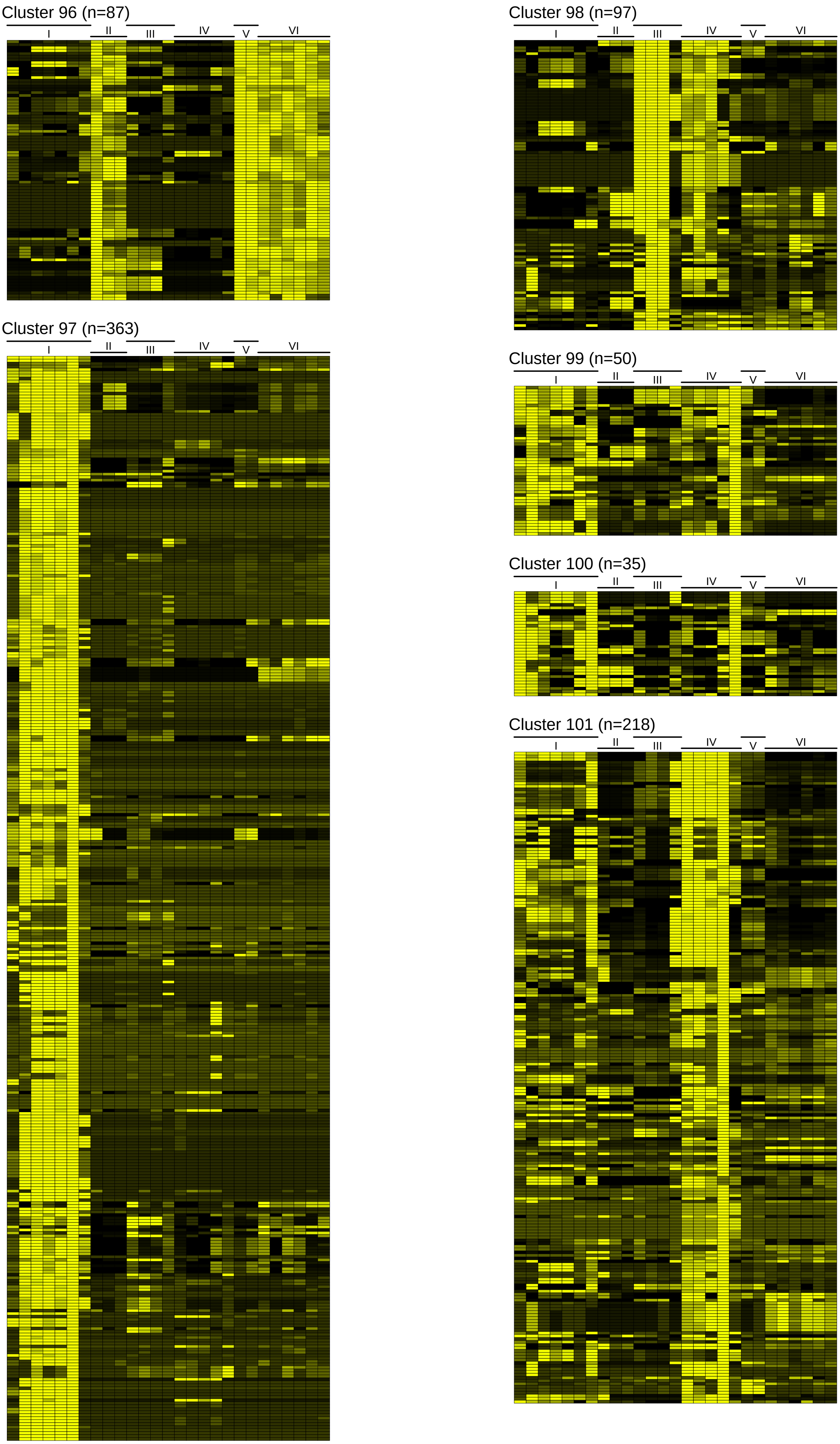

Cluster 96 (n=87)
I II III IV V VI
Cluster 97 (n=363)
I II III IV V VI
Cluster 98 (n=97)
I II III IV V VI
Cluster 99 (n=50)
I II III IV V VI
Cluster 100 (n=35)
I II III IV V VI
Cluster 101 (n=218)
I II III IV V VI

Cluster 102 (n=145)

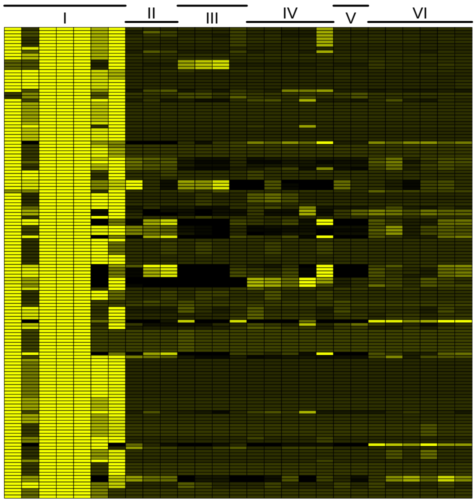

Cluster 103 (n=46)

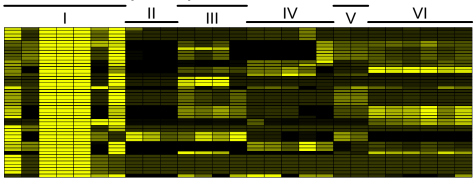

Cluster 104 (n=392)

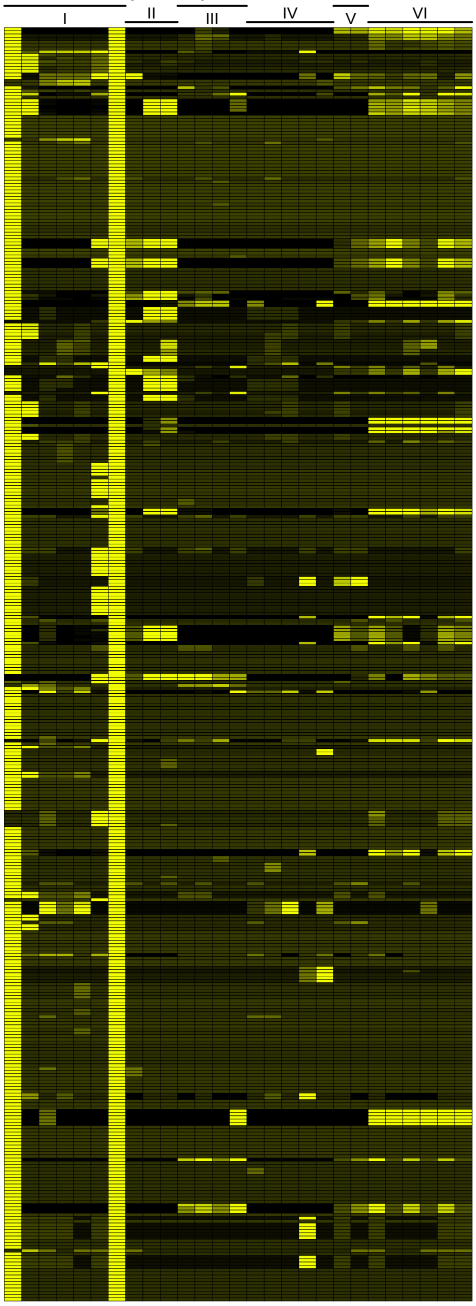

Cluster 105 (n=120)

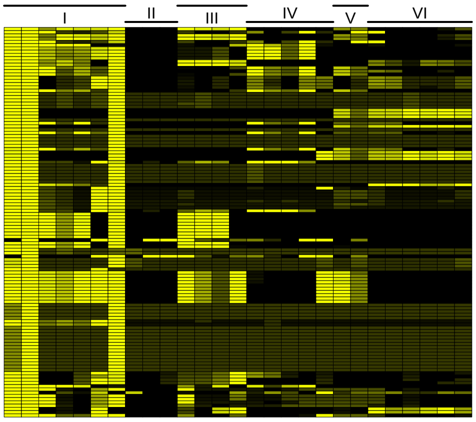

Cluster 106 (n=25)

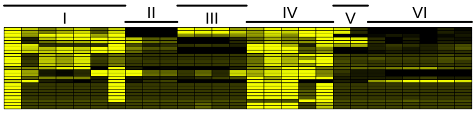

Cluster 107 (n=428)

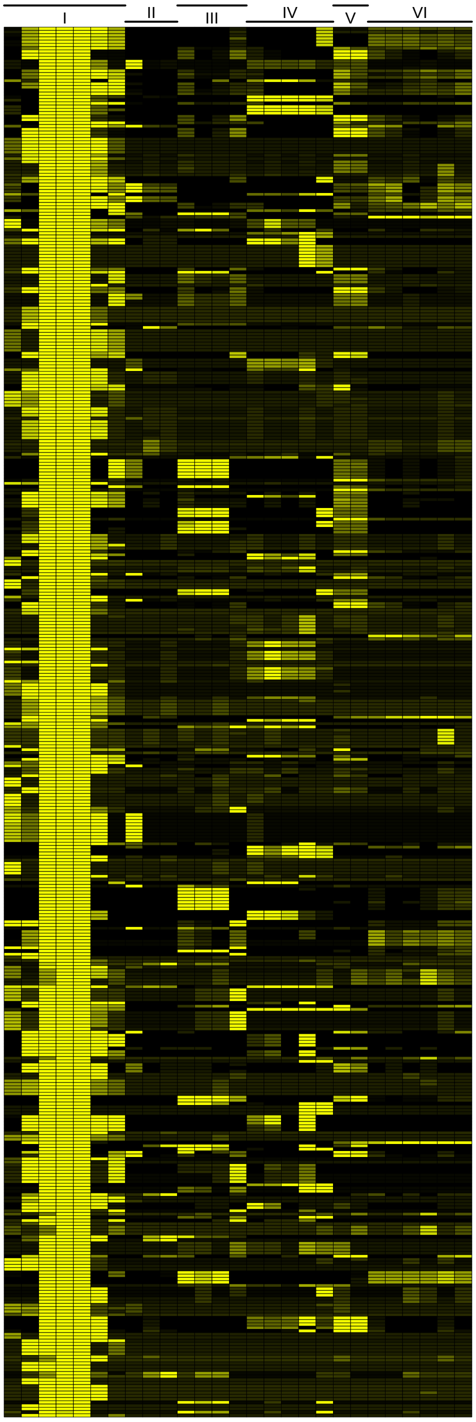

Cluster 108 (n=25)

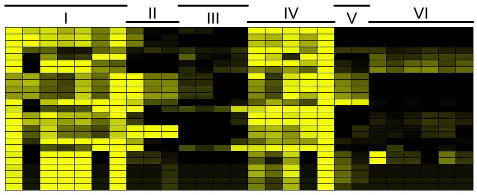

Cluster 109 (n=171)

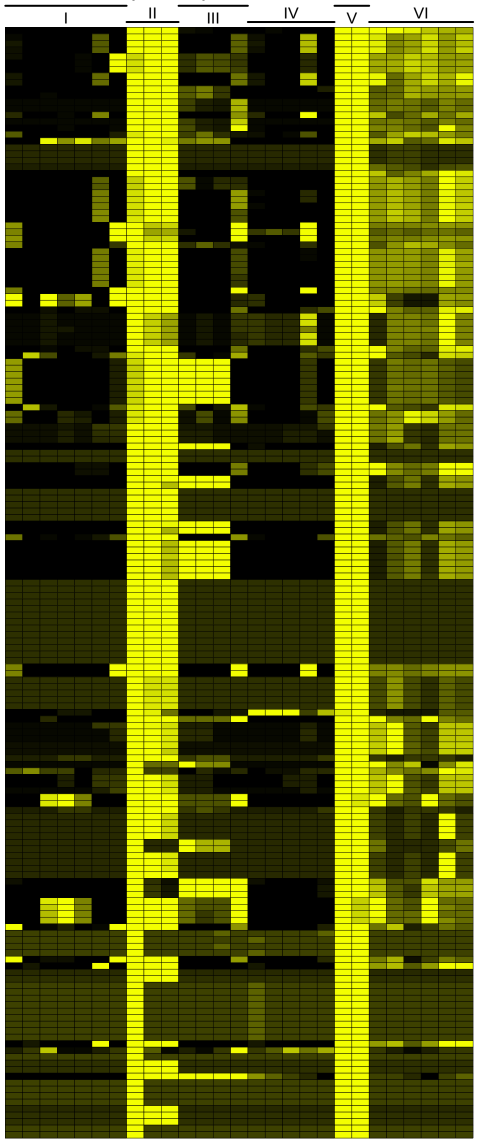

Cluster 110 (n=36)

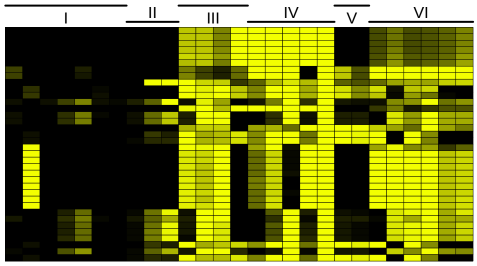

Cluster 111 (n=170)

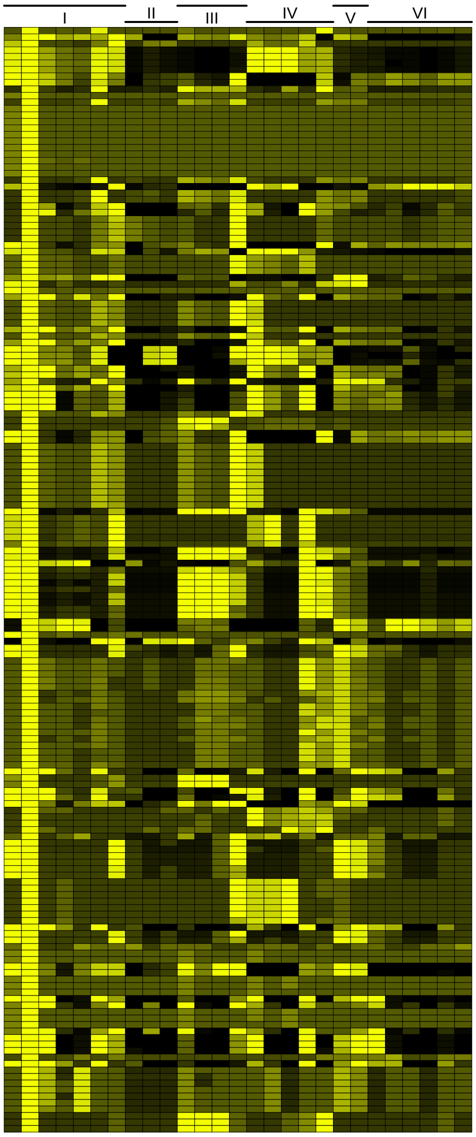

Cluster 112 (n=109)

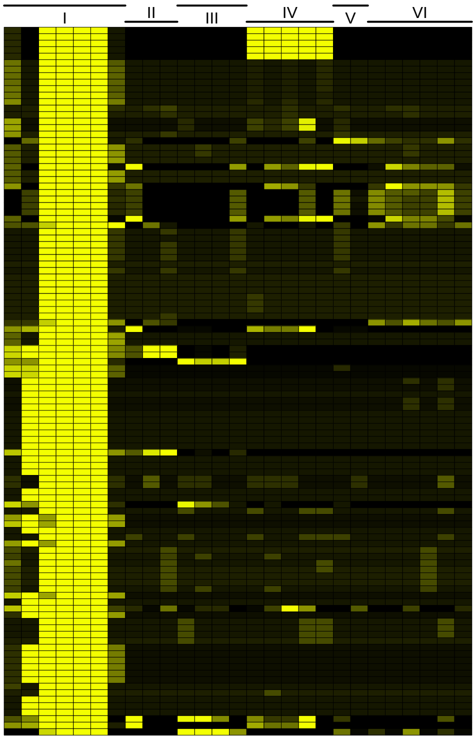

Cluster 113 (n=9)

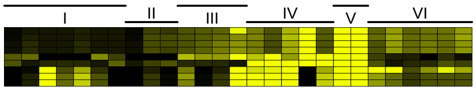

Cluster 114 (n=36)

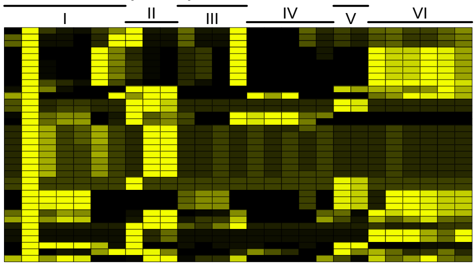

Cluster 115 (n=33)

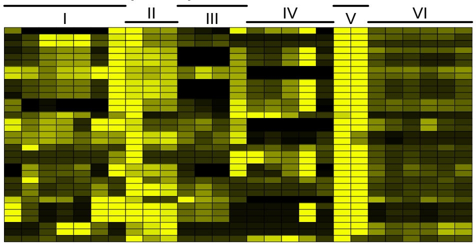

Cluster 116 (n=17)

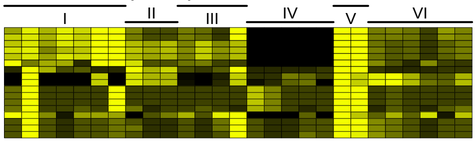

Cluster 117 (n=10)

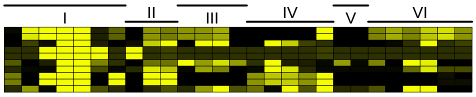

Cluster 118 (n=36)

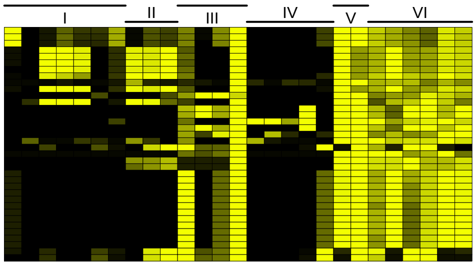

Cluster 119 (n=39)

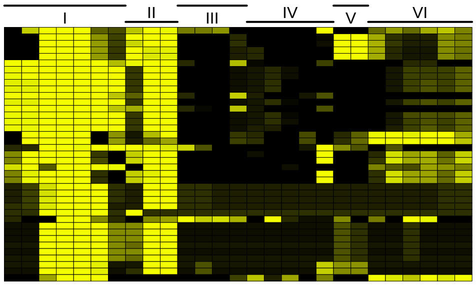

Cluster 120 (n=36)

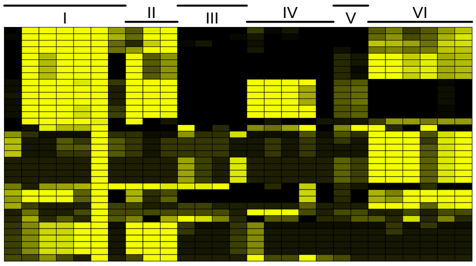

Cluster 121 (n=61)

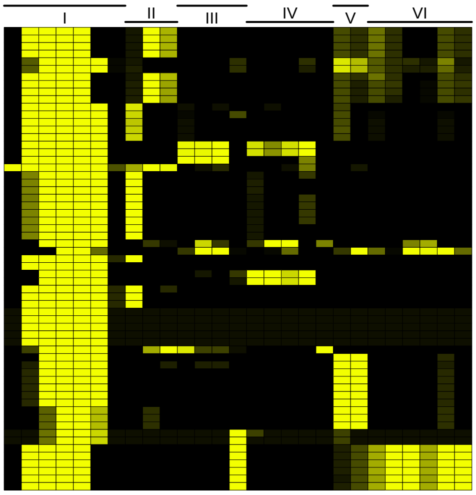

Cluster 122 (n=32)

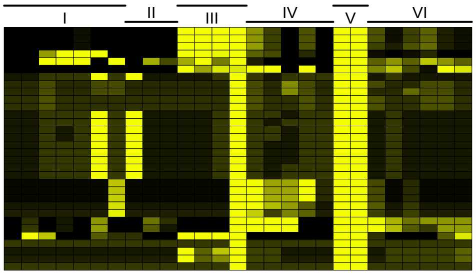

Cluster 123 (n=8)

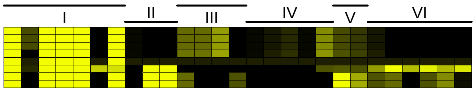

Cluster 124 (n=25)

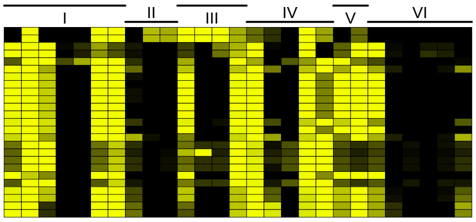

Cluster 125 (n=55)

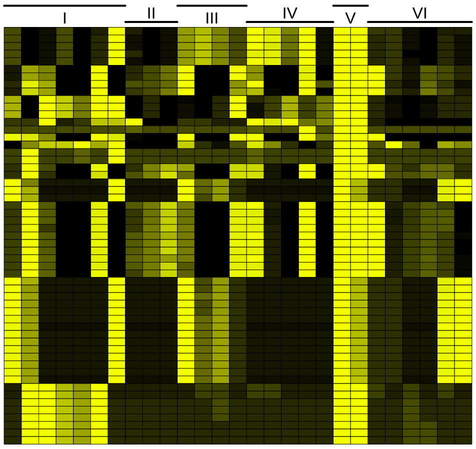

Supplement: Supplementary file 4 [file 1678-8060-mioc-116-e200538-s5.pdf]
