# Supplementary material for: A new Trypanosoma cruzi genotyping method enables high resolution evolutionary analyses
Source: Mem Inst Oswaldo Cruz. 2021 Aug 30;116:e200538. doi: 10.1590/0074-02760200538 (PMC8405150; doi:10.1590/0074-02760200538)

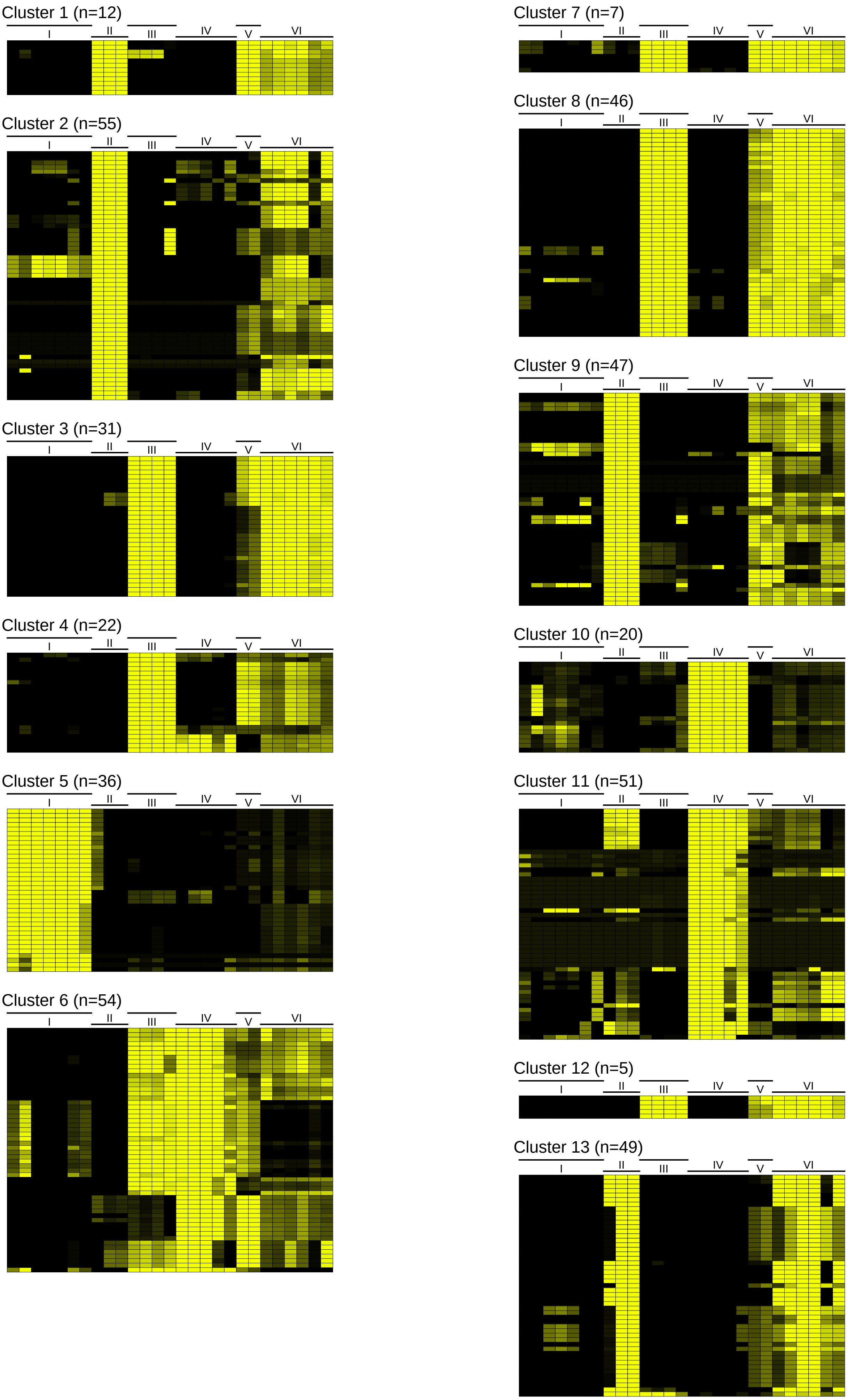
Cluster 1 (n=12)
I II III IV V VI
Cluster 2 (n=55)
I II III IV V VI
Cluster 3 (n=31)
I II III IV V VI
Cluster 4 (n=22)
I II III IV V VI
Cluster 5 (n=36)
I II III IV V VI
Cluster 6 (n=54)
I II III IV V VI
Cluster 7 (n=7)
I II III IV V VI
Cluster 8 (n=46)
I II III IV V VI
Cluster 9 (n=47)
I II III IV V VI
Cluster 10 (n=20)
I II III IV V VI
Cluster 11 (n=51)
I II III IV V VI
Cluster 12 (n=5)
I II III IV V VI
Cluster 13 (n=49)
I II III IV V VI

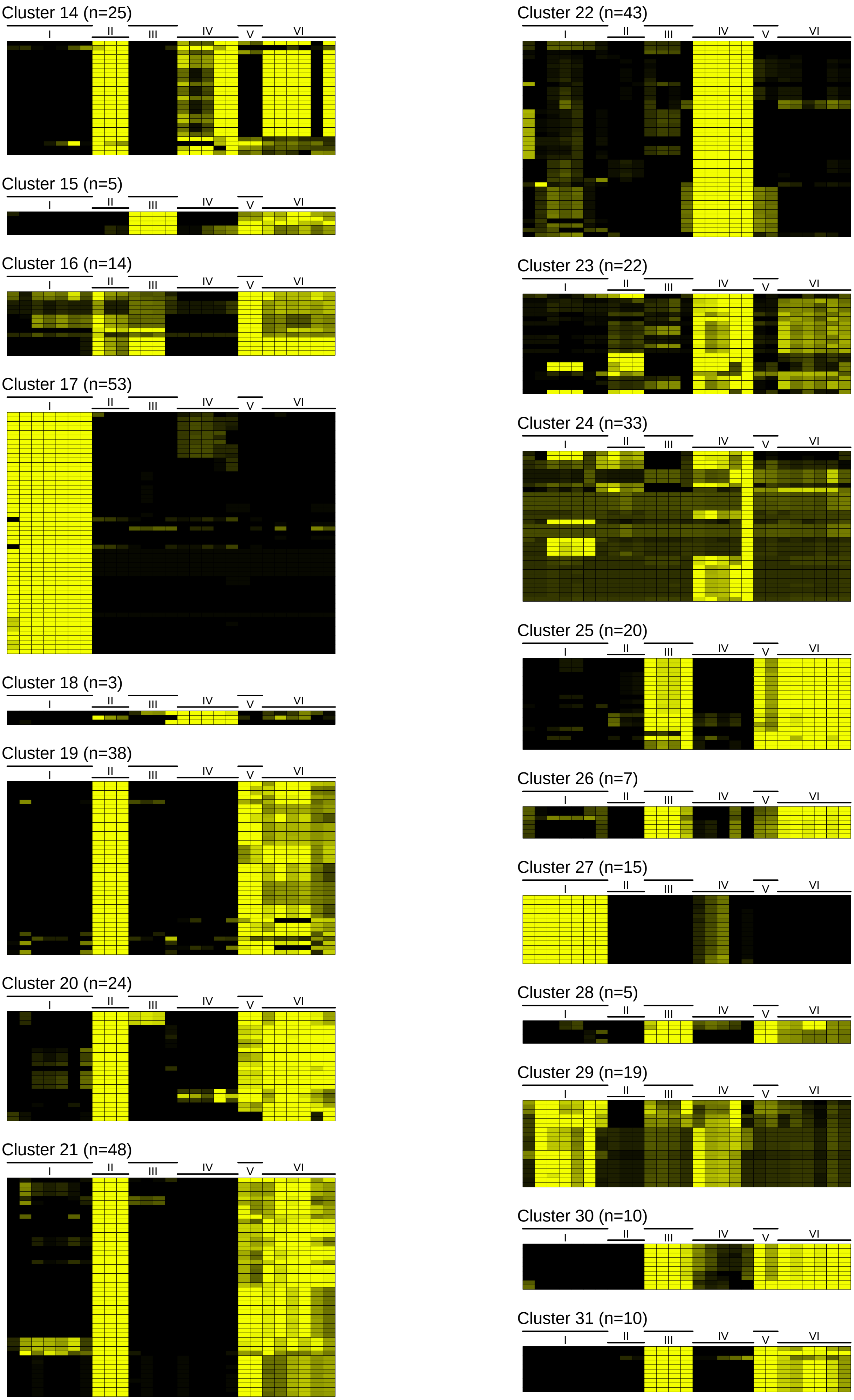

Cluster 14 (n=25)
I II III IV V VI
Cluster 15 (n=5)
I II III IV V VI
Cluster 16 (n=14)
I II III IV V VI
Cluster 17 (n=53)
I II III IV V VI
Cluster 18 (n=3)
I II III IV V VI
Cluster 19 (n=38)
I II III IV V VI
Cluster 20 (n=24)
I II III IV V VI
Cluster 21 (n=48)
I II III IV V VI
Cluster 22 (n=43)
I II III IV V VI
Cluster 23 (n=22)
I II III IV V VI
Cluster 24 (n=33)
I II III IV V VI
Cluster 25 (n=20)
I II III IV V VI
Cluster 26 (n=7)
I II III IV V VI
Cluster 27 (n=15)
I II III IV V VI
Cluster 28 (n=5)
I II III IV V VI
Cluster 29 (n=19)
I II III IV V VI
Cluster 30 (n=10)
I II III IV V VI
Cluster 31 (n=10)
I II III IV V VI

Cluster 32 (n=54)

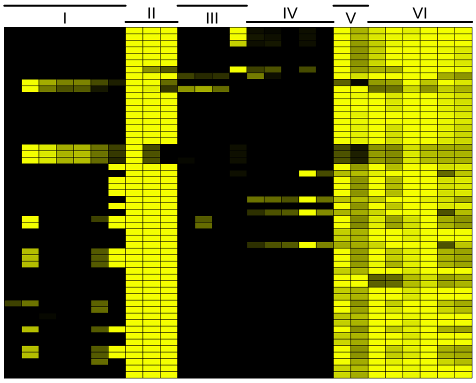

Cluster 33 (n=20)

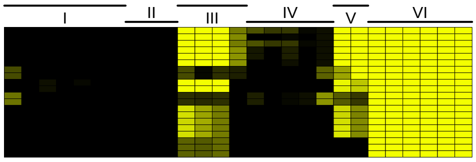

Cluster 34 (n=29)

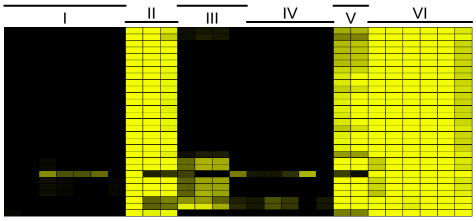

Cluster 35 (n=24)

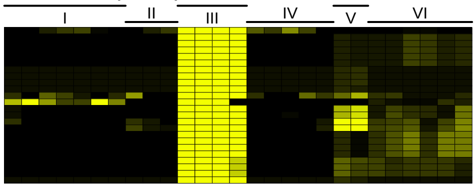

Cluster 36 (n=28)

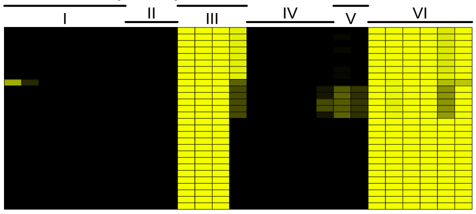

Cluster 37 (n=51)

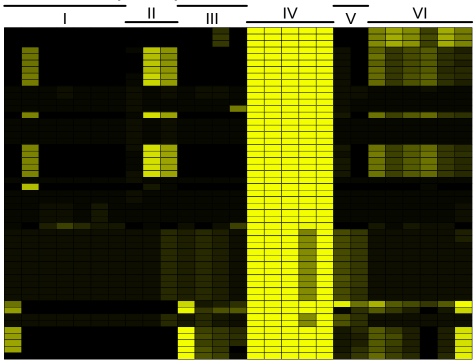

Cluster 38 (n=40)

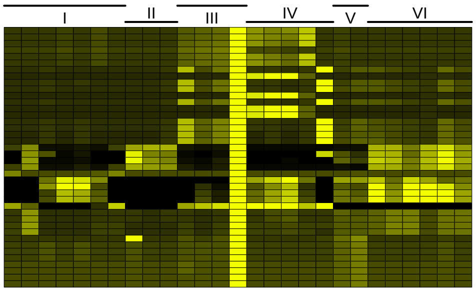

Cluster 39 (n=31)

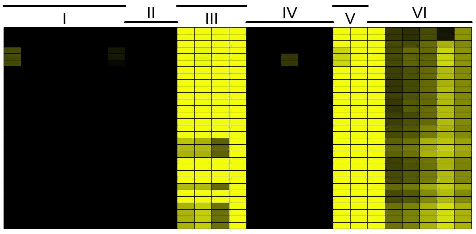

Cluster 40 (n=48)

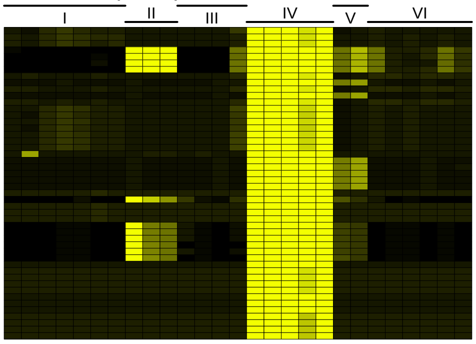

Cluster 41 (n=6)

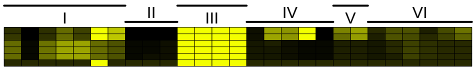

Cluster 42 (n=37)

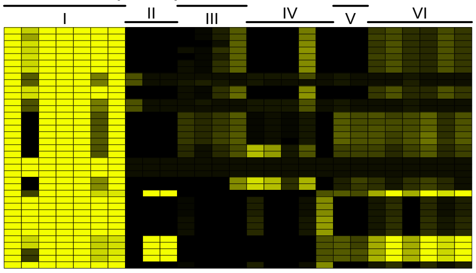

Cluster 43 (n=23)

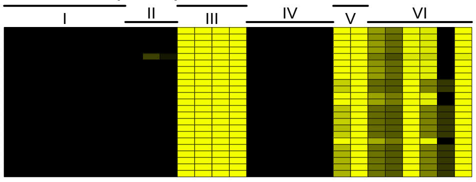

Cluster 44 (n=7)

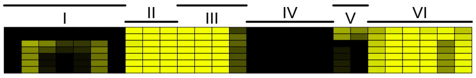

Cluster 45 (n=13)

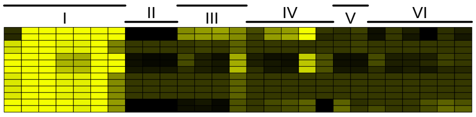

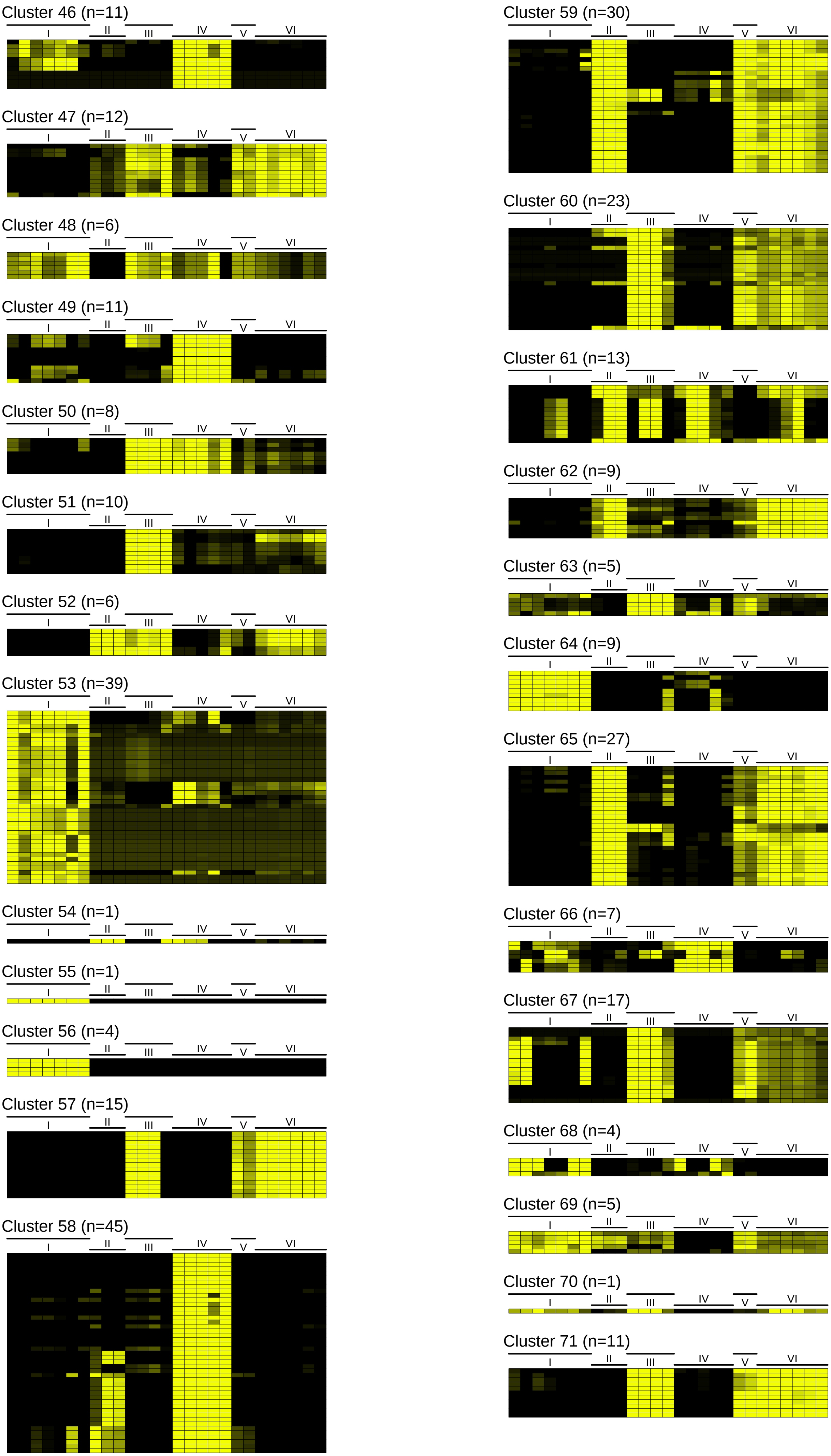

Cluster 46 (n=11)
Cluster 47 (n=12)
Cluster 48 (n=6)
Cluster 49 (n=11)
Cluster 50 (n=8)
Cluster 51 (n=10)
Cluster 52 (n=6)
Cluster 53 (n=39)
Cluster 54 (n=1)
Cluster 55 (n=1)
Cluster 56 (n=4)
Cluster 57 (n=15)
Cluster 58 (n=45)
Cluster 59 (n=30)
Cluster 60 (n=23)
Cluster 61 (n=13)
Cluster 62 (n=9)
Cluster 63 (n=5)
Cluster 64 (n=9)
Cluster 65 (n=27)
Cluster 66 (n=7)
Cluster 67 (n=17)
Cluster 68 (n=4)
Cluster 69 (n=5)
Cluster 70 (n=1)
Cluster 71 (n=11)
I
II
III
IV
V
VI

Cluster 72 (n=37)

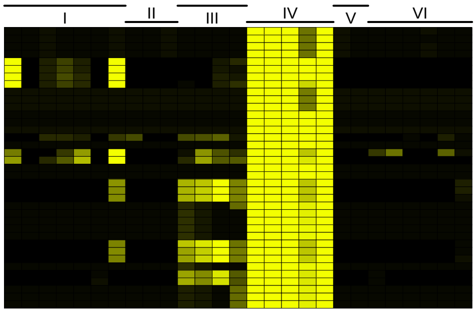

Cluster 73 (n=18)

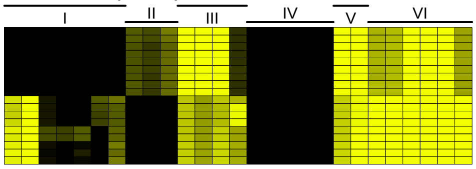

Cluster 74 (n=7)

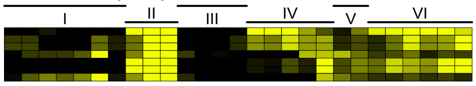

Cluster 75 (n=6)

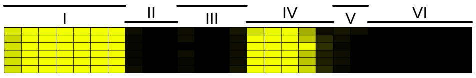

Cluster 76 (n=2)

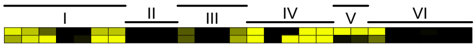

Cluster 77 (n=17)

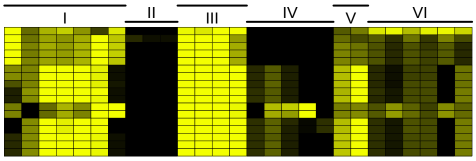

Cluster 78 (n=3)

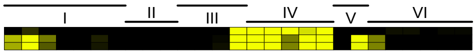

Cluster 79 (n=7)

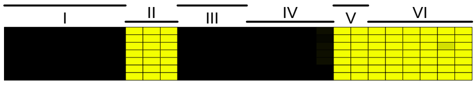

Cluster 80 (n=3)

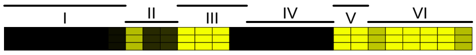

Cluster 81 (n=20)

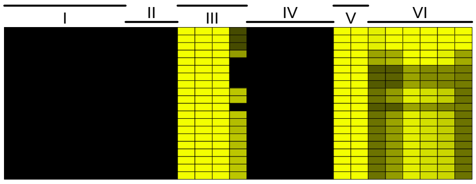

Cluster 82 (n=3)

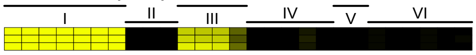

Cluster 83 (n=1)

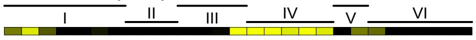

Cluster 84 (n=2)

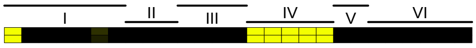

Cluster 85 (n=1)

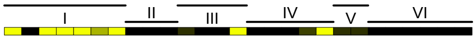

Supplement: Supplementary file 6 [file 1678-8060-mioc-116-e200538-s4.pdf]
